# Comprehensive exploration of immune checkpoint-related genes in the prognosis and tumor immune microenvironment of pancreatic adenocarcinoma

**DOI:** 10.1016/j.clinsp.2024.100481

**Published:** 2024-08-28

**Authors:** Xiao Chen, Herui Zhang

**Affiliations:** a Department of Surgery, Suzhou Hospital of Anhui Medical University, Suzhou, PR China; b Department of Gastroenterology, Suzhou Hospital of Anhui Medical University, Suzhou, PR China

**Keywords:** Pancreatic adenocarcinoma, Immune checkpoint, Tumor immune microenvironment, Prognosis, Biomarker

## Abstract

**Background:**

To comprehensively analyze the clinical significance of Immune Checkpoint-Related Genes (ICRGs) in Pancreatic Adenocarcinoma (PAAD).

**Method:**

PAAD tissues and normal pancreatic tissues were obtained from The Cancer Genome Atlas (TCGA) and Genotype-Tissue Expression (GTEx) databases, and 283 ICRGs were integrated by the Kyoto Encyclopedia of Genes and Genomes (KEGG) and Reactome datasets. Unsupervised clustering was used to obtain potential ICRGs-based PAAD subtypes. Wilcoxon test was performed to screen Differentially Expressed ICRGs (DEICRGs), while cox regression analyses were utilized to identify prognosis-related ICRGs and clinicopathological factors, and construct the corresponding models. The Tumor Immune Microenvironment (TIME) was evaluated. Moreover, the authors performed enrichment analysis, Gene Set Enrichment Analysis (GSEA), and transcription factor regulatory networks to realize underlying mechanisms.

**Results:**

Three ICRGs-based PAAD subtypes were identified, and they were associated with three ESTIMATE scores, a Tumor Microenvironment (TMB) score, 14 therapeutic immune checkpoints, and infiltration levels of seven immune cells. On top of that, the authors constructed two signatures based on DEICRGs to predict the Overall Survival (OS) (Area Under the ROC Curve [AUC: 0.741∼0.778]) and Progression-Free Survival (PFS) (AUC: 0.746∼0.831) of patients. Two nomograms were established by combining clinical variables and signatures. In addition, the authors found higher infiltration of naïve B cells and CD8^+^ T-cells in low-risk PAAD patients, and higher infiltration of suppressive immune cells and cancer-related signaling pathways in high-risk PAAD patients.

**Conclusion:**

The present study suggested that ICRGs were associated with TIME formation and prognosis of PAAD patients, which may serve as novel clinical biomarkers and therapeutic targets.

## Highlights

• Pancreatic Adenocarcinoma (PAAD) exhibits a universally poor prognosis.
• Tumor Immune Microenvironment (TIME) affected the development of tumor.
• Immune Checkpoint-Related Genes (ICRGs) were associated with TIME formation.
• ICRGs were associated with the prognosis of PAAD.
• ICRGs may serve as novel clinical biomarkers and therapeutic targets.

## Introduction

Pancreatic Adenocarcinoma (PAAD) exhibits a universally poor prognosis, which causes almost as many deaths (466000) as incidences (496000) in 185 countries in 2020.^1^ Given the steadily increasing rate of this disease,^2^ it is estimated that PAAD will overtake breast cancer as the third leading tumor-related fatal disease by 2025. Due to the lack of early satisfactory screening methods and atypical symptoms in patients with localized PAAD in the early stage, most patients are diagnosed with an advanced stage upon detection, with only a 6 %‒8 % 5-year survival rate.^3^, ^4^, ^5^ Therefore, there is an urgent demand to find reliable tumor biomarkers that can identify this disease at the early phase and assist in further appropriate therapy.

As for the current therapeutic options, surgical resection combined with chemotherapy and radiotherapy improves the prognostic outcomes of patients with localized PAAD, but the efficacy remains disappointing for advanced patients with distant metastasis.^6^ Increasing evidence indicates that immune checkpoint blockade is one of the most promising therapies for cancer.^7^ Immune checkpoints are some inhibitory pathways, which have major effects on maintaining self-tolerance and regulating the immune response of peripheral tissues to reduce tissue damage.^8^ In the Tumor Immune Microenvironment (TIME), by activating multiple immune checkpoints, tumor cells can evade immune surveillance, grow further, and even invade distant sites.^9^ Immune Checkpoint Inhibitors (ICIs) can intercept co-inhibitory signaling pathways in TIME, and facilitate immune cell activation-mediated tumor cell clearance, which has been shown to be effective in many tumors, including lung cancer, bladder cancer, and melanoma.^10^, ^11^, ^12^, ^13^ However, due to the unique immunosuppressive TIME of PAAD, single-agent ICI treatments have generally not been satisfactory,^14^ which is also related to the fact that there are relatively few ICIs available for treating PAAD. Given that the increasing number of immune checkpoints has been identified, it is crucial to clarify the clinical significance of immune checkpoints in PAAD, explore suitable targeted ICIs, and propose appropriate strategies to aid immunotherapy against PAAD.

Therefore, this study was conducted to evaluate the prognostic and clinical value of Immune Checkpoint-Related Genes (ICRGs) and explore underlying related molecular subtypes. On the clinical side, the authors screened patients suitable for ICI treatments and constructed signatures and nomograms for the prediction of the Overall Survival (OS) and Progression-Free Survival (PFS) of PAAD. On the mechanistic side, the authors explored the relationship between ICRGs and various TIME features in PAAD patients and constructed two ICRGs-Transcription Factors (TFs) regulatory networks.

### Materials and methods

This present research is an observational study and follows the STROBE Statement. The ethical approval is not applicable, because the Cancer Genome Atlas (TCGA) and ICGC belong to public databases, the patients involved in the database have obtained ethical approval, users can download relevant data for free for research and publish relevant articles, and this study is based on open-source data, and the Suzhou Hospital of Anhui Medical University do not require research using publicly available data to be submitted for review to their ethics committee, so there are no ethical issues and other conflicts of interest.

### Data collection

RNA-Sequencing (RNA-Seq) data and clinical information of 178 PADD patients were obtained from The Cancer Genome Atlas (TCGA) database (https://portal.gdc.cancer.gov), and the RNA-Seq data of 165 normal pancreatic tissues was downloaded from the Genotype Tissue Expression (GTEx) database. Fragments per Kilobase Per Million (FPKM) values of the RNA-Seq data presented above were normalized to log2 (FPKM+1), and the authors performed batch effect correction for the two datasets. After excluding one patient with no survival information, the authors included 177 PAAD patients and 165 normal tissues for further study. Moreover, a total of 283 ICRGs were obtained from the Kyoto Encyclopedia of Genes and Genomes (KEGG) database (https://www.kegg.jp/) and the Reactome pathway database (https://www.reactome.org/) (Supplementary Table 1). Finally, 129 candidate ICRGs expressed in both databases were screened.

### Unsupervised clustering for identifying ICRGs-based molecular subtypes in PAAD patients

Based on the 129 candidate ICRGs, the K-means clustering algorithm was used to classify 177 patients with PAAD. The optimal number of clusters was identified by the elbow method and the gap statistic. Principal Component Analysis (PCA) was conducted to verify the distribution differences among subtypes. Subsequently, in order to probe the association between the ICRGs expression levels and PAAD prognosis, a log-rank test was conducted to assess the OS and PFS in different subtypes. Additionally, Kaplan-Meier (KM) survival curves were drawn to illustrate the differences.

To further investigate the underlying mechanisms between different PAAD subtypes, the authors compared the TIME scores and the infiltration levels of 22 immune cells with ESTIMATE and CIBERSORT algorithms. Furthermore, the Tumor Mutation Burden (TMB) score and the expression of 15 therapeutic immune checkpoints in different subtypes were assessed using the Kruskal-Wallis test.

### Establishment of two DEICRGs-based signatures for prognosis evaluation

Differentially Expressed ICRGs (DEICRGs) between PAAD and normal pancreatic tissues were screened by the Wilcoxon test, and a False Discovery Rate (FDR) < 0.05 was regarded as statistical significance.

To further explore the prognostic value of DEICRGs, the authors conducted univariate cox regression to screen significant OS- and PFS-related DEICRGs. Then, genes with p < 0.05 were incorporated into the LASSO analysis to prevent over-fitting. Furthermore, according to the smallest Akaike Information Criterion (AIC) value,^15^ OS- and PFS-related DEICRGs determined by LASSO analysis were included in the multivariate cox analysis to obtain the corresponding optimal model, respectively. Finally, by combining the coefficients and the expression of selected genes, risk score was determined, and the formula was as follows: $\text{Risk}\;\text{score}=\sum_{i}^{n}\beta i*\text{Gi}$.

In the above formula, ‘βi’ represents the regression coefficient of each prognostic DEICRG identified by the multivariate cox regression analysis, and ‘Gi’ shows the expression level of the selected prognostic DEICRG.

In this research, the authors developed OS- and PFS-related signatures to comprehensively evaluate the prognosis of PAAD. According to the median value of the risk score, all PAAD patients were classified into two groups: a high-risk group and a low-risk group. To assess the performance of the signatures, the authors performed a log-rank test and drawn KM survival curves to compare the different survival statuses between the groups. Then, the authors generated receiver operating characteristic (ROC) curves and calculated Area Under the ROC Curve (AUC) values for 1-, 2-, and 3-years.

### Underlying mechanisms and immune features between high- and low-risk groups

The Wilcoxon test suggested that genes with FDR < 0.05 and |log2 fold change (log2FC)| > 1 were considered as Differentially Expressed Genes (DEGs) between high- and low-risk groups, and heat-maps were drawn to demonstrate this. To explore the underlying mechanisms and functions between different risk groups, Gene Ontology (GO) functional annotation and KEGG pathway enrichment analyses were presented, respectively. Meanwhile, Gene set Enrichment Analysis (GSEA) was used to reveal the different potential biological processes between high- and low-risk groups. Moreover, same as before, immune infiltration analysis was conducted to elucidate the infiltration levels of 22 immune cells between high- and low-risk groups using the Wilcoxon test, and the expression levels of 15 therapeutic immune checkpoints were also evaluated.

### Mutation state of hub genes and infiltration level of six immune cells

16 independent prognostic DEICRGs were included in this analysis, including 4 in OS-related signature, 10 in PFS-related signature, and 2 overlapping genes. The potential relationships between the mutational status of 16 hub genes and the level of six representative immune cells in TIME were investigated and visualized in the TIMER website (https://cistrome.shinyapps.io/timer).

### Construction of ICRGs-transcription factors (TFs) regulatory networks

The Transcription Factor (TF) set and corresponding information were obtained from the Cistrome Cancer (http://cistrome.org/CistromeCancer/). Differentially Expressed TFs (DETFs) were determined by matching 274 TFs to DEGs in PAAD and normal pancreas tissues. On top of that, univariate cox analysis was performed to reveal OS- and PFS-related DETFs. Then, Pearson correlation analysis was implemented to measure the correlation between DETFs and DEICRGs, and the results with *r* > 0.4 and p < 0.01 were considered to be reliable. Finally, the above robust DEICRs-DETFs pairs were incorporated to construct two immune-related regulatory networks, and Cytoscape was utilized to illuminate the results.

### Comparison of prognostic values of different factors and construction of clinical-ICRGs nomograms

Univariate and multivariate cox regression analyses were conducted to evaluate the prognostic values of two DEICRGs-based signatures and other clinicopathologic characteristics of patients with PAAD, thereby selecting independent prognostic factors. Additionally, time-dependent ROC curves and corresponding AUC values for 1-, 2- and 3-years were carried out to compare the predictive accuracy of each factor. To more accurately and comprehensively assess the prognosis of PAAD patients, clinical-ICRGs nomograms were applied to visualize the OS and PFS. According to the independent prognostic factors acquired by multivariate analysis, two clinical-ICRGs nomograms were developed for predicting the PAAD OS and PFS. Meanwhile, the Concordance index (C-index) was calculated, and the calibration plot was drawn to estimate the performance of the two nomograms.

### Statistical analysis

In this study, all statistical analyses and figures were accomplished with SPSS 21.0 and R software (version 4.0.2). p-value < 0.05 was considered statistically significant. The batch effect correction between data was performed using “limma” package. Univariate and multivariate cox analyses were conducted by the “survival” package, and LASSO analysis was generated using “glmnet” package. “Survminer” and “survivalROC” packages were implemented to draw KM survival curves and time-dependent ROC curves. Heat-map and volcano maps were drawn with “pheatmap” package, and the nomogram and the calibration curve were visualized by the “rms”, “regplot”, and “survival” packages.

## Results

### Overview of characteristics of PAAD patients

**Fig. 1:**
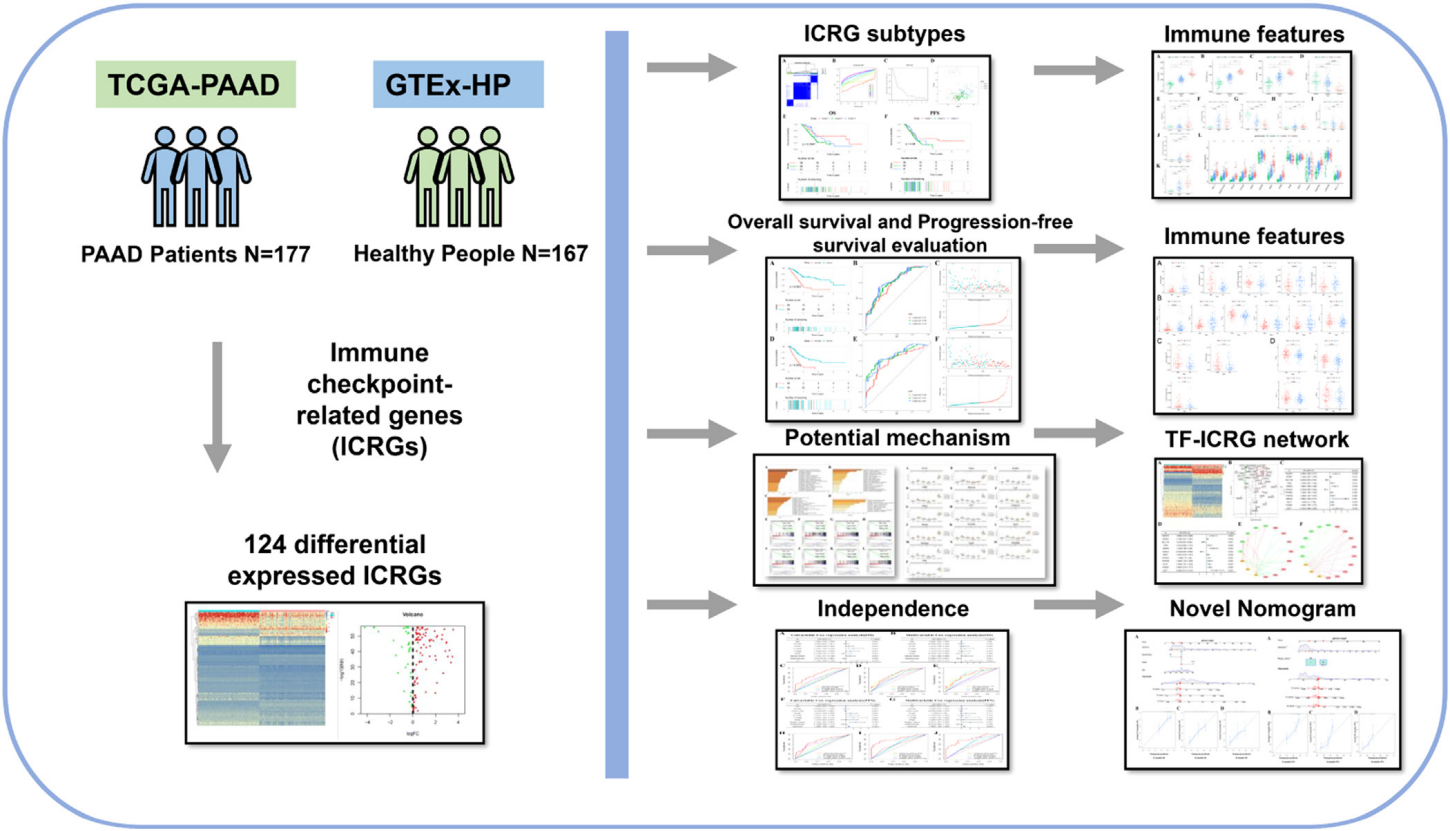
The workflow of this study.

The present study was performed to evaluate the prognostic and clinical value of ICRGs and explore underlying related molecular subtypes (Fig. 1). The clinicopathologic characteristics of PAAD patients are described in Supplementary Table 2. The average age was 64.68 ± 0.81 years old, and the proportion of man (54.8 %) and female (45.2 %) cases were similar. The median OS time of patients with PAAD was 1.663 years (95 % CI 1.418‒1.908), and the median PFS time was 1.332 years (95 % CI 1.111‒1.552).

### Three ICRGs-based PAAD subtypes were significantly related to the prognosis and TIME characteristics

**Fig. 2:**
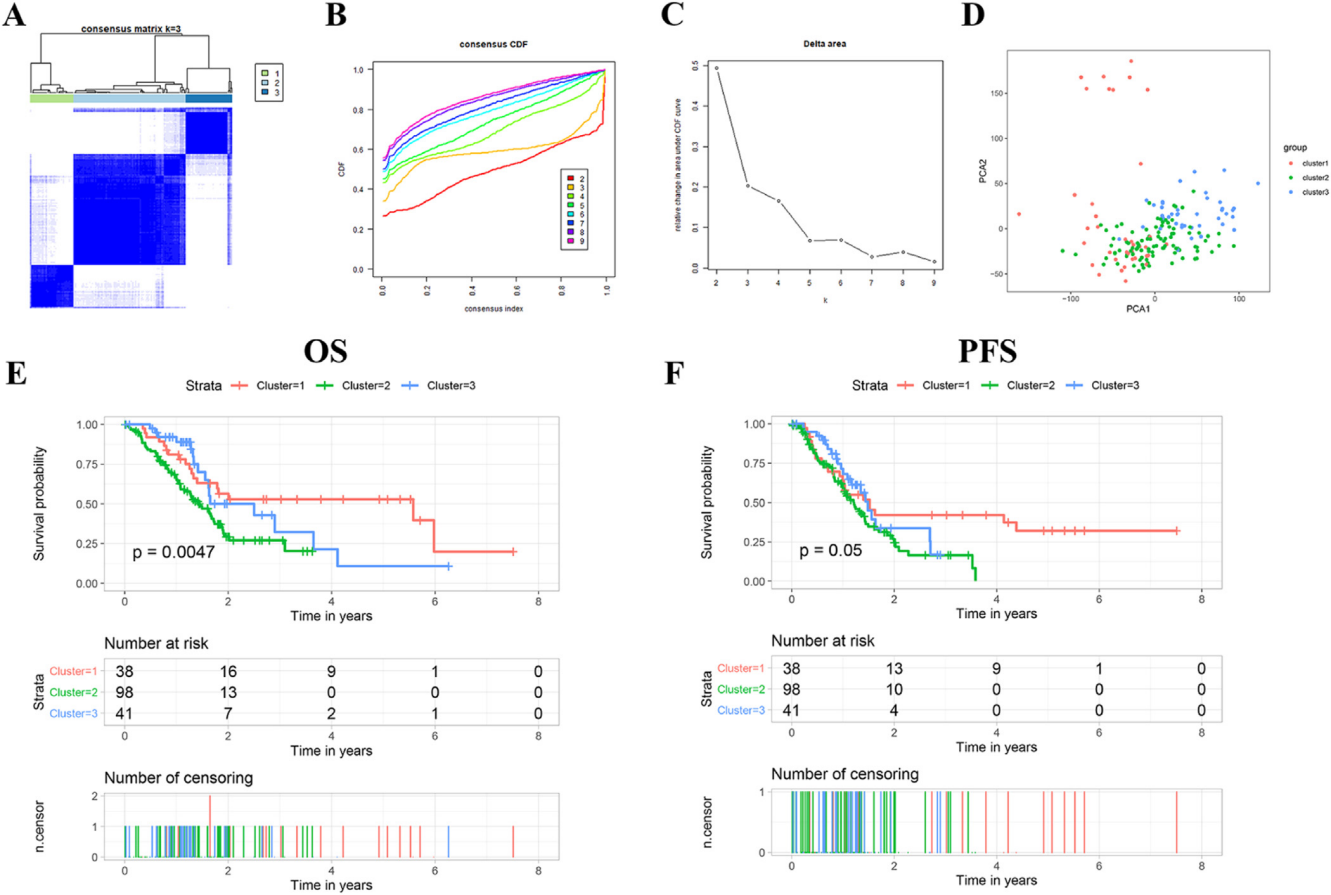
**Identification and verification of ICRGs-based PAAD subtypes**. (A‒C) Unsupervised clustering divided PAAD patients into three clusters. (D) PCA demonstrated distant heterogeneity among the three PAAD subtypes. (E‒F) K-M survival analysis of OS and PFS status of PAAD patients in three subtypes. PAAD, Pancreatic adenocarcinoma; ICRGs, Immune Checkpoint-Related Genes; PCA, Principal Component Analysis; OS, Overall Survival; PFS, Progression-Free Survival.

The ICRGs profiling suggested that ICRGs were dramatically heterogeneous among patients with PAAD, and this uncovered that its intrinsic characteristics were of great clinical relevance. According to the results of unsupervised clustering (Fig. 2 A‒C), the authors identified three discernible PAAD subtypes. Three ICRGs-based PAAD subtypes were determined as follows (Fig. 2A): Cluster 1 (n = 38, 21.5 %), Cluster 2 (n = 98, 55.4 %) and Cluster 3 (n = 41, 23.1 %). Then, the result of PCA based on full RNA-Seq data also demonstrated distant heterogeneity among the three PAAD subtypes (Fig. 2D). KM curves which performed log-rank test indicated that PAAD patients with different subtypes had significantly different OS (p = 0.0047) and PFS time (p = 0.05), and patients with Cluster 2 presented the best prognosis (Fig. 2 E‒F).

**Fig. 3:**
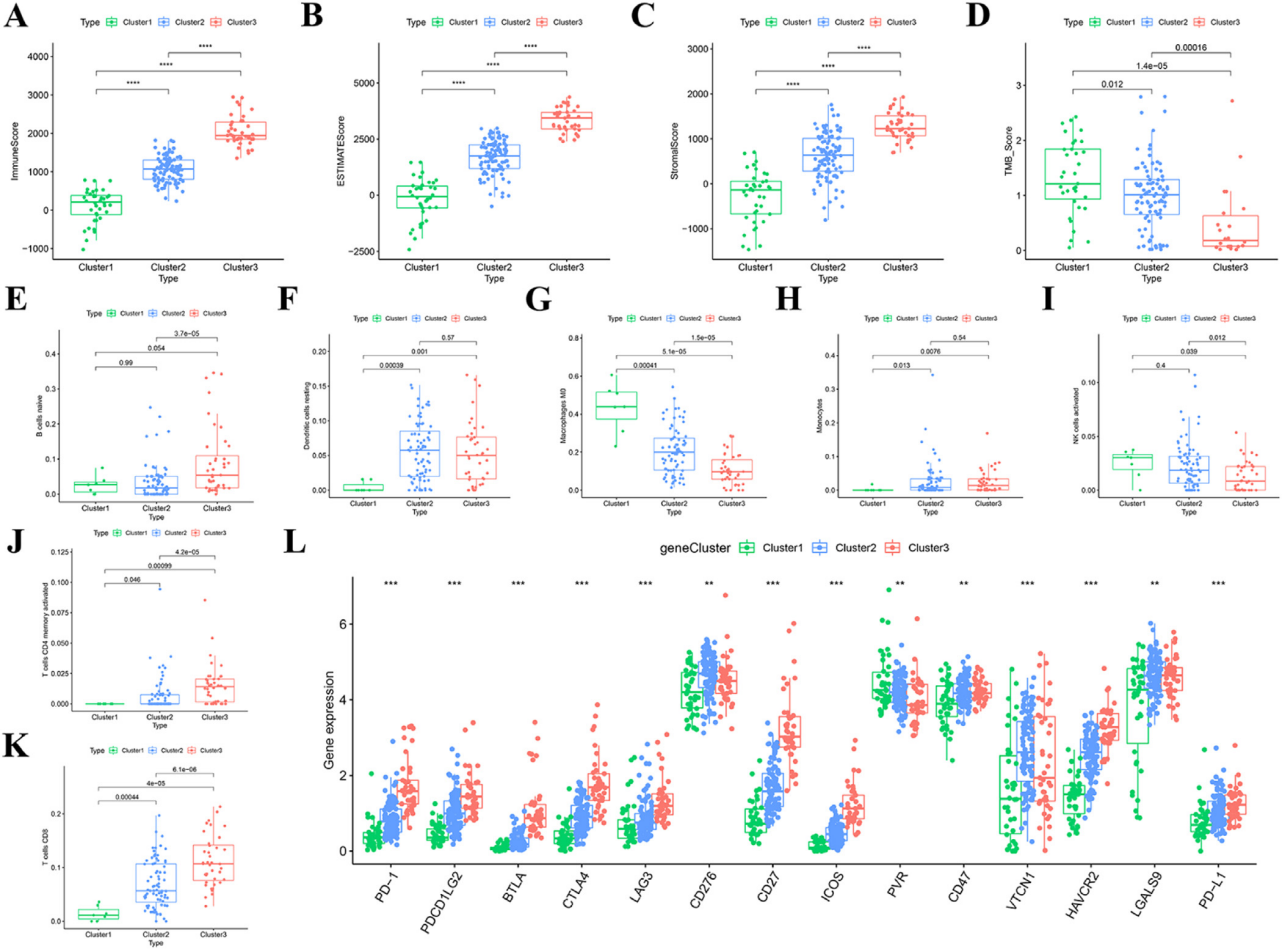
**TIME characteristics, TMB, and immune cells among different PAAD subtypes**. (A‒C) Comparison of three TIME scores between the three clusters. (D) Comparison of the TMB score between the three clusters. (E‒K) The infiltration level of seven immune cells in different groups. (L) The expression level of 15 immune checkpoints in three clusters. TIME, Tumor Immune Microenvironment; TMB, Tumor Mutation Burden.

To further investigate the potential mechanism among patients with different subtypes, the authors compared the TIME and TMB scores. As seen in Figure 3 A‒C, the authors found Cluster 1 had the lowest stromal, immune, and estimate scores, while patients with Cluster 3 had the highest scores. Additionally, diametrically opposite results were obtained from the TMB analysis; Cluster 1 had the highest score; and Cluster 3 had the lowest score (Fig. 3D). Moreover, the authors evaluated the levels of 22 kinds of immune cells to better understand the correlation between ICRGs and immune infiltration. B-cells naïve, T-cells CD8, T-cells CD4 memory activated, NK cells activated, monocytes, macrophages M0, dendritic cells resting, and mast cells activated were differentially expressed among different PAAD subtypes (Fig. 3 E‒K). Furthermore, among 15 therapeutic immune checkpoints, the authors found 14 checkpoints were distantly distributed across subtypes, including PD-1, PDCD1LG2, BTLA, CTLA4, LAG3, CD276, CD27, ICOS, PVR, CD47, VTCN1, HAVCR2, LGALS9 and PD-L1 (Fig. 3L). These results indicated that ICRGs based PAAD subtypes presented discernible TIME characteristics, which may help evaluate and guide ICIs therapy.

### Developing two ICRGs-signatures for OS and PFS evaluation

Differential expression analysis revealed that 124 of 129 ICRGs were differentially expressed in the PAAD and normal pancreatic tissues (Supplementary Fig. 1), including 91 up-regulated DEICRGs and 33 down-regulated DEICRGs (Supplementary Table 3). Further, univariate cox regression analysis screened 35 OS-related DEICRGs (Supplementary Table 4) and 38 PFS-related DEICRGs (Supplementary Table 5). Then, LASSO analysis ulteriorly identified 14 candidate DEICRGs for OS and 20 candidate DEICRGs for PFS. Finally, based on the smallest AIC value, a six gene OS-related signature (Risk score = 0.46*STAT1+0.99*PAK2+ 0.78*MAPK1-1.75* GRB2-0.74*PPP3CB-1.68*LAT) and a 12 gene PFS-related signature (Risk score = 0.28*STAT1-1.33*EML4-0.97*FYN+0.75*CSNK2A1-0.80*

TRAF6-0.67*MAP2K6+0.65*NRAS+1.01*PAK2+0.47*MAP2K3-0.49*PAK3-0.89*PPP2R5B+0.53*EGF) were established. Interestingly, two genes, STAT1 and PAK2, were common independent risk factors for OS and PFS.

**Fig. 4:**
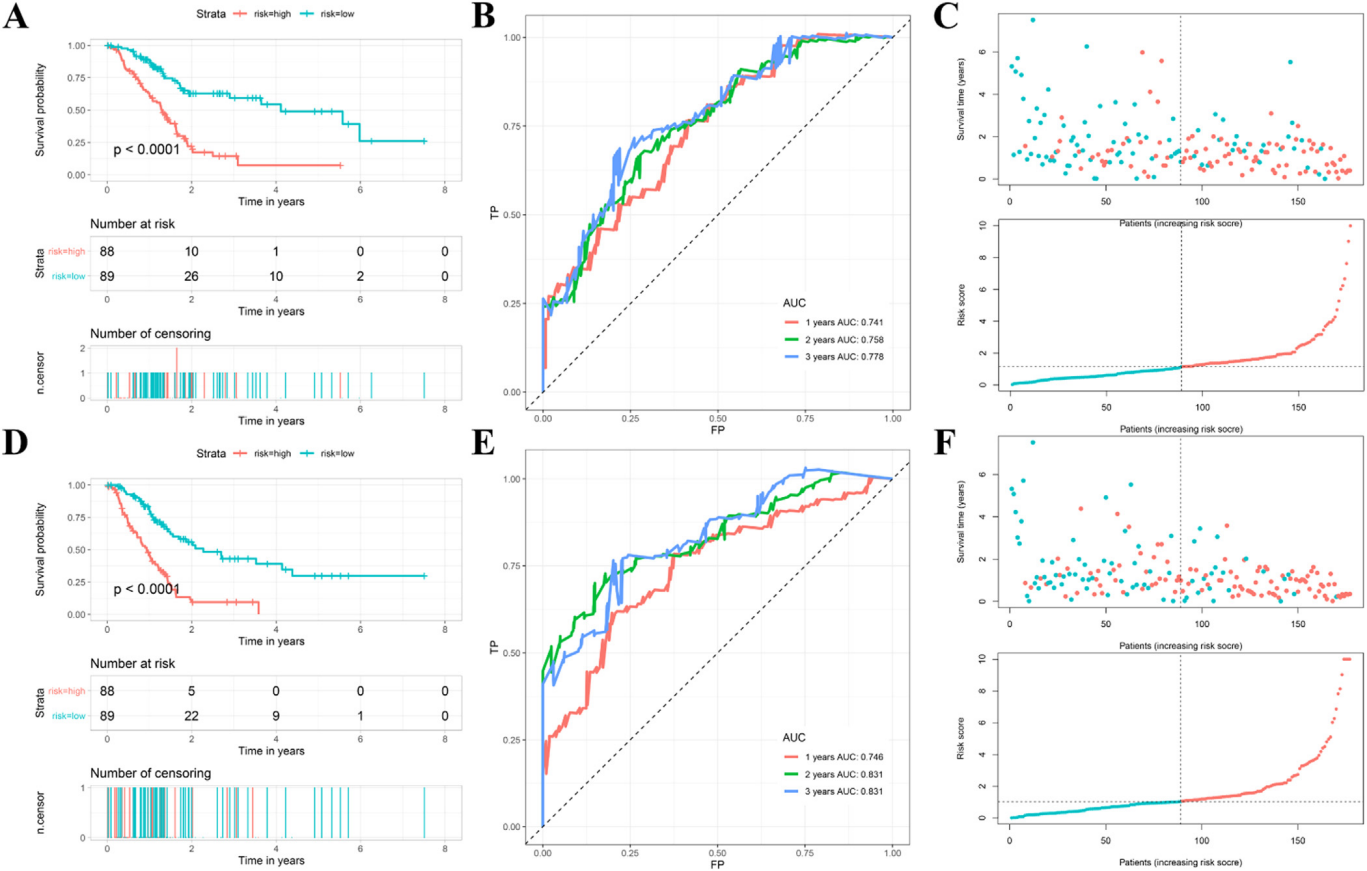
**ICRGs-based prognostic signatures for OS and PFS**. (A, D) Kaplan-Meier (K-M) survival analysis of OS and PFS between high- and low-risk groups. (B, E) ROC curves of OS-related signature and PFS-related signature at 1-, 2- and 3-years. (C, F) Scatter plots of survival time and risk score for PAAD patients in different risk groups. ICRGs, Immune Checkpoint-Related Genes; OS, Overall Survival; PFS, Progression-Free Survival; PAAD, Pancreatic Adenocarcinoma.

The median risk score was used to classify patients into the high- and low-risk groups and KM survival curves showed that high-risk PAAD patients had lower OS and PFS time (Fig. 4 A and D). In addition, OS and PFS survival status of patients with high- and low-risk PAAD were visualized via scatter plots, as shown in Figure 4C and F. Furthermore, the AUC values of time-dependent ROC were 0.741, 0.758, and 0.778 for OS, and 0.746, 0.831 and 0.831 for PFS (> 0.7), respectively (Fig. 4B and E). Thus, the above analyses suggested that DEICRGs-based signatures possessed a stable and robust predictive prognosis ability.

To examine whether the OS- and PFS-related signatures have similar prognostic values in the different data sets, RNA-sequencing of 260 PAAD patients obtained from the ICGC database (PACA-AU and PACA-CA) (https://dcc.icgc.org/) was served as validation cohort. The two datasets were merged with “limma” package, the expressions of six OS-related and 12 PFS-related DEIRGs were extracted from the validation cohort, and the corresponding risk score of each patient was calculated. Based on the median risk score, the authors found high-risk PAAD patients in the OS-related signature and PFS-related signature both exhibited worse prognoses (Supplementary Fig. 2A‒B).

### Underlying mechanisms and TIME characteristics between high- and low-risk groups

**Fig. 5:**
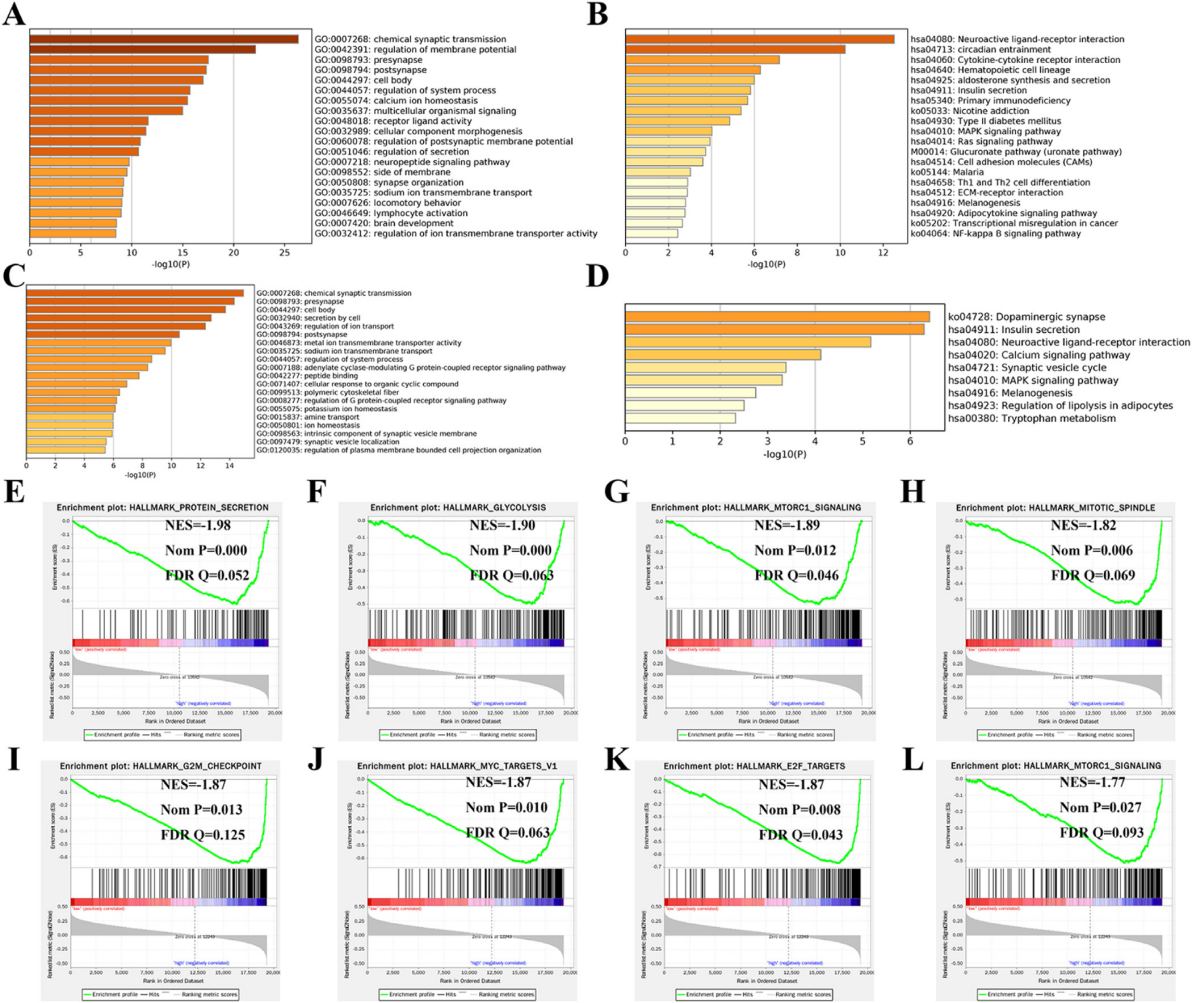
**Functional annotations and GSEA between high- and low-risk groups**. (A‒B) GO and KEGG analyses between different risk groups for OS. (C‒D) GO and KEGG analyses between different risk groups for PFS. (E‒H) Four significant cancer hallmarkers were higher expressed in the high-risk group for OS. (I‒L) Four significant cancer hallmarkers were higher expressed in the high-risk group for PFS.

**Fig. 6:**
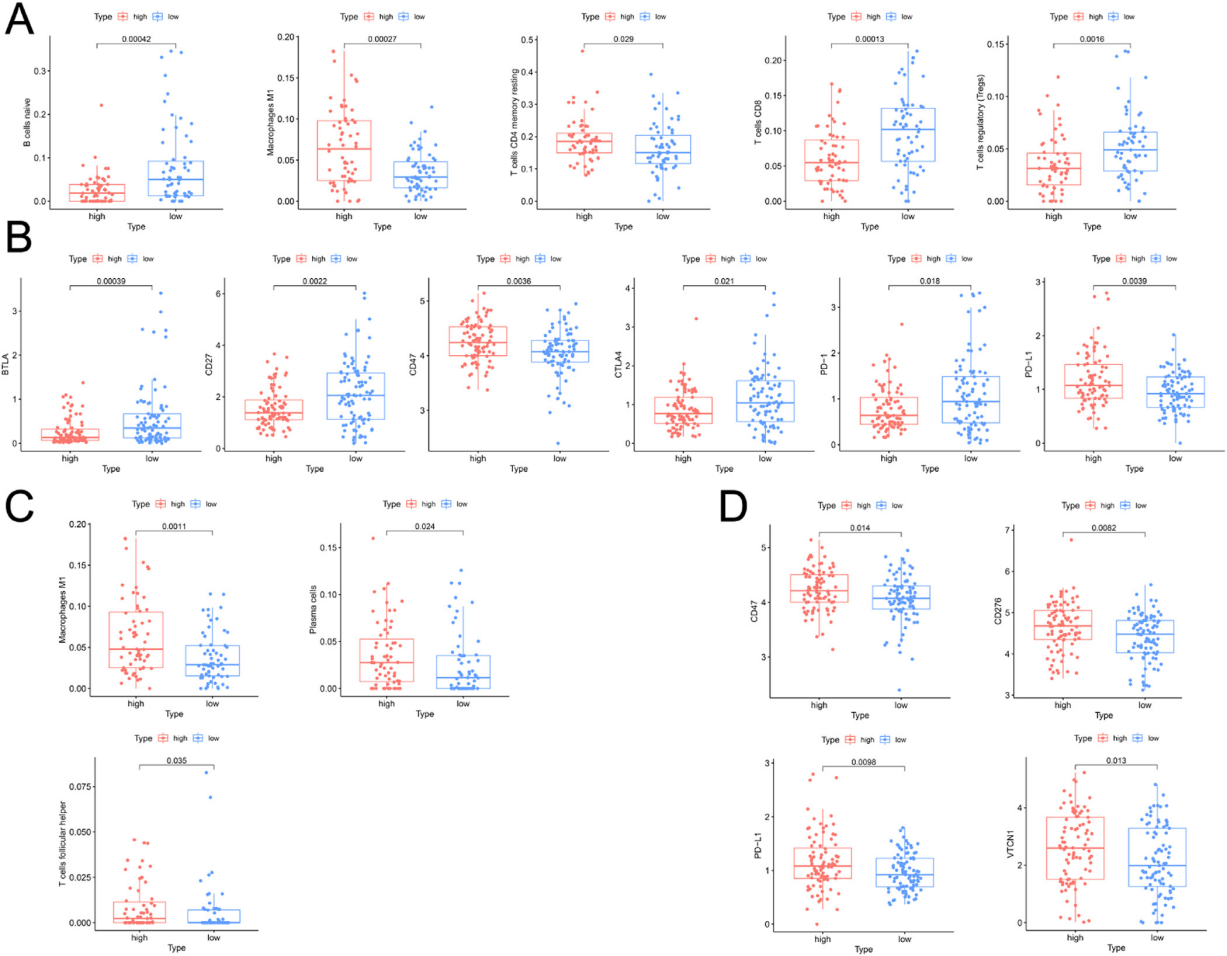
**TIME characteristics between high- and low-risk groups**. (A) The infiltration level of five significant immune cells between high and low-risk groups for OS. (B) The expression of six significant immune checkpoints for OS. (C) The infiltration level of three significant immune cells between high and low-risk groups for PFS. (D) The expression of four significant immune checkpoints for PFS.

Nine hundred and twenty DEGs (Supplementary Table 6) and 492 DEGs (Supplementary Table 7) between high- and low-risk groups were found in the OS-related signature and PFS-related signature (Supplementary Fig. 3A and B), respectively. The GO results indicated that OS-related DEGs were enriched in chemical synaptic transmission, regulation of membrane potential, presynapse, postsynapse, and cell body (Fig. 5A), PFS-related DEGs were enriched in chemical synaptic transmission, presynapse, cell body, secretion by cell, and regulation of ion transport (Fig. 5C). In addition, the top five terms of KEGG analysis were neuroactive ligand-receptor interaction, circadian entrainment, cytokine-cytokine receptor interaction, hematopoietic cell lineage, and aldosterone synthesis and secretion for OS (Fig. 5B), and dopaminergic synapse, insulin secretion, neuroactive ligand-receptor interaction, calcium signaling pathway, and synaptic vesicle cycle for PFS (Fig. 5D). Moreover, GSEA identified that cancer markers were significantly higher in the high-risk group, including protein secretion, glycolysis, MTORC1 signaling, and mitotic spindle for OS (Fig. 5E‒H), and G2M checkpoint, MYC targets, E2F targets, and MTORC1 signaling for PFS (Fig. 5I‒L). From the perspective of immunology, the authors found patients with low-risk PAAD in the OS-related signature had higher infiltration levels of B-cells naïve, CD8 T-cells, and T-cells regulatory, and low infiltration levels of Macrophages M1 and CD4 memory cells (Fig. 6A). Similarly, patients with high-risk PAAD in the PFS-related signature presented higher infiltration levels of Macrophages M1, plasma cells and follicular helper T-cells (Fig. 6C). Additionally, PAAD patients in different risk groups also shown differences in crucial immune checkpoints expression, which indicated the different sensitivities to immunotherapies. Patients with low-risk PAAD presented markedly higher expression levels of BTLA, CD27, CTLA4, and PD-1, and lower expression levels of CD47, PD-L1, CD276, and VTCN1 (Fig. 6B, D).

### Mutation state of hub genes and infiltration level of immune cells

In the present study, four OS-related ICRGs, 10 PFS-related ICRGs, and two overlapping genes were included for mutation analysis. The authors further investigated whether the copy number of crucial ICRGs could have reasonable effects on the infiltration levels mediated by six effector immune cells, and obtained results with significant correlation (Supplementary Fig. 4 A‒P). For example, copy number purpose deletion of MAPK1 led to reduced infiltration levels of immune cells (Supplementary Fig. 4C). Additionally, both high and low copies of PPP3CB resulted in the reduced expression of effector cells, which indicated that suppressor gene mutation could increase the immune response and further fight against cancer (Supplementary Fig. 4E). Generally, the close association with immune cells illustrated that the two signatures were relatively stable and robust.

### Exploring the regulatory mechanism of ICRGs

**Fig. 7:**
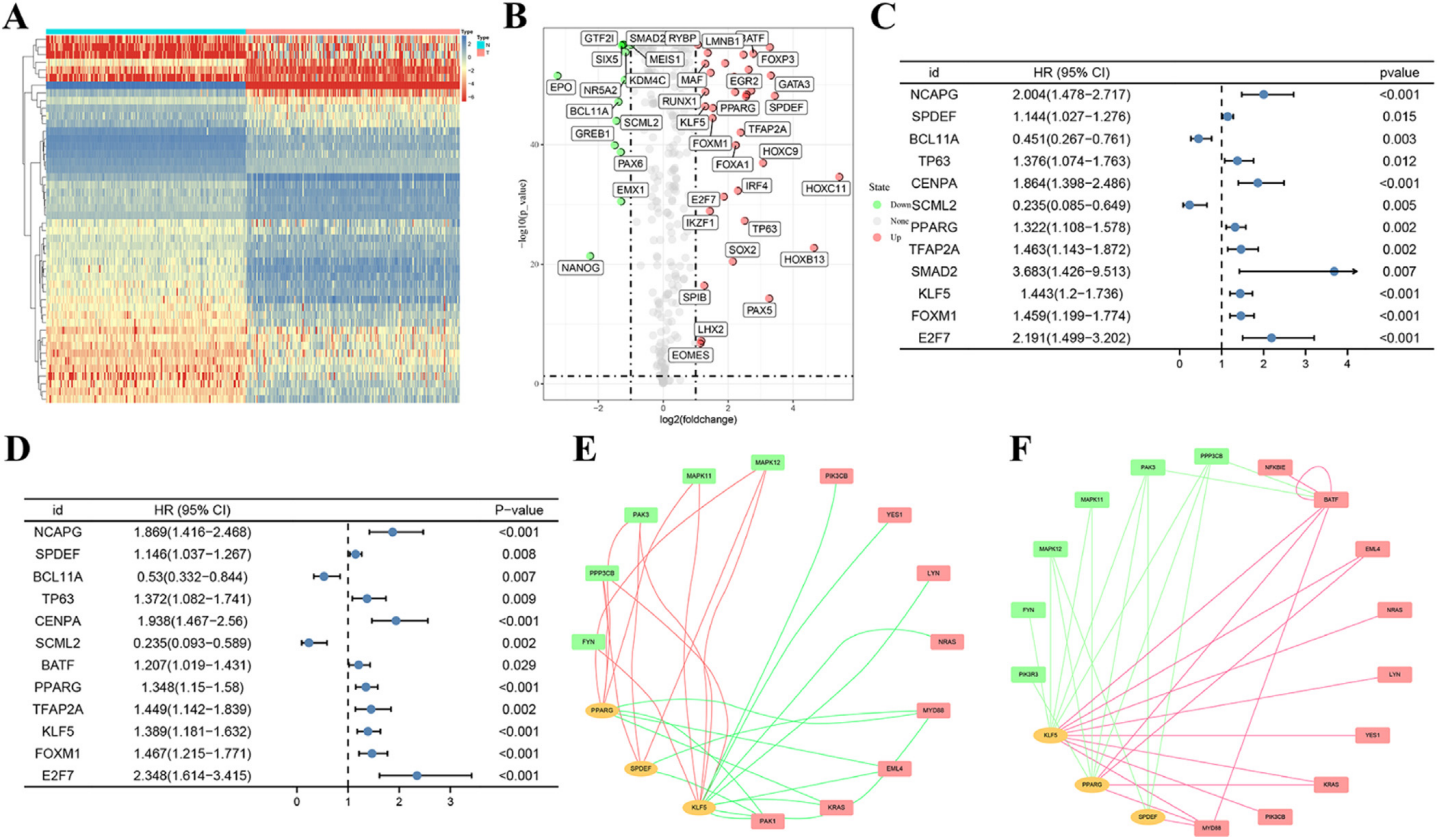
**Two DEICRs-DETFs regulatory networks**. (A‒B) Heatmap and volcano plot of 45 DETFs in PAAD and normal pancreas tissues. (C‒D) 12 OS-related DETFs and 12 PFS-related DETFs were determined by the univariate cox analysis. (E, F) Regulatory network of OS (PFS) -related DEIRCGs and corresponding DETFs.

Of the 247 TFs, 48 DETFs were determined by matching TFs to DEGs, as shown in heat-map (Fig. 7A) and volcano plots (Fig. 7B) (Supplementary Table 8). From the perspective of clinical outcome, the authors found 12 DETFs had some associations with the OS of PAAD patients (Fig. 7C), and 12 DETFs were associated with the PFS of PAAD patients (Fig. 7D). Moreover, three DETFs, including PPARG, SPDEF, and KLF5, were determined to co-express with 13 OS-related DEICRGs (Supplementary Table 9) and 15 PFS-related DEICRGs (Supplementary Table 10). Finally, based on the above results, two DEICRGs-DETFs networks were constructed to illustrate the regulatory mechanism (Fig. 7 E and F).

### Comparison of the prognostic value of different factors and construction of clinical-ICRGs nomograms

**Fig. 8:**
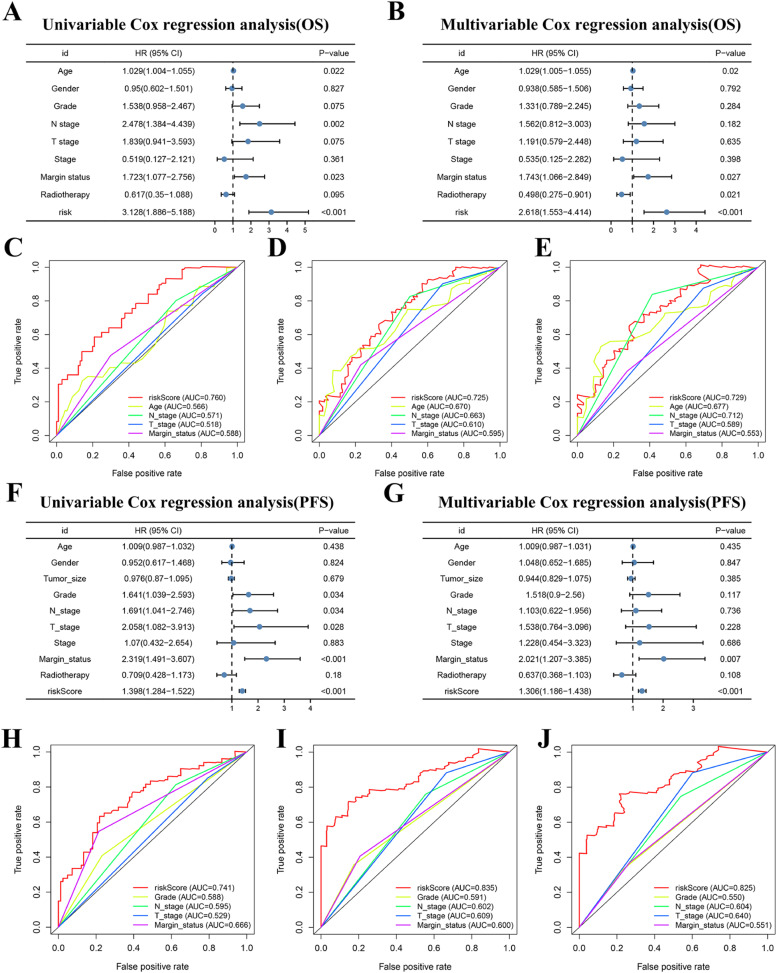
**Identification and comparison of independent prognostic factors**. (A, B) Univariate and multivariate cox analyses of the OS-related signature and clinical variables. (C‒E) Comparison of AUC values between OS-related signature and all significant factors. (F‒G) Univariate and multivariate cox analyses of the OS-related signature and clinical variables. (H‒J) Comparison of AUC values between PFS-related signature and all significant factors.

**Fig. 9:**
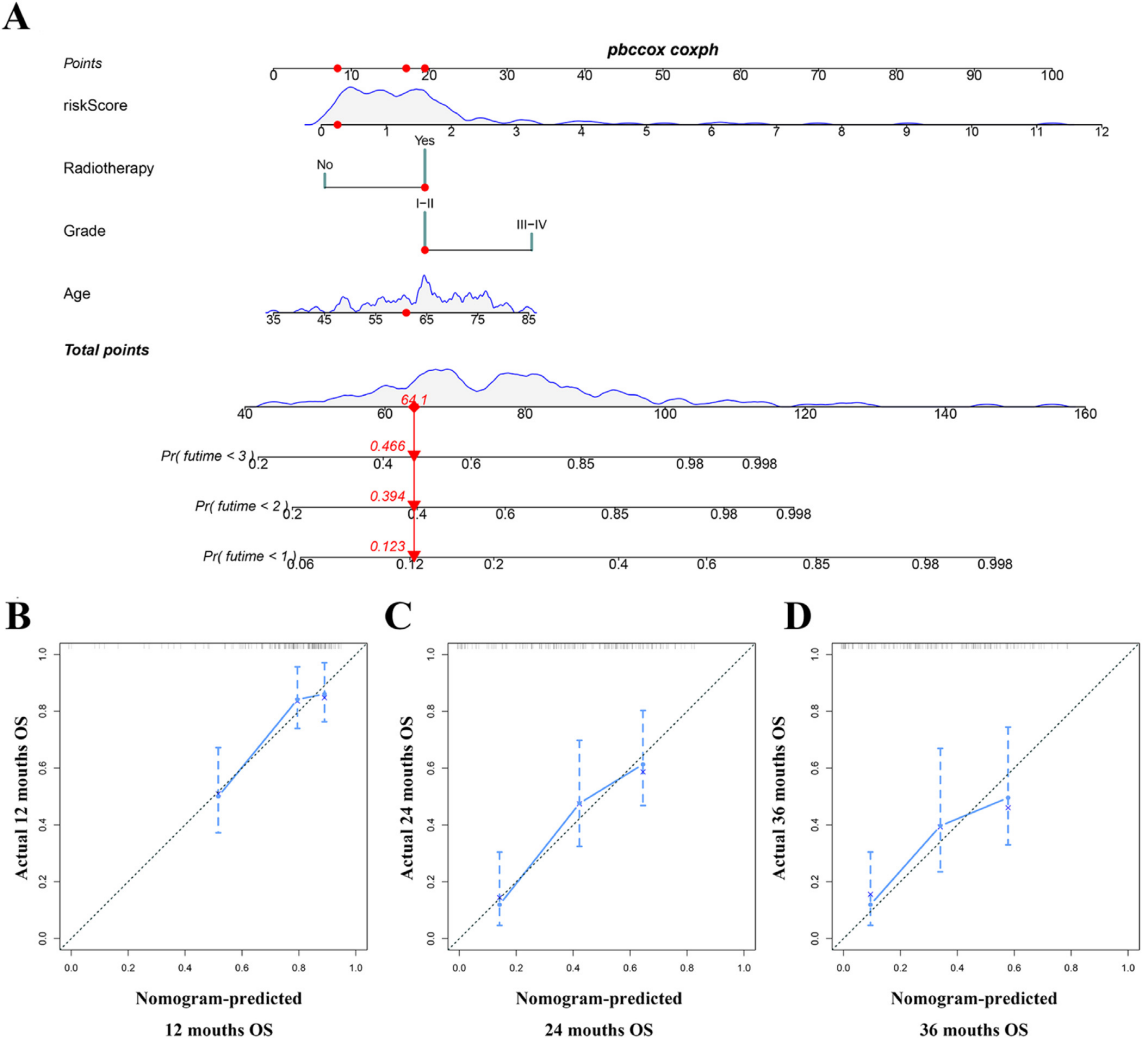
**Establishing an ICRG-clinical nomogram to predict the OS of PAAD patients**. (A) A nomogram combining the OS-related signature, radiotherapy, grade, and age. (B‒D) The calibration curves of the nomogram at 1-, 2- and 3-years.

**Fig. 10:**
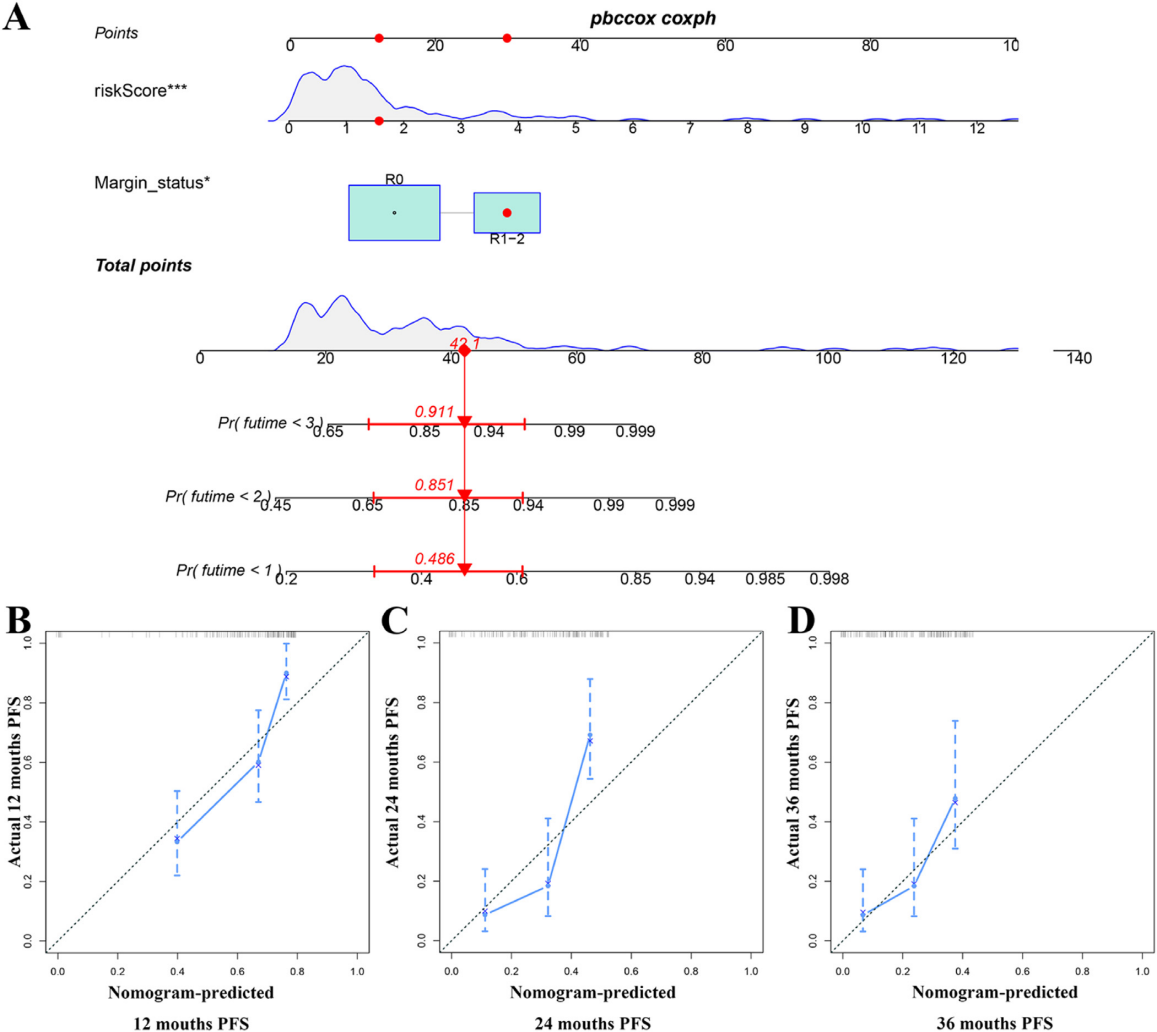
**Developing an ICRG-clinical nomogram to predict the PFS of PAAD patients**. (A) A nomogram combining the PFS-related signature, radiotherapy, grade, and age. (B‒D) The calibration curves of the nomogram at 1-, 2- and 3-years.

Univariate analysis showed that age, N-stage, margin status, and ICRGs-based signature were OS-related variables (Fig. 8A), and grade, N-stage, T-stage, margin status, and ICRGs-based signature were PFS-related factors (Fig. 8F). On top of that, multivariate analysis determined that age, margin status, radiotherapy, and ICRGs-based signature were independent factors for OS (Fig. 8B), and margin status and ICRGs-based signature for PFS (Fig. 8G), respectively. The authors further compared the prognostic value of the ICRGs-based signature with clinicopathological factors, suggesting that the discrimination of signatures was better than that of all clinical variables at 12, 24, and 36 months for OS (Fig. 8 C‒E) and PFS (Fig. 8H‒J), respectively. On the basis of the above independent factors, two clinical-ICRGs nomograms were constructed to evaluate the OS (Fig. 9A) and PFS (Fig. 10A) of PAAD patients. The C-index of the nomogram was 0.708 (95 % CI 0.641‒0.775) for OS and 0.755 (95 % CI 0.704‒0.806) for PFS. Furthermore, the calibration curves at 12, 24, and 36 months exhibited strong agreement between predicted results and the actual outcomes (Fig. 9B‒D, and Fig. 10B‒D).

## Discussion

PAAD remains an aggressive and highly lethal disease with limited effective treatment tools. Novel targeted therapies and immunotherapeutic strategies are important treatment options in addition to surgery and chemoradiotherapy, but recent clinical trials have shown their very limited application in PAAD, which is associated with the unique biological behavior and TIME.^16^ The immunosuppressive microenvironment of PAAD is extremely heterogeneous, with the presence of a large number of unknow inhibitory tight matrix components to be antagonized, resulting in its difficult reprogramming.^17^^,^^18^ Breaking the physical barrier formed by the extracellular matrix and inducing more effector immune cells are two game-changing solutions.^19^ Therefore, this research aimed to study the predictive and prognostic values of ICRGs for immunotherapy, as well as their relationship with TIME, thereby finding pivotal molecules that could activate the immunosuppressive microenvironment. Three ICRGs-based subtypes, two molecular signatures and nomograms were identified for clinical use. Functional annotation, GESA, TIME, immune infiltration analysis, and ICRG-TF networks were preformed to realize underlying mechanisms.

The selection of early intervention and treatment options for PAAD is primarily based on symptoms, AJCC stage, histological grade, and tumor markers. Integrative analyses indicated that cancer genotyping based on specific genes may be beneficial for identifying suitable patients for corresponding therapies and prognosis evaluation.^20^, ^21^, ^22^ Herein, the authors implemented a machine learning method to determine three ICRGs-based PAAD subtypes with distinct clinical characteristics. Three ICRGs-based clusters as discrete entities showed significant OS and PFS differences. PAAD patients with different subtypes demonstrated distinct TIME scores, with only a few presenting relatively high immune scores, which was consistent with previous reports showing that the cold microenvironment of PAAD was heterogeneous.^3^ TMB remains a predictive biomarker for immunotherapy. Previous reports suggested that TMB influenced the immune infiltration signatures, and high TMB could appeal to effector cells to fight tumor cells,^23^^,^^24^ but this was not the case in PAAD. More specifically, the authors found that the major immune cells enriched in the PAAD subtype with higher TIME scores were antitumor cells, including B-cells naïve, CD4 memory T-cells, and CD8 T-cells. However, such PAAD patients did not present a better prognosis, probably due to the presence of a large amount of suppressive extracellular matrix encapsulating tumor cells in the TIME.^25^ In addition, ICRGs-based PAAD subtypes exhibit differential expression of crucial therapeutic immune checkpoints, which may assist patients in selecting ICI combination therapies. The identification of novel subtypes of PAAD based on ICRGs not only enables accurate assessment of patients' prognosis but also assists doctors in formulating individualized treatment plans and making corresponding adjustments. Different subtypes exhibit distinct levels of immune checkpoint expression and infiltration of immune effector cells, which facilitates the understanding of the immunosuppressive microenvironment in PAAD and the development of new therapeutic targets.

ICRGs showed high predictive value of treatment response and survival evaluation for many cancers. There is a lack of ICRGs-related studies in PAAD. This study established two molecule signatures as valid clinical tools. These two models demonstrated excellent performance in evaluating OS and PFS, which was comparable to existing similar studies.^26^, ^27^, ^28^ However, these models may be better able to assess the efficacy of immunotherapy and patients’ outcomes. Based on this, the ICRGs-clinical nomograms established with traditional clinical features may have great potential for clinical application. The key genes involved are mostly related to the regulation of the immune microenvironment, and the activation and inhibition of critical cancer biological signals. STAT1 and PAK2, two overlapping genes in the two signatures caught the authors’ attention. STAT1 plays a critical role in immunologic self-tolerance and innate immune function.^29^ Particularly, STAT1 has a close association with the development of advanced tumors and the maintenance of stem cells in terms of anti-tumor function.^30^^,^^31^ Varun et al.^32^ found that the inhibition of STAT1 activation partially down-regulated PD-L1, which was frequently expressed in several tumors, and associated with immune escape. The immune features results in this research also showed the high-risk patients had higher expression levels of PD-L1. Interestingly, the high expression of STAT1 led to satisfying OS of ovarian cancer, rectum adenocarcinoma, and sarcoma, whereas in several cancers including pancreatic cancer, and lung adenocarcinoma, the high expression of STAT1 was correlated with poor OS.^29^^,^^31^ More importantly, previous investigation revealed that STAT1 could protect T-cells from NK cell-mediated cytotoxicity.^33^ Nevertheless, the overexpression of STAT1 had been reported to inhibit T-cell expansion, indicating that targeting STAT1 could enhance T-cell numbers.^34^ The p21-Activated Kinases (PAKs) are members of the serine/threonine kinases family involved in cell cycle regulation, neoplastic processes, and inflammation.^35^^,^^36^ PAK2 protein works as a cytokine and phosphorylates Bcl2–Associated Death promoter (BAD) protein, causing inhibition of proapoptotic signaling.^37^ Existing evidence described that PAK2 promoted the growth and metastasis of pancreatic cancer.^38^ The authors speculated that these two genes acted as important immune mediators in the TIME of PAAD, and the molecular signatures based on them may have great clinical translational vale.

Additionally, ICRGs-based molecular models also revealed the immune microenvironment and specific molecular features associated with different outcomes, making the model more interpretable and applicable. Immune infiltration analysis was also conducted for the high- and low-risk groups and it was observed that satisfactory prognosis was associated with higher infiltration level of B-cells naïve, CD8 T-cells, and T-cells regulatory (Tregs), and lower infiltration level of Macrophages M1 and plasma cells, which was in line with the previous study that sufficient effector immune cells could synergistically exert anti-tumor effects in a minority of patients.^39^^,^^40^ Based on the functional annotation and GSEA of DEGs between different risk groups, the authors found that patients with lower survival probability mainly enriched in cancer-related biological processes, including MAPK signaling pathway, RAS signaling pathway, glycolysis, Mtorc1 signaling, G2M checkpoint, MYC targets, and E2F targets. Of course, these hallmark pathways can also regulate immune evasion, and they are involved in a highly complex network of relationships. Finally, two ICRGs-TFs regulatory networks were developed to elucidate the transcriptional mechanism of ICRGs, further understanding the expression of ICRGs and the molecular mechanisms underlying PAAD, which provided new perspectives for studying immunosuppressive microenvironment targets and resistance mechanisms.

It is undeniable that some limitations still exist in this study. Firstly, the present study was a retrospective analysis and had a small sample size with unavoidable bias. Secondly, the current findings of the present study only resulted from the bioinformatic analysis, and there are not clinical and experimental trials to validate the results. Thirdly, for the molecular subtypes and molecular signatures established in this study, it is necessary to be confirmed in a larger multi-center prospective study for the immunotherapy efficacy prediction and prognosis assessment.

## Conclusion

This study suggests that ICRGs were associated with TIME formation and prognosis of PAAD patients, which may serve as novel clinical biomarkers and therapeutic targets. Three ICRGs-based subtypes, two molecular signatures, and nomograms were identified for clinical use. Functional annotation, GESA, TIME, immune infiltration analysis, and ICRG-TF networks were preformed to realize underlying mechanisms.

## Declaration

Ethical Approval and Consent to participate: Not applicable, because the Cancer Genome Atlas (TCGA) and ICGC belong to public databases, the patients involved in the database have obtained ethical approval, users can download relevant data for free for research and publish relevant articles, and the present study is based on open-source data, and the Suzhou Hospital of Anhui Medical University does not require research using publicly available data to be submitted for review to their ethics committee, so there are no ethical issues and other conflicts of interest.

## Consent for publication: not applicable

Availability of supporting data: The datasets used to support the findings of this study are available from TCGA (https://cancergenome.nih.gov/) and ICGC (https://dcc.icgc.org/) databases.

## Authors' contributions

Xiao Chen, and Herui Zhang conceived of and designed the study. Xiao Chen performed a literature search, generated the figures and tables, and analyzed the data. Xiao Chen wrote the manuscript and Herui Zhang critically reviewed the manuscript.

## Funding

This study is supported by the National College Students Innovation and Entrepreneurship Training Program (n° 202110343028).

## Conflicts of interest

The authors declare no conflicts of interest.

## Appendix Supplementary materials

Image, application 1
