# Supplementary material for: Comprehensive exploration of immune checkpoint-related genes in the prognosis and tumor immune microenvironment of pancreatic adenocarcinoma
Source: Clinics (Sao Paulo). 2024 Aug 28;79:100481. doi: 10.1016/j.clinsp.2024.100481 (PMC11399560; doi:10.1016/j.clinsp.2024.100481)
Supplement: Supplementary file 1 [file mmc1.doc]

**CLINICS-D-24-00434_Supplementary Material**

**Supplementary Table 1** Two hundred and eighty-three immune checkpoint-related genes.

| **ICRGs** |
| --- |
| AAM |
| ACS |
| ACUN |
| ACYG |
| AJU |
| AKT1 |
| AKT2 |
| AKT3 |
| ALK |
| AMEX |
| AMJ |
| AML |
| AOCE |
| APLA |
| ASN |
| BACU |
| BATF |
| BATF2 |
| BATF3 |
| BBUB |
| ZAP70 |
| PPP2R1B |
| SLAL |
| RAC1 |
| RTP |
| TLR4 |
| PPP2R5A |
| PPS |
| RRO |
| BIU |
| BOM |
| BTA |
| BTLA |
| CCAE |
| CCAN |
| CCAR |
| CCW |
| CD247 |
| CD274 |
| CD28 |
| CD3D |
| CD3E |
| CD3G |
| CD4 |
| CD80 |
| CD86 |
| CDC42 |
| CDK |
| CFA |
| TRAV19 |
| VAV1 |
| SRC |
| RAF1 |
| SALP |
| TLR9 |
| PPP2R5B |
| PRET |
| TRBC1 |
| CFR |
| CGE |
| CHUK |
| CHX |
| CJC |
| CJO |
| CLV |
| CMK |
| CMY |
| CPIC |
| CSAB |
| CSK |
| CSNK2A1 |
| CSNK2A2 |
| CSNK2A3 |
| CSNK2B |
| CTLA4 |
| CVG |
| DLE |
| DRE |
| YES1 |
| UMR |
| SRX |
| RASGRP1 |
| SANH |
| TMU |
| PPP2R5C |
| PRKCQ |
| DRO |
| EAI |
| ECB |
| EEE |
| EGF |
| EGFR |
| EGZ |
| ELK |
| ELS |
| EML4 |
| EPZ |
| ETL |
| FAB |
| FCA |
| FCH |
| FOS |
| FPG |
| KIAA1429 |
| FYN |
| GFR |
| GGA |
| VVP |
| UAH |
| SSC |
| RAY |
| SASA |
| TNFRSF14 |
| PPP2R5D |
| PRR5 |
| GGO |
| GJA |
| GRAP2 |
| GRB2 |
| HAI |
| HGL |
| HIF1A |
| HLA-DPA1 |
| HLA-DPB1 |
| HLA-DQA1 |
| HLA-DQA2 |
| HLA-DQB1 |
| HLA-DQB2 |
| HLA-DRA |
| HLA-DRB1 |
| HLA-DRB3 |
| HLA-DRB4 |
| HLA-DRB5 |
| HRAS |
| HSA |
| XCO |
| TUP |
| STAT1 |
| RBB |
| SBQ |
| TRAC |
| PPP2R5E |
| PSS |
| ICOS |
| ICOSLG |
| IFNG |
| IFNGR1 |
| IFNGR2 |
| IKBKB |
| IKBKG |
| IPU |
| JAK1 |
| JAK2 |
| JUN |
| KMR |
| KRAS |
| LAT |
| LAV |
| LCF |
| LCK |
| LCM |
| LSR |
| LVE |
| XLA |
| TSR |
| STAT3 |
| RELA |
| SCAN |
| TRAF6 |
| PPP3CA |
| PTEN |
| LYN |
| MALB |
| MAP2K1 |
| MAP2K2 |
| MAP2K3 |
| MAP2K6 |
| MAP3K14 |
| MAP3K3 |
| MAP3K8 |
| MAPK1 |
| MAPK11 |
| MAPK12 |
| MAPK13 |
| MAPK14 |
| MAPK3 |
| MAPKAP1 |
| MCAL |
| MCC |
| MCF |
| MDO |
| XMA |
| TRIB3 |
| TGU |
| RICTOR |
| SDU |
| TLR2 |
| PPP3CB |
| PTG |
| MGP |
| MJV |
| MLST8 |
| MMU |
| MNA |
| MPAH |
| MTOR |
| MUN |
| MYB |
| MYD |
| MYD88 |
| MZE |
| NFATC1 |
| NFATC2 |
| NFATC3 |
| NFKB1 |
| NFKBIA |
| NFKBIB |
| NFKBIE |
| NGI |
| XTR |
| TRBV7-9 |
| THEM4 |
| RNO |
| SFM |
| PVT |
| PPP3CC |
| PTPN11 |
| NLE |
| NMEL |
| NNI |
| NRAS |
| OAA |
| OAS |
| OCU |
| OLA |
| ONL |
| OOR |
| ORO |
| OTW |
| PADL |
| PAK1 |
| PAK2 |
| PAK3 |
| PALE |
| PBI |
| PCAD |
| PCW |
| PDCD1 |
| TRBV12-3 |
| TICAM1 |
| RPS6KB1 |
| SGH |
| TRAV8-4 |
| PPP3R1 |
| PTPN6 |
| PDCD1LG2 |
| PDPK1 |
| PHI |
| PHYP |
| PIK3CA |
| PIK3CB |
| PIK3CD |
| PIK3R1 |
| PIK3R2 |
| PIK3R3 |
| PKI |
| PLCG1 |
| PMAJ |
| PMUA |
| PMUR |
| PON |
| POV |
| PPAD |
| PPP2CA |
| PPP2CB |
| PPP2R1A |
| TRAV29DV5 |
| TICAM2 |
| RPS6KB2 |
| SHR |
| TIRAP |
| PPP3R2 |
| PTR |

**Supplementary Table 2** Clinicopathologic factors of PAAD patients.

| **Factors** | **n = 177** |
| --- | --- |
| **Age, year** | 64.68 ± 0.81 |
| **Gender** |  |
| Male | 97 (54.8%) |
| Female | 80 (45.2%) |
| **Grade** |  |
| I‒II | 125 (70.6%) |
| III‒IV | 50 (28.3%) |
| Unknow | 2 (1.1%) |
| **T stage** |  |
| I‒II | 31 (17.5%) |
| III‒IV | 144 (81.4%) |
| Unknow | 2 (1.1%) |
| **N stage** |  |
| N0 | 50 (28.3%) |
| N1 | 122 (68.9%) |
| Unknow | 5 (2.8%) |
| **AJCC Stage** |  |
| I‒II | 166 (93.8%) |
| III‒IV | 9 (5.1%) |
| Unknow | 2 (1.1%) |
| **Survival status** |  |
| Dead | 92 (52.0%) |
| Alive | 85 (48.0%) |
| **Median OS time, year** | 1.663 (95% CI 1.418‒1.908) |
| **Median PFS time, year** | 1.332 (95% CI 1.111‒1.552) |

**Supplementary Table 3** One hundred and twenty-four DEICRGs between PAAD and normal pancreatic tissues.

| **Gene** | **conMean** | **treatMean** | **logFC** | **p-value** | **FDR** |
| --- | --- | --- | --- | --- | --- |
| TICAM2 | 1,251298 | 0,065565 | -4,25436 | 1,71E-57 | 2,77E-56 |
| LAT | 2,207021 | 0,200323 | -3,4617 | 1,71E-57 | 2,77E-56 |
| PIK3R2 | 3,027098 | 0,289598 | -3,38581 | 1,71E-57 | 2,77E-56 |
| EGF | 4,063207 | 0,495967 | -3,0343 | 2,18E-54 | 1,22E-53 |
| PAK3 | 3,136477 | 0,958859 | -1,70975 | 2,74E-48 | 6,67E-48 |
| ICOSLG | 0,546256 | 0,195093 | -1,48542 | 1,21E-38 | 2,08E-38 |
| CSNK2A3 | 0,304232 | 0,122352 | -1,31413 | 3,98E-27 | 5,64E-27 |
| MAP2K6 | 2,061423 | 1,047526 | -0,97665 | 4,52E-44 | 9,10E-44 |
| PDPK1 | 3,216037 | 1,860166 | -0,78985 | 7,84E-57 | 7,78E-56 |
| TRBC1 | 0,726157 | 0,428353 | -0,76148 | 2,45E-15 | 3,13E-15 |
| AKT2 | 5,125745 | 3,063444 | -0,74261 | 5,25E-57 | 5,64E-56 |
| MAPK12 | 1,475538 | 0,943125 | -0,64572 | 9,26E-31 | 1,37E-30 |
| THEM4 | 3,279365 | 2,162394 | -0,60079 | 2,58E-54 | 1,39E-53 |
| RPS6KB2 | 4,352316 | 2,938862 | -0,56653 | 2,00E-57 | 2,77E-56 |
| CSNK2B | 5,969122 | 4,149704 | -0,52451 | 1,71E-57 | 2,77E-56 |
| KIAA1429 | 3,537805 | 2,606467 | -0,44076 | 6,46E-53 | 2,61E-52 |
| PRR5 | 2,634384 | 1,948824 | -0,43486 | 7,94E-33 | 1,20E-32 |
| AKT1 | 5,116787 | 3,821153 | -0,42123 | 1,71E-57 | 2,77E-56 |
| PPP2R1B | 3,444552 | 2,618698 | -0,39547 | 1,66E-45 | 3,45E-45 |
| RAF1 | 4,773744 | 3,656749 | -0,38456 | 2,00E-57 | 2,77E-56 |
| MLST8 | 4,352498 | 3,352225 | -0,37672 | 1,54E-53 | 7,35E-53 |
| RPS6KB1 | 2,853431 | 2,243022 | -0,34725 | 5,02E-46 | 1,08E-45 |
| CSNK2A2 | 4,291024 | 3,419147 | -0,32769 | 6,13E-47 | 1,39E-46 |
| PLCG1 | 3,855124 | 3,078941 | -0,32434 | 4,15E-41 | 7,44E-41 |
| MTOR | 3,026772 | 2,442274 | -0,30956 | 2,45E-42 | 4,65E-42 |
| PRKCQ | 1,22864 | 0,999671 | -0,29754 | 1,74E-08 | 2,03E-08 |
| MAPKAP1 | 4,114642 | 3,518028 | -0,226 | 2,34E-43 | 4,51E-43 |
| TNFRSF14 | 4,412757 | 3,912241 | -0,17368 | 3,01E-12 | 3,62E-12 |
| MAPK13 | 4,22983 | 3,751194 | -0,17325 | 2,97E-27 | 4,25E-27 |
| PTEN | 3,95079 | 3,512302 | -0,16972 | 1,12E-24 | 1,56E-24 |
| PIK3CB | 3,09828 | 2,778344 | -0,15724 | 1,17E-14 | 1,47E-14 |
| MAP3K14 | 2,499858 | 2,324019 | -0,10522 | 2,00E-05 | 2,20E-05 |
| TIRAP | 1,775656 | 1,658776 | -0,09823 | 8,66E-07 | 9,80E-07 |
| PPP2R1A | 5,263889 | 5,370171 | 0,028839 | 0,000262 | 0,000282 |
| JUN | 5,748463 | 5,952125 | 0,050228 | 0,003296 | 0,003485 |
| EML4 | 3,555248 | 3,698734 | 0,057081 | 8,19E-06 | 9,19E-06 |
| HRAS | 3,997585 | 4,164818 | 0,059125 | 0,000602 | 0,000642 |
| MAPK14 | 3,127186 | 3,276018 | 0,067078 | 4,83E-10 | 5,67E-10 |
| PPP3CB | 3,356801 | 3,52104 | 0,068915 | 1,53E-05 | 1,70E-05 |
| NFKBIA | 5,554713 | 5,878124 | 0,081644 | 2,74E-08 | 3,13E-08 |
| TRIB3 | 2,59367 | 2,746318 | 0,082504 | 0,045185 | 0,047007 |
| FYN | 2,755365 | 2,949491 | 0,098223 | 4,50E-05 | 4,92E-05 |
| PTPN6 | 3,531546 | 3,811918 | 0,110217 | 2,30E-08 | 2,65E-08 |
| PPP2R5B | 3,059828 | 3,367213 | 0,138105 | 8,97E-13 | 1,09E-12 |
| PPP2R5A | 3,861679 | 4,295872 | 0,153723 | 4,40E-21 | 5,86E-21 |
| CSNK2A1 | 3,598213 | 4,006181 | 0,154947 | 2,76E-29 | 4,00E-29 |
| MAPK1 | 3,202895 | 3,56656 | 0,155157 | 1,80E-19 | 2,32E-19 |
| PAK1 | 3,430865 | 3,907553 | 0,187693 | 6,90E-22 | 9,27E-22 |
| MGP | 6,280117 | 7,338431 | 0,22468 | 8,15E-13 | 1,00E-12 |
| MAP3K8 | 1,895874 | 2,24275 | 0,242406 | 1,98E-11 | 2,36E-11 |
| PPP2CA | 3,856895 | 4,580329 | 0,248011 | 1,34E-48 | 3,32E-48 |
| FOS | 5,906715 | 7,058603 | 0,257027 | 3,54E-11 | 4,19E-11 |
| NFATC1 | 1,450593 | 1,7357 | 0,258875 | 9,50E-05 | 0,000103 |
| PPP2R5D | 3,22325 | 3,896053 | 0,273497 | 8,50E-54 | 4,39E-53 |
| CDC42 | 4,82877 | 5,955416 | 0,302547 | 2,15E-55 | 1,32E-54 |
| TRAF6 | 1,551485 | 1,924058 | 0,310503 | 1,75E-32 | 2,63E-32 |
| MAP2K1 | 3,289609 | 4,089041 | 0,313847 | 5,06E-53 | 2,23E-52 |
| JAK1 | 4,10833 | 5,138774 | 0,322872 | 7,37E-49 | 2,07E-48 |
| PIK3CA | 1,593789 | 2,003581 | 0,330121 | 1,86E-23 | 2,53E-23 |
| MAPK3 | 3,909075 | 4,983116 | 0,350221 | 1,85E-52 | 6,80E-52 |
| LSR | 5,315252 | 6,787881 | 0,352823 | 1,99E-41 | 3,68E-41 |
| MAP2K3 | 3,528293 | 4,527012 | 0,359589 | 1,08E-49 | 3,23E-49 |
| CSK | 3,216791 | 4,152589 | 0,368389 | 7,99E-49 | 2,19E-48 |
| RAC1 | 5,442689 | 7,085451 | 0,38054 | 1,83E-57 | 2,77E-56 |
| PIK3R1 | 2,123986 | 2,783445 | 0,390098 | 4,02E-24 | 5,51E-24 |
| NFKB1 | 2,830256 | 3,711639 | 0,391124 | 9,47E-49 | 2,49E-48 |
| PPP3R1 | 3,054898 | 4,065631 | 0,412355 | 1,15E-56 | 1,06E-55 |
| PIK3R3 | 1,467251 | 1,954718 | 0,413844 | 2,28E-20 | 2,97E-20 |
| ZAP70 | 0,837587 | 1,128204 | 0,429716 | 0,010014 | 0,010502 |
| NFKBIB | 2,8826 | 3,910957 | 0,440151 | 5,19E-53 | 2,23E-52 |
| IFNGR1 | 3,931141 | 5,346077 | 0,443533 | 5,11E-53 | 2,23E-52 |
| YES1 | 2,762592 | 3,763862 | 0,446191 | 6,43E-47 | 1,43E-46 |
| PTPN11 | 2,8198 | 3,92301 | 0,476368 | 1,62E-50 | 5,08E-50 |
| GRB2 | 3,61522 | 5,06052 | 0,485202 | 2,04E-57 | 2,77E-56 |
| PPP2R5E | 1,891632 | 2,662053 | 0,492908 | 6,04E-53 | 2,51E-52 |
| KRAS | 2,186388 | 3,170399 | 0,536115 | 1,20E-48 | 3,09E-48 |
| MAPK11 | 1,750105 | 2,604989 | 0,573836 | 4,88E-34 | 7,59E-34 |
| AKT3 | 1,624232 | 2,46709 | 0,603053 | 9,12E-35 | 1,45E-34 |
| IFNGR2 | 3,643025 | 5,566737 | 0,611695 | 1,09E-55 | 7,69E-55 |
| MYD88 | 2,860733 | 4,371653 | 0,611794 | 1,04E-52 | 4,08E-52 |
| STAT1 | 3,219212 | 5,051978 | 0,650141 | 4,91E-52 | 1,71E-51 |
| HLA-DPA1 | 3,410204 | 5,435134 | 0,672458 | 3,47E-40 | 6,13E-40 |
| LYN | 2,154961 | 3,43685 | 0,673425 | 7,08E-49 | 2,03E-48 |
| JAK2 | 1,339385 | 2,216786 | 0,726899 | 1,60E-43 | 3,13E-43 |
| PAK2 | 2,493949 | 4,226593 | 0,761063 | 3,27E-57 | 3,84E-56 |
| PIK3CD | 1,233391 | 2,101095 | 0,768511 | 1,34E-34 | 2,11E-34 |
| NRAS | 2,133768 | 3,771724 | 0,821821 | 1,42E-56 | 1,22E-55 |
| HLA-DPB1 | 3,73172 | 6,596592 | 0,82188 | 2,64E-50 | 8,12E-50 |
| TLR2 | 1,444596 | 2,585824 | 0,839958 | 1,12E-38 | 1,95E-38 |
| TICAM1 | 2,274035 | 4,173668 | 0,876062 | 2,15E-57 | 2,77E-56 |
| NFKBIE | 1,939268 | 3,687141 | 0,92699 | 1,57E-52 | 5,97E-52 |
| MCC | 0,855235 | 1,680032 | 0,974096 | 1,63E-45 | 3,45E-45 |
| HLA-DRA | 4,577902 | 9,820511 | 1,101111 | 1,30E-55 | 8,38E-55 |
| CD4 | 1,732519 | 3,752646 | 1,115037 | 1,74E-51 | 5,90E-51 |
| CD80 | 0,213851 | 0,474094 | 1,148563 | 8,03E-15 | 1,02E-14 |
| NFATC2 | 0,982975 | 2,179753 | 1,148938 | 1,36E-53 | 6,73E-53 |
| RASGRP1 | 0,618522 | 1,392517 | 1,170797 | 1,68E-29 | 2,46E-29 |
| HLA-DQB1 | 2,109049 | 4,776555 | 1,179378 | 9,12E-44 | 1,81E-43 |
| HLA-DRB1 | 3,679296 | 8,897257 | 1,273931 | 4,23E-56 | 3,41E-55 |
| TLR4 | 0,724178 | 1,989338 | 1,457872 | 1,27E-48 | 3,20E-48 |
| CD247 | 0,451181 | 1,260221 | 1,4819 | 6,08E-38 | 1,02E-37 |
| HLA-DQA1 | 1,445438 | 4,047287 | 1,485449 | 6,10E-48 | 1,43E-47 |
| CD3E | 0,90093 | 2,573413 | 1,514196 | 1,61E-44 | 3,30E-44 |
| TRAC | 1,061923 | 3,281798 | 1,627807 | 9,17E-49 | 2,46E-48 |
| VAV1 | 0,656034 | 2,296828 | 1,807801 | 3,48E-48 | 8,32E-48 |
| HLA-DRB5 | 1,785648 | 6,786649 | 1,926252 | 1,12E-54 | 6,56E-54 |
| PDCD1 | 0,242706 | 0,95551 | 1,977059 | 2,72E-38 | 4,63E-38 |
| BTLA | 0,105264 | 0,415453 | 1,980676 | 2,70E-14 | 3,34E-14 |
| CD3G | 0,205192 | 0,848004 | 2,047098 | 8,65E-36 | 1,39E-35 |
| CD3D | 0,636624 | 2,724319 | 2,097382 | 1,13E-50 | 3,63E-50 |
| TRBV12-3 | 0,053605 | 0,234941 | 2,131865 | 8,96E-21 | 1,18E-20 |
| LCK | 0,552783 | 2,479649 | 2,165351 | 1,95E-51 | 6,46E-51 |
| MYB | 0,121031 | 0,717768 | 2,568138 | 3,76E-49 | 1,10E-48 |
| PDCD1LG2 | 0,169025 | 1,025899 | 2,601583 | 3,82E-52 | 1,37E-51 |
| IFNG | 0,020823 | 0,13411 | 2,687162 | 6,19E-27 | 8,68E-27 |
| BATF | 0,554949 | 3,80274 | 2,776613 | 5,50E-56 | 4,17E-55 |
| TRBV7-9 | 0,141193 | 1,034625 | 2,873366 | 2,33E-41 | 4,24E-41 |
| CD28 | 0,091576 | 0,711586 | 2,957991 | 1,16E-46 | 2,54E-46 |
| CTLA4 | 0,129277 | 1,015357 | 2,973444 | 3,86E-47 | 8,90E-47 |
| TRAV19 | 0,058713 | 0,496705 | 3,08063 | 9,89E-38 | 1,64E-37 |
| ICOS | 0,060886 | 0,571518 | 3,230625 | 8,35E-42 | 1,56E-41 |
| TRAV29DV5 | 0,036228 | 0,370244 | 3,353314 | 6,69E-34 | 1,03E-33 |
| TRAV8-4 | 0,034512 | 0,389185 | 3,495291 | 1,23E-37 | 2,01E-37 |
| HLA-DQB2 | 0,268968 | 3,16997 | 3,558963 | 1,13E-55 | 7,69E-55 |

**Supplementary Table 4** Thirty-five overall survival related DEICRGs.

| **id** | **HR** | **HR.95L** | **HR.95H** | **p-value** |
| --- | --- | --- | --- | --- |
| STAT1 | 1,518638 | 1,208793 | 1,907903 | 0,000332 |
| EML4 | 1,570133 | 1,024637 | 2,40604 | 0,038291 |
| KIAA1429 | 2,180607 | 1,24926 | 3,806293 | 0,006088 |
| FYN | 0,59097 | 0,397366 | 0,878902 | 0,009394 |
| IFNG | 2,210563 | 1,083355 | 4,510607 | 0,029258 |
| CDC42 | 1,810604 | 1,071505 | 3,059518 | 0,026555 |
| PPP3R1 | 3,264412 | 1,353817 | 7,871367 | 0,008425 |
| CSNK2A3 | 16,58879 | 2,345756 | 117,3131 | 0,004889 |
| PIK3CA | 2,459702 | 1,507802 | 4,012553 | 0,000313 |
| TRAF6 | 2,671923 | 1,435928 | 4,97182 | 0,001923 |
| THEM4 | 0,388499 | 0,19672 | 0,767243 | 0,006468 |
| CD80 | 2,104346 | 1,13588 | 3,898536 | 0,018032 |
| NRAS | 2,61575 | 1,656017 | 4,131689 | 3,75E-05 |
| PTPN6 | 0,617356 | 0,401789 | 0,948579 | 0,027746 |
| PAK2 | 2,902433 | 1,699975 | 4,955438 | 9,46E-05 |
| LYN | 1,543377 | 1,105695 | 2,154312 | 0,010758 |
| MAPK12 | 0,624707 | 0,417334 | 0,935122 | 0,022262 |
| YES1 | 1,834938 | 1,209527 | 2,783732 | 0,00431 |
| PDCD1LG2 | 1,39463 | 1,007519 | 1,930477 | 0,04495 |
| MAP3K14 | 0,527493 | 0,323989 | 0,858821 | 0,010113 |
| MAPK1 | 1,939252 | 1,122855 | 3,349228 | 0,017521 |
| KRAS | 2,19814 | 1,42837 | 3,38275 | 0,000342 |
| MAPK11 | 0,662649 | 0,498239 | 0,881311 | 0,004679 |
| PAK1 | 1,586622 | 1,116498 | 2,254699 | 0,010036 |
| PAK3 | 0,685924 | 0,525637 | 0,895089 | 0,005501 |
| HRAS | 0,690312 | 0,490295 | 0,971926 | 0,033746 |
| GRB2 | 0,466754 | 0,251685 | 0,865604 | 0,015607 |
| PPP3CB | 0,463704 | 0,261122 | 0,823454 | 0,008718 |
| PPP2R5E | 3,598522 | 1,608239 | 8,051889 | 0,001832 |
| MYD88 | 1,849808 | 1,24464 | 2,749222 | 0,002346 |
| PPP2R1A | 0,409804 | 0,207309 | 0,810093 | 0,010297 |
| PIK3CB | 3,266365 | 1,905184 | 5,600057 | 1,68E-05 |
| EGF | 1,322272 | 1,019076 | 1,715675 | 0,035539 |
| LAT | 0,123114 | 0,016375 | 0,925643 | 0,041848 |
| PLCG1 | 0,421006 | 0,238507 | 0,74315 | 0,002846 |

**Supplementary Table 5** Thirty-eight progression free survival related DEICRGs.

| **id** | **HR** | **HR.95L** | **HR.95H** | **p-value** |
| --- | --- | --- | --- | --- |
| STAT1 | 1,422593 | 1,139907 | 1,775382 | 0,001818 |
| PIK3R3 | 0,684096 | 0,471504 | 0,992541 | 0,045566 |
| EML4 | 1,6324 | 1,105572 | 2,410274 | 0,013711 |
| NFKBIE | 1,36657 | 1,033479 | 1,807016 | 0,028452 |
| KIAA1429 | 2,060963 | 1,226205 | 3,463996 | 0,006339 |
| FYN | 0,638254 | 0,436455 | 0,933356 | 0,020578 |
| RAF1 | 2,834201 | 1,325283 | 6,061117 | 0,007229 |
| PPP3R1 | 2,642601 | 1,181617 | 5,909986 | 0,017965 |
| CSNK2A3 | 17,28624 | 2,865831 | 104,2678 | 0,001882 |
| CSNK2A1 | 2,105649 | 1,213352 | 3,654139 | 0,008107 |
| PIK3CA | 2,393611 | 1,499342 | 3,821257 | 0,000255 |
| TRAF6 | 2,16898 | 1,206174 | 3,900331 | 0,009708 |
| MAP2K6 | 0,569092 | 0,360491 | 0,8984 | 0,015525 |
| THEM4 | 0,424551 | 0,218101 | 0,826422 | 0,011703 |
| BATF | 1,207376 | 1,019025 | 1,430541 | 0,029424 |
| CD80 | 2,093836 | 1,172859 | 3,738001 | 0,012448 |
| NFKB1 | 1,603353 | 1,016052 | 2,530126 | 0,042521 |
| NRAS | 2,621385 | 1,696572 | 4,050318 | 1,42E-05 |
| PTPN6 | 0,668822 | 0,454321 | 0,984598 | 0,041487 |
| PAK2 | 2,940209 | 1,771974 | 4,878644 | 2,99E-05 |
| LYN | 1,464534 | 1,075285 | 1,994688 | 0,015502 |
| MAPK12 | 0,54811 | 0,369094 | 0,813952 | 0,00288 |
| CSNK2A2 | 2,176532 | 1,108678 | 4,27292 | 0,023839 |
| YES1 | 1,699963 | 1,154166 | 2,503863 | 0,007238 |
| MAP2K3 | 1,577033 | 1,071928 | 2,32015 | 0,020746 |
| MAPK1 | 1,749735 | 1,028508 | 2,97671 | 0,039051 |
| KRAS | 1,972464 | 1,308994 | 2,972216 | 0,001166 |
| MAPK11 | 0,637592 | 0,48682 | 0,835059 | 0,001078 |
| PAK3 | 0,670783 | 0,523565 | 0,859396 | 0,001586 |
| PPP2R5B | 0,512194 | 0,330074 | 0,794799 | 0,002841 |
| PPP3CB | 0,48263 | 0,287401 | 0,810479 | 0,005879 |
| PPP2R5E | 2,344234 | 1,093689 | 5,024674 | 0,02851 |
| MYD88 | 1,846574 | 1,296962 | 2,629095 | 0,000668 |
| PIK3CB | 2,687638 | 1,626426 | 4,441269 | 0,000114 |
| EGF | 1,399995 | 1,102898 | 1,777123 | 0,005697 |
| MTOR | 2,07098 | 1,120668 | 3,827144 | 0,020149 |
| LAT | 0,154582 | 0,0251 | 0,952033 | 0,044119 |
| PLCG1 | 0,482804 | 0,279717 | 0,833341 | 0,008933 |

**Supplementary Table 6** Nine hundred and twenty DEGs between high- and low-risk groups in the OS-related signature.

| **Gene** | **conMean** | **treatMean** | **logFC** | **p-value** | **fdr** |
| --- | --- | --- | --- | --- | --- |
| HAS1 | 3,287533 | 0,83578 | -1,97581 | 0,002598 | 0,008901 |
| BLK | 2,524824 | 0,380662 | -2,7296 | 0,000289 | 0,001592 |
| OR2L13 | 0,053829 | 0,011122 | -2,27495 | 0,018954 | 0,042483 |
| SCN4A | 0,108448 | 0,038507 | -1,49383 | 2,44E-07 | 6,87E-06 |
| TFAP2C | 0,812414 | 1,78762 | 1,137753 | 0,003363 | 0,010892 |
| S100B | 11,62437 | 4,816905 | -1,27097 | 6,73E-06 | 8,68E-05 |
| PNMA3 | 0,956223 | 0,194096 | -2,30058 | 4,98E-07 | 1,19E-05 |
| PABPN1L | 0,033518 | 0,011188 | -1,58302 | 4,89E-05 | 0,000405 |
| BPIFB4 | 0,298469 | 0,010438 | -4,83766 | 0,00056 | 0,00266 |
| GJD4 | 0,070448 | 0,024664 | -1,51414 | 0,003325 | 0,010815 |
| TENM1 | 0,20963 | 0,094546 | -1,14876 | 0,012482 | 0,030457 |
| TMEM59L | 5,498312 | 1,33678 | -2,04023 | 0,000127 | 0,00084 |
| CBLN4 | 0,957703 | 0,460203 | -1,05731 | 0,002159 | 0,007694 |
| TRARG1 | 0,945184 | 0,166319 | -2,50665 | 6,84E-05 | 0,000525 |
| SERPINB5 | 9,019067 | 23,69589 | 1,393587 | 1,01E-10 | 3,31E-08 |
| NGFR | 11,68048 | 4,503701 | -1,37492 | 1,44E-06 | 2,67E-05 |
| PPM1E | 0,410305 | 0,198383 | -1,04841 | 0,007232 | 0,01989 |
| VTN | 33,63105 | 4,902988 | -2,77806 | 0,019447 | 0,043264 |
| CLEC4F | 0,491781 | 0,22129 | -1,15207 | 3,07E-06 | 4,67E-05 |
| KRT27 | 0,077212 | 0,037849 | -1,02857 | 0,000537 | 0,002568 |
| MUC16 | 1,024429 | 2,724538 | 1,411192 | 3,46E-05 | 0,000306 |
| ADCY1 | 1,644291 | 0,416929 | -1,97959 | 0,007425 | 0,020304 |
| CD3E | 10,00125 | 4,772921 | -1,06724 | 0,001976 | 0,007175 |
| CACNA1A | 2,979632 | 0,524449 | -2,50626 | 0,001169 | 0,00473 |
| KCNH3 | 1,318926 | 0,328111 | -2,00711 | 4,07E-06 | 5,80E-05 |
| JPH4 | 0,833485 | 0,276862 | -1,58999 | 1,04E-05 | 0,000122 |
| AFF3 | 0,700404 | 0,268181 | -1,38498 | 0,000714 | 0,003213 |
| RIIAD1 | 0,714436 | 0,229243 | -1,63993 | 0,016073 | 0,03716 |
| TDRD10 | 0,482193 | 0,227522 | -1,0836 | 3,29E-09 | 3,25E-07 |
| CNTNAP5 | 0,092397 | 0,038309 | -1,27019 | 0,022032 | 0,047749 |
| CPE | 197,2572 | 55,53408 | -1,82863 | 0,002428 | 0,008452 |
| APOF | 0,093791 | 0,022848 | -2,03736 | 0,0085 | 0,022575 |
| LGALS12 | 0,543335 | 0,200511 | -1,43816 | 0,001524 | 0,005821 |
| ALLC | 0,068134 | 0,031669 | -1,10533 | 0,000325 | 0,001742 |
| CD19 | 4,33324 | 0,641088 | -2,75685 | 0,002202 | 0,007812 |
| CYP46A1 | 0,367066 | 0,083788 | -2,13122 | 1,01E-06 | 2,04E-05 |
| INSM2 | 0,055155 | 0,011033 | -2,3216 | 0,000179 | 0,001098 |
| COL26A1 | 0,682574 | 0,242168 | -1,49498 | 0,000892 | 0,003829 |
| DIRAS3 | 8,675934 | 2,634866 | -1,71929 | 0,000306 | 0,001663 |
| VWA5B2 | 5,698041 | 0,969987 | -2,55443 | 0,002753 | 0,009294 |
| UGT1A1 | 0,138873 | 0,469506 | 1,757379 | 9,59E-09 | 6,61E-07 |
| CACNB2 | 1,216466 | 0,473191 | -1,3622 | 2,81E-06 | 4,38E-05 |
| CPA5 | 0,21884 | 0,045305 | -2,27213 | 7,90E-05 | 0,000586 |
| DAW1 | 0,096975 | 0,255593 | 1,398159 | 0,006861 | 0,019091 |
| PPP1R1A | 9,508959 | 1,913702 | -2,31292 | 0,000901 | 0,003857 |
| SRRM4 | 0,102341 | 0,023437 | -2,12655 | 0,000347 | 0,001837 |
| CETP | 1,466214 | 0,586927 | -1,32084 | 2,67E-05 | 0,00025 |
| NFASC | 2,442642 | 1,038933 | -1,23334 | 0,00027 | 0,001509 |
| P2RX5 | 2,042703 | 0,361877 | -2,49691 | 0,000176 | 0,001084 |
| SH3GL3 | 0,182004 | 0,032137 | -2,50165 | 0,002221 | 0,00787 |
| PDXP | 0,462755 | 0,206236 | -1,16596 | 0,000137 | 0,000896 |
| TSPEAR | 0,309192 | 0,104474 | -1,56536 | 7,75E-05 | 0,000579 |
| NLRP4 | 0,064653 | 0,015018 | -2,10598 | 0,003261 | 0,010659 |
| GPX3 | 92,3044 | 24,48279 | -1,91463 | 6,15E-08 | 2,42E-06 |
| C19orf81 | 0,792268 | 0,107154 | -2,88631 | 4,54E-06 | 6,33E-05 |
| LY9 | 0,627658 | 0,270236 | -1,21576 | 0,007169 | 0,019758 |
| SLC4A1 | 0,020492 | 0,008444 | -1,27907 | 0,000694 | 0,003142 |
| ARG1 | 0,631065 | 0,08325 | -2,92227 | 0,000452 | 0,002251 |
| PRKCB | 2,073051 | 0,830557 | -1,3196 | 0,000846 | 0,003673 |
| PGA5 | 0,109449 | 0,025096 | -2,12475 | 0,01146 | 0,02852 |
| ABCC8 | 26,54748 | 6,276722 | -2,08049 | 0,016999 | 0,038824 |
| UPK2 | 0,738693 | 2,710789 | 1,875665 | 0,002861 | 0,009588 |
| UGT1A6 | 0,343045 | 0,719737 | 1,069071 | 5,94E-09 | 4,78E-07 |
| MAPK8IP1 | 8,468924 | 3,287071 | -1,36538 | 3,29E-06 | 4,93E-05 |
| C12orf54 | 0,136898 | 0,063104 | -1,1173 | 0,001918 | 0,007003 |
| FIGNL2 | 0,141559 | 0,068926 | -1,03827 | 0,000419 | 0,002118 |
| RTN1 | 5,787409 | 1,601641 | -1,85337 | 3,08E-05 | 0,00028 |
| LAMA3 | 13,61536 | 27,26314 | 1,001716 | 2,81E-09 | 2,95E-07 |
| TNNT3 | 0,695454 | 0,202694 | -1,77865 | 1,88E-05 | 0,000191 |
| MROH9 | 0,009244 | 0,021229 | 1,199436 | 0,001683 | 0,006317 |
| REELD1 | 0,446475 | 0,136108 | -1,71383 | 3,17E-07 | 8,30E-06 |
| RAPH1 | 0,37988 | 0,775075 | 1,028792 | 4,32E-13 | 7,37E-10 |
| MB | 0,801832 | 1,71585 | 1,097552 | 0,01991 | 0,044076 |
| MGAT4C | 0,06842 | 0,020537 | -1,7362 | 0,003526 | 0,011311 |
| AC243967.1 | 0,047742 | 0,100364 | 1,071925 | 0,002902 | 0,009708 |
| C6orf58 | 32,7748 | 1,162226 | -4,81762 | 0,000246 | 0,001408 |
| RGS11 | 2,793829 | 1,297307 | -1,10672 | 0,000468 | 0,002302 |
| CD247 | 2,542813 | 1,213468 | -1,06729 | 0,00022 | 0,001291 |
| MMD2 | 0,088672 | 0,004308 | -4,36333 | 0,000734 | 0,003295 |
| CTXN2 | 0,165642 | 0,022981 | -2,84954 | 0,004102 | 0,012737 |
| TMOD1 | 5,951441 | 2,359631 | -1,33468 | 0,004745 | 0,014236 |
| TPBGL | 0,616114 | 0,29676 | -1,0539 | 0,00023 | 0,001336 |
| PCDH8 | 0,453796 | 0,032415 | -3,80733 | 0,000931 | 0,003953 |
| ASGR2 | 1,453586 | 0,287453 | -2,33822 | 0,000627 | 0,002909 |
| LIPK | 0,038325 | 0,131826 | 1,782289 | 0,019613 | 0,043564 |
| IL4 | 0,204401 | 0,069889 | -1,54827 | 0,001519 | 0,005811 |
| PACRG | 1,473266 | 0,64584 | -1,18977 | 0,010297 | 0,026224 |
| LIMD2 | 8,633814 | 3,610044 | -1,25798 | 4,47E-07 | 1,10E-05 |
| CNTFR | 1,222342 | 0,597724 | -1,0321 | 8,60E-05 | 0,000626 |
| PADI3 | 0,484544 | 1,640115 | 1,759097 | 0,012568 | 0,030662 |
| ADH1A | 0,769044 | 0,335146 | -1,19828 | 0,00762 | 0,020703 |
| LY6G6C | 0,395049 | 0,805108 | 1,02715 | 0,000108 | 0,000744 |
| RIPPLY2 | 1,462625 | 0,31114 | -2,23292 | 0,003155 | 0,010363 |
| VAT1L | 3,414668 | 1,515732 | -1,17173 | 0,006003 | 0,017153 |
| CRABP2 | 43,72873 | 89,61618 | 1,035178 | 0,000121 | 0,000809 |
| FXYD1 | 0,792387 | 0,369125 | -1,1021 | 5,76E-08 | 2,29E-06 |
| ARMC12 | 0,489644 | 0,203635 | -1,26575 | 7,01E-06 | 8,97E-05 |
| CCL23 | 2,166581 | 0,926533 | -1,22551 | 3,96E-06 | 5,68E-05 |
| TEKT2 | 1,502622 | 0,559823 | -1,42444 | 0,000557 | 0,002644 |
| SLC13A5 | 1,172345 | 2,623213 | 1,161938 | 0,001478 | 0,005681 |
| ACKR1 | 26,38707 | 9,547381 | -1,46665 | 2,05E-08 | 1,13E-06 |
| MAL | 1,807482 | 0,827083 | -1,12788 | 0,008459 | 0,02247 |
| PTGDS | 62,80967 | 27,52697 | -1,19014 | 0,000405 | 0,002068 |
| RGS9 | 3,266917 | 1,134514 | -1,52585 | 0,003088 | 0,010181 |
| HS3ST4 | 0,139904 | 0,016236 | -3,10717 | 0,014013 | 0,033429 |
| BTBD17 | 0,59416 | 0,097527 | -2,60698 | 5,00E-05 | 0,000412 |
| KRT6A | 19,40393 | 54,57053 | 1,491773 | 0,000777 | 0,003437 |
| WNT7A | 2,229467 | 5,321037 | 1,255009 | 4,47E-07 | 1,10E-05 |
| GPR6 | 0,153697 | 0,019022 | -3,01438 | 0,002612 | 0,00894 |
| MUC17 | 3,269561 | 6,669593 | 1,028502 | 7,33E-05 | 0,000552 |
| CD48 | 8,48612 | 3,375901 | -1,32983 | 0,000641 | 0,002957 |
| RUNDC3A | 6,631688 | 0,894555 | -2,89013 | 0,00093 | 0,003951 |
| SULT1B1 | 3,237926 | 7,784752 | 1,265581 | 2,98E-06 | 4,58E-05 |
| SV2A | 4,428956 | 2,063574 | -1,10182 | 0,002549 | 0,008771 |
| LCN10 | 0,167425 | 0,048993 | -1,77285 | 3,90E-09 | 3,60E-07 |
| PCSK1 | 16,02438 | 3,645878 | -2,13593 | 0,006112 | 0,017385 |
| HOXA13 | 0,751756 | 1,823947 | 1,278727 | 0,000356 | 0,001869 |
| NRXN1 | 0,512042 | 0,224802 | -1,18761 | 0,008243 | 0,022016 |
| CCL19 | 77,16518 | 23,89081 | -1,69149 | 2,39E-05 | 0,000231 |
| PTPRN | 31,41012 | 5,162004 | -2,60523 | 0,018259 | 0,041187 |
| ZPBP2 | 0,026546 | 0,006449 | -2,04138 | 0,003521 | 0,011311 |
| LTA | 0,745425 | 0,301404 | -1,30637 | 0,004368 | 0,01334 |
| KHDRBS2 | 0,316234 | 0,058861 | -2,42562 | 1,43E-06 | 2,66E-05 |
| TSPAN33 | 9,230048 | 4,272418 | -1,11129 | 8,66E-08 | 3,15E-06 |
| SPINK2 | 0,529569 | 0,172516 | -1,61809 | 8,08E-05 | 0,000597 |
| LEP | 1,381375 | 0,318669 | -2,11598 | 0,004861 | 0,014522 |
| ALDH3A1 | 2,959929 | 6,219071 | 1,071136 | 0,002944 | 0,009797 |
| SCML4 | 0,527475 | 0,244623 | -1,10854 | 4,62E-06 | 6,40E-05 |
| SLC2A1 | 42,56675 | 86,69033 | 1,026144 | 9,54E-08 | 3,38E-06 |
| CDH10 | 0,752109 | 0,217789 | -1,78801 | 0,019902 | 0,044076 |
| NPFFR2 | 0,136613 | 0,053729 | -1,34632 | 0,014023 | 0,033447 |
| IL24 | 2,829581 | 0,875175 | -1,69294 | 0,000238 | 0,001372 |
| CHRDL2 | 4,434324 | 1,784328 | -1,31333 | 0,000154 | 0,000981 |
| BTLA | 0,82187 | 0,256952 | -1,67741 | 0,000189 | 0,001143 |
| INSYN1 | 0,888693 | 0,409882 | -1,11648 | 3,88E-05 | 0,000336 |
| TPH1 | 0,71108 | 0,156969 | -2,17953 | 1,96E-06 | 3,37E-05 |
| NPM2 | 3,137876 | 1,540975 | -1,02595 | 0,000533 | 0,002552 |
| TAGLN3 | 5,200444 | 0,96775 | -2,42593 | 0,001685 | 0,006317 |
| KIF5A | 1,745642 | 0,637101 | -1,45416 | 0,01991 | 0,044076 |
| CA9 | 30,84299 | 87,58008 | 1,50566 | 3,73E-05 | 0,000327 |
| NPY4R | 0,083535 | 0,209036 | 1,323299 | 0,001436 | 0,005538 |
| TCEAL5 | 3,800338 | 0,680113 | -2,48228 | 1,82E-05 | 0,000187 |
| KCNG1 | 0,756331 | 0,28925 | -1,3867 | 1,60E-05 | 0,000169 |
| CORO1A | 19,51089 | 8,334064 | -1,22719 | 8,60E-05 | 0,000626 |
| KRT222 | 0,374586 | 0,181191 | -1,04779 | 0,000118 | 0,000797 |
| WFIKKN1 | 0,355148 | 0,158972 | -1,15965 | 0,000483 | 0,002362 |
| RIMBP2 | 2,390049 | 0,511011 | -2,22562 | 0,003765 | 0,011919 |
| POU2F2 | 1,500151 | 0,737309 | -1,02477 | 0,00093 | 0,003951 |
| TMC8 | 6,770455 | 3,063702 | -1,14398 | 1,09E-05 | 0,000126 |
| APOH | 21,41915 | 3,120655 | -2,77898 | 0,006981 | 0,019326 |
| NDUFA4L2 | 18,74514 | 7,132941 | -1,39395 | 0,018259 | 0,041187 |
| ANKRD7 | 0,151773 | 0,072782 | -1,06026 | 0,012981 | 0,031496 |
| AHNAK2 | 6,225573 | 13,36154 | 1,101808 | 3,32E-10 | 7,00E-08 |
| CIDEA | 0,912644 | 0,109027 | -3,06537 | 0,000346 | 0,001832 |
| PLA2G2D | 1,986856 | 0,922099 | -1,10749 | 0,000911 | 0,00389 |
| SPDYE4 | 0,026786 | 0,011368 | -1,2365 | 0,000159 | 0,000999 |
| AL096711.2 | 0,047363 | 0,017409 | -1,44396 | 0,01588 | 0,036874 |
| XKR4 | 0,120227 | 0,029953 | -2,00499 | 4,72E-07 | 1,14E-05 |
| CLEC3B | 8,531085 | 4,153601 | -1,03837 | 1,38E-08 | 8,47E-07 |
| CCNA1 | 0,454306 | 0,137692 | -1,72222 | 8,74E-06 | 0,000107 |
| WFDC13 | 0,039869 | 0,081116 | 1,024733 | 0,00427 | 0,01314 |
| HMGA2 | 0,559906 | 1,196578 | 1,095658 | 2,23E-05 | 0,000218 |
| ADCY8 | 0,095015 | 0,010438 | -3,18635 | 0,020294 | 0,044816 |
| ATP2A3 | 42,56662 | 17,37057 | -1,29308 | 1,96E-06 | 3,37E-05 |
| SEZ6 | 2,77562 | 0,189979 | -3,8689 | 0,009023 | 0,023646 |
| ACER1 | 0,070533 | 0,025018 | -1,49533 | 7,79E-07 | 1,66E-05 |
| HOXD11 | 0,007574 | 0,034759 | 2,198181 | 0,012961 | 0,031461 |
| CA10 | 0,395399 | 0,04411 | -3,16412 | 0,013386 | 0,032238 |
| DSCAM | 0,41101 | 0,062509 | -2,71703 | 0,004171 | 0,012886 |
| LGI3 | 1,094615 | 0,239956 | -2,18958 | 0,01243 | 0,030379 |
| SLIT1 | 0,773646 | 0,238612 | -1,69701 | 0,00736 | 0,020159 |
| CD37 | 15,21105 | 5,489869 | -1,47028 | 1,01E-05 | 0,00012 |
| WNT1 | 0,138216 | 0,049937 | -1,46875 | 0,006653 | 0,018634 |
| UCN3 | 26,22482 | 4,364798 | -2,58695 | 0,014469 | 0,034261 |
| NPHS1 | 0,825185 | 0,369861 | -1,15773 | 3,20E-05 | 0,000288 |
| CFAP299 | 0,018364 | 0,008343 | -1,13825 | 0,003826 | 0,012089 |
| PTPRR | 1,267595 | 2,767059 | 1,12626 | 1,35E-05 | 0,000148 |
| STAP1 | 2,094222 | 0,505892 | -2,04951 | 0,007756 | 0,02099 |
| GLRA2 | 0,089053 | 0,014401 | -2,62846 | 0,011898 | 0,029362 |
| E2F7 | 0,357595 | 0,747525 | 1,063798 | 2,61E-09 | 2,79E-07 |
| CD79B | 11,29615 | 2,13864 | -2,40107 | 2,85E-05 | 0,000263 |
| ATOH8 | 1,60262 | 0,653977 | -1,29312 | 1,09E-08 | 7,17E-07 |
| PCBP3 | 1,147944 | 0,536041 | -1,09864 | 7,29E-09 | 5,52E-07 |
| TRDN | 0,022194 | 0,008032 | -1,46636 | 2,92E-05 | 0,000268 |
| HCST | 9,262379 | 4,614315 | -1,00527 | 2,37E-07 | 6,69E-06 |
| SCRT1 | 0,233695 | 0,092503 | -1,33705 | 0,012896 | 0,031326 |
| COX6B2 | 1,564167 | 3,413603 | 1,125901 | 0,001754 | 0,006529 |
| CASP14 | 0,450489 | 3,657874 | 3,021441 | 2,29E-05 | 0,000222 |
| FAIM2 | 2,170931 | 1,070666 | -1,01981 | 0,001122 | 0,004574 |
| MPZ | 1,974478 | 0,819498 | -1,26866 | 5,29E-07 | 1,24E-05 |
| CLCA4 | 0,193915 | 0,510231 | 1,395729 | 0,018245 | 0,041187 |
| RASD1 | 32,5369 | 12,20847 | -1,41419 | 0,000786 | 0,00346 |
| OSR1 | 1,590521 | 0,605192 | -1,39404 | 0,000289 | 0,001592 |
| SPTBN4 | 1,226833 | 0,290974 | -2,07598 | 0,000217 | 0,001279 |
| A1BG | 0,14438 | 0,060647 | -1,25137 | 9,61E-06 | 0,000115 |
| PCP4 | 8,187314 | 1,748809 | -2,22702 | 0,005058 | 0,014985 |
| CACNA1G | 0,171741 | 0,077509 | -1,1478 | 0,008681 | 0,022924 |
| RBFOX3 | 0,300785 | 0,050373 | -2,57801 | 0,006982 | 0,019326 |
| PRR18 | 0,393507 | 0,093928 | -2,06676 | 0,001771 | 0,006577 |
| IGF2BP3 | 0,493981 | 1,260373 | 1,351323 | 4,96E-08 | 2,09E-06 |
| FCRL3 | 1,045093 | 0,326875 | -1,67682 | 0,003001 | 0,009937 |
| GZMK | 4,665588 | 2,229259 | -1,06549 | 0,000462 | 0,002287 |
| ASB11 | 0,012863 | 0,006329 | -1,02305 | 0,005755 | 0,016565 |
| LRRTM2 | 0,208412 | 0,092113 | -1,17797 | 0,004132 | 0,012798 |
| FABP4 | 22,86611 | 4,669135 | -2,29198 | 2,73E-06 | 4,29E-05 |
| APBB1 | 11,06836 | 5,348207 | -1,04931 | 4,98E-07 | 1,19E-05 |
| BRSK2 | 1,036652 | 0,443066 | -1,22634 | 0,001719 | 0,006423 |
| EPGN | 0,024711 | 0,091197 | 1,883806 | 0,018186 | 0,041065 |
| DIRAS1 | 2,589653 | 0,74924 | -1,78926 | 2,26E-05 | 0,00022 |
| CALB1 | 0,470091 | 0,169394 | -1,47255 | 5,18E-05 | 0,000422 |
| CCDC184 | 3,205619 | 1,251812 | -1,35658 | 0,003493 | 0,011233 |
| XDH | 2,884773 | 6,239933 | 1,113073 | 2,54E-06 | 4,10E-05 |
| EFNB3 | 5,380239 | 1,238911 | -2,1186 | 0,002888 | 0,009664 |
| KRT9 | 0,0354 | 0,090862 | 1,359932 | 1,13E-07 | 3,81E-06 |
| RHOH | 2,148658 | 0,851004 | -1,3362 | 0,000303 | 0,001649 |
| NTS | 9,958626 | 0,805292 | -3,62836 | 8,49E-05 | 0,000621 |
| NELL2 | 1,6824 | 0,703376 | -1,25815 | 0,003147 | 0,010344 |
| BCL11A | 1,069479 | 0,45229 | -1,24159 | 2,63E-08 | 1,32E-06 |
| SPX | 0,400055 | 0,173281 | -1,20708 | 0,009733 | 0,0251 |
| KCNJ5 | 2,376297 | 1,090027 | -1,12435 | 0,001349 | 0,005286 |
| TMEM61 | 3,66479 | 1,529653 | -1,26053 | 0,009954 | 0,025527 |
| IQSEC3 | 2,104801 | 0,529837 | -1,99006 | 0,001789 | 0,006629 |
| FXYD6 | 10,22849 | 4,751577 | -1,10611 | 3,12E-05 | 0,000283 |
| ALPG | 1,387197 | 2,922162 | 1,074863 | 0,002743 | 0,009281 |
| ELAVL4 | 0,87869 | 0,355876 | -1,30398 | 0,017411 | 0,039563 |
| OR13A1 | 0,303264 | 0,096297 | -1,65501 | 0,001065 | 0,004405 |
| POU6F2 | 0,605329 | 0,294826 | -1,03786 | 0,014826 | 0,034876 |
| MAFB | 34,61593 | 17,00389 | -1,02557 | 0,005197 | 0,015314 |
| NKX2-2 | 12,61398 | 2,188054 | -2,5273 | 0,001065 | 0,004405 |
| GPR62 | 0,115694 | 0,038617 | -1,58302 | 0,013156 | 0,031788 |
| S1PR4 | 4,612281 | 2,261616 | -1,02813 | 2,17E-05 | 0,000213 |
| ADGRF3 | 0,378579 | 0,135974 | -1,47726 | 0,00018 | 0,001101 |
| C1orf127 | 4,179391 | 1,378798 | -1,59988 | 0,005792 | 0,016651 |
| CCDC151 | 0,757244 | 0,256545 | -1,56155 | 0,000244 | 0,001398 |
| CBLN1 | 2,297192 | 0,839382 | -1,45247 | 2,60E-05 | 0,000245 |
| ADGRB3 | 0,494311 | 0,152987 | -1,69201 | 0,000189 | 0,001143 |
| ARMH1 | 0,953098 | 0,470475 | -1,01851 | 0,00017 | 0,001053 |
| NAP1L5 | 4,612344 | 2,200128 | -1,06791 | 7,90E-05 | 0,000586 |
| AC005154.5 | 0,070419 | 0,034706 | -1,02079 | 9,72E-06 | 0,000116 |
| PSAPL1 | 1,313334 | 2,785006 | 1,084447 | 0,002481 | 0,008588 |
| NIM1K | 0,650563 | 0,224664 | -1,53392 | 0,00022 | 0,001291 |
| BMPER | 0,253932 | 0,119957 | -1,08192 | 0,000438 | 0,002193 |
| FAM181B | 0,270478 | 0,135161 | -1,00083 | 0,002793 | 0,00941 |
| GZMM | 2,905153 | 1,150501 | -1,33635 | 1,70E-06 | 3,01E-05 |
| SORCS1 | 0,491857 | 0,201184 | -1,28973 | 0,000149 | 0,00096 |
| ATCAY | 0,921503 | 0,205338 | -2,16599 | 0,009745 | 0,02512 |
| CEND1 | 1,468699 | 0,301822 | -2,28277 | 0,000313 | 0,001693 |
| SLC16A12 | 2,32469 | 1,007182 | -1,20671 | 0,020864 | 0,045799 |
| C16orf96 | 0,070016 | 0,033895 | -1,04661 | 1,67E-07 | 5,16E-06 |
| IKZF3 | 2,735677 | 1,226738 | -1,15707 | 0,01769 | 0,040084 |
| C14orf180 | 0,242844 | 0,046496 | -2,38486 | 1,81E-05 | 0,000186 |
| SLFN14 | 0,050822 | 0,020166 | -1,33351 | 0,00779 | 0,021075 |
| FAM166A | 0,078232 | 0,035633 | -1,13457 | 0,001255 | 0,005001 |
| KLHL32 | 0,616128 | 0,189716 | -1,69939 | 6,32E-05 | 0,00049 |
| HBA1 | 0,329225 | 0,118814 | -1,47037 | 0,01956 | 0,043501 |
| FAM110D | 2,905142 | 1,373829 | -1,08041 | 7,56E-10 | 1,16E-07 |
| CES5A | 0,043497 | 0,017522 | -1,31178 | 0,01082 | 0,027233 |
| TMEM271 | 0,268013 | 0,07527 | -1,83215 | 0,000215 | 0,00127 |
| KCNN1 | 0,462942 | 0,065817 | -2,81431 | 0,000864 | 0,003735 |
| KLHDC7B | 2,43245 | 7,259125 | 1,577385 | 0,015689 | 0,036482 |
| FCMR | 11,27903 | 3,182715 | -1,82531 | 0,000121 | 0,000809 |
| CGB5 | 4,903454 | 15,21184 | 1,633324 | 0,004802 | 0,014379 |
| HSPA1B | 31,27293 | 63,60625 | 1,024254 | 0,012075 | 0,029704 |
| CD1B | 0,444594 | 0,221628 | -1,00435 | 0,000549 | 0,002619 |
| SHISA7 | 0,471056 | 0,025498 | -4,20745 | 3,35E-05 | 0,0003 |
| AC007192.1 | 0,013581 | 0,006072 | -1,16122 | 0,003878 | 0,012184 |
| GNAZ | 4,612853 | 1,912458 | -1,27023 | 0,00749 | 0,020424 |
| PSCA | 81,04548 | 177,434 | 1,130479 | 1,83E-05 | 0,000187 |
| TSPAN7 | 12,47789 | 4,048775 | -1,62382 | 3,33E-08 | 1,59E-06 |
| PCDH10 | 0,373255 | 0,118439 | -1,65602 | 0,003872 | 0,01217 |
| KLB | 0,362584 | 0,146441 | -1,308 | 0,000769 | 0,00341 |
| ANKLE1 | 0,240544 | 0,119071 | -1,01449 | 7,27E-07 | 1,58E-05 |
| HEMGN | 0,043645 | 0,015429 | -1,50022 | 0,000645 | 0,002975 |
| ANGPTL5 | 0,109951 | 0,033931 | -1,69621 | 7,41E-05 | 0,000557 |
| DSG3 | 1,608078 | 4,728515 | 1,556049 | 0,000379 | 0,001963 |
| CLEC4G | 0,430055 | 0,120581 | -1,83452 | 1,85E-05 | 0,000189 |
| SLC8A2 | 2,788866 | 0,531597 | -2,39127 | 0,019069 | 0,042644 |
| MPPED1 | 0,631744 | 0,006931 | -6,5101 | 0,002691 | 0,00913 |
| FAM171A2 | 1,233073 | 0,596892 | -1,04672 | 0,001405 | 0,005442 |
| ASB2 | 1,30957 | 0,633331 | -1,04806 | 0,000292 | 0,001606 |
| CXCR4 | 76,17827 | 37,59973 | -1,01866 | 0,003363 | 0,010892 |
| FAM107A | 3,827285 | 1,779452 | -1,10489 | 7,85E-08 | 2,95E-06 |
| TNFRSF13B | 0,638262 | 0,140119 | -2,1875 | 0,004008 | 0,012508 |
| FBXO15 | 0,197848 | 0,092219 | -1,10125 | 0,008171 | 0,021883 |
| C5orf49 | 1,206677 | 0,508345 | -1,24716 | 0,00051 | 0,002471 |
| CERS1 | 1,081815 | 0,199698 | -2,43756 | 1,78E-05 | 0,000183 |
| CA6 | 0,098234 | 0,011152 | -3,13886 | 0,00597 | 0,017086 |
| SLURP1 | 0,156259 | 1,014999 | 2,699463 | 0,000157 | 0,000993 |
| POU2AF1 | 3,257806 | 1,225888 | -1,41007 | 0,001099 | 0,004502 |
| UGT1A3 | 0,007875 | 0,019667 | 1,320391 | 0,002554 | 0,008789 |
| SULT1E1 | 1,785255 | 4,51295 | 1,337941 | 0,001771 | 0,006577 |
| KLRB1 | 4,686656 | 2,282991 | -1,03763 | 7,33E-05 | 0,000552 |
| AGTR1 | 1,295649 | 0,571174 | -1,18167 | 1,85E-06 | 3,22E-05 |
| TMEM72 | 0,371896 | 0,176372 | -1,07628 | 0,017132 | 0,039065 |
| SLC22A7 | 0,042949 | 0,015288 | -1,49018 | 0,00078 | 0,003447 |
| CSN1S1 | 0,166204 | 0,069446 | -1,259 | 0,000192 | 0,001156 |
| LCN15 | 0,028647 | 0,010206 | -1,48895 | 0,006314 | 0,017871 |
| TMEM86B | 2,534428 | 1,202806 | -1,07526 | 4,75E-05 | 0,000394 |
| F10 | 13,56454 | 3,667374 | -1,88702 | 0,001405 | 0,005442 |
| TCL1A | 6,483039 | 0,511794 | -3,66304 | 0,000113 | 0,000768 |
| SLC12A5 | 0,834043 | 0,118706 | -2,81274 | 0,000185 | 0,00112 |
| DCX | 0,298144 | 0,066247 | -2,17007 | 0,000344 | 0,001826 |
| RPRM | 0,238066 | 0,089828 | -1,40612 | 0,000356 | 0,001869 |
| ITK | 1,548521 | 0,646673 | -1,25978 | 0,021027 | 0,046025 |
| DSCAML1 | 0,979314 | 0,365555 | -1,42168 | 0,003001 | 0,009937 |
| SLIT3 | 5,429383 | 2,65706 | -1,03096 | 0,000438 | 0,002193 |
| POMC | 1,785852 | 0,596559 | -1,58188 | 4,37E-06 | 6,11E-05 |
| IL13 | 0,035382 | 0,011856 | -1,57739 | 0,000126 | 0,000839 |
| SHISA8 | 0,279842 | 0,047138 | -2,56966 | 0,002116 | 0,007568 |
| SLC6A16 | 0,785745 | 0,324948 | -1,27385 | 4,07E-06 | 5,80E-05 |
| CRMP1 | 8,269674 | 3,345439 | -1,30564 | 0,005689 | 0,016409 |
| MANSC4 | 0,122825 | 0,056628 | -1,11702 | 1,47E-05 | 0,000159 |
| ALPP | 0,611212 | 3,095501 | 2,340429 | 2,72E-05 | 0,000254 |
| SGSM1 | 1,512589 | 0,567922 | -1,41325 | 0,005389 | 0,015737 |
| CAPSL | 1,464253 | 0,301434 | -2,28025 | 0,004207 | 0,012972 |
| IKZF1 | 2,837161 | 1,378302 | -1,04156 | 0,002202 | 0,007812 |
| IGFN1 | 0,354541 | 0,132825 | -1,41642 | 0,000153 | 0,000976 |
| CACNA1I | 0,267538 | 0,096338 | -1,47356 | 9,25E-05 | 0,000661 |
| MAP4K1 | 4,09027 | 1,609012 | -1,34602 | 0,000133 | 0,000872 |
| CD7 | 4,313919 | 2,005272 | -1,1052 | 4,93E-05 | 0,000407 |
| FGF17 | 0,288432 | 0,087994 | -1,71276 | 4,31E-06 | 6,04E-05 |
| TPO | 0,20478 | 0,093906 | -1,12478 | 1,44E-05 | 0,000156 |
| KCNG2 | 0,164624 | 0,055852 | -1,5595 | 4,75E-06 | 6,55E-05 |
| HMGCLL1 | 0,842789 | 0,348312 | -1,27479 | 0,009298 | 0,024172 |
| SELL | 13,747 | 4,96977 | -1,46787 | 0,022892 | 0,049198 |
| DYNC1I1 | 2,143634 | 0,923146 | -1,21543 | 0,019295 | 0,043 |
| PGM5 | 2,927662 | 1,302166 | -1,16884 | 0,000112 | 0,000766 |
| LY6D | 15,82252 | 34,77928 | 1,136249 | 0,000499 | 0,002428 |
| PLPPR1 | 0,524457 | 0,145936 | -1,84549 | 0,004658 | 0,014024 |
| ZFR2 | 0,274826 | 0,064539 | -2,09027 | 0,003854 | 0,012148 |
| OLIG1 | 0,156648 | 0,059241 | -1,40286 | 0,003023 | 0,010008 |
| SHISAL2A | 0,76335 | 0,321181 | -1,24896 | 0,000165 | 0,001028 |
| FEV | 5,410486 | 0,602024 | -3,16786 | 0,005389 | 0,015737 |
| SYN1 | 3,527967 | 1,595172 | -1,14512 | 0,01755 | 0,039826 |
| SLC6A4 | 0,970363 | 0,185408 | -2,38782 | 0,002428 | 0,008452 |
| VEGFD | 0,549859 | 0,198589 | -1,46927 | 4,54E-09 | 3,96E-07 |
| STXBP5L | 0,304802 | 0,08421 | -1,85581 | 0,008681 | 0,022924 |
| CDO1 | 5,350093 | 1,9673 | -1,44335 | 1,33E-05 | 0,000147 |
| CNR2 | 0,671009 | 0,109976 | -2,60914 | 0,000683 | 0,003101 |
| P2RX5-TAX1BP3 | 0,173074 | 0,075982 | -1,18765 | 8,93E-10 | 1,32E-07 |
| TLR10 | 1,149099 | 0,350873 | -1,71148 | 0,000468 | 0,002302 |
| KLHL41 | 1,164134 | 0,330146 | -1,81808 | 0,017273 | 0,039313 |
| TCEAL2 | 8,557726 | 1,493356 | -2,51867 | 0,000781 | 0,003454 |
| NR0B1 | 1,702978 | 0,345082 | -2,30305 | 0,008201 | 0,021955 |
| ARHGAP19-SLIT1 | 0,029752 | 0,005757 | -2,36951 | 0,000333 | 0,001777 |
| NACAD | 3,021114 | 1,122752 | -1,42804 | 0,001322 | 0,005199 |
| ADARB2 | 0,329715 | 0,122348 | -1,43023 | 0,001122 | 0,004574 |
| CASQ1 | 0,339104 | 0,133048 | -1,34978 | 0,000145 | 0,000937 |
| CFD | 25,29601 | 9,843592 | -1,36165 | 3,87E-08 | 1,75E-06 |
| MATK | 1,32862 | 0,650772 | -1,0297 | 1,21E-07 | 4,02E-06 |
| MAP1LC3C | 0,60964 | 0,290838 | -1,06774 | 0,006111 | 0,017385 |
| C8orf86 | 0,018098 | 0,004423 | -2,03283 | 0,000486 | 0,002372 |
| CCER2 | 0,475355 | 0,220375 | -1,10905 | 0,000753 | 0,003356 |
| FAM180B | 0,31119 | 0,097876 | -1,66876 | 1,78E-06 | 3,13E-05 |
| NMUR2 | 1,254926 | 2,54075 | 1,017653 | 0,017118 | 0,039065 |
| OR52N4 | 0,22233 | 0,081171 | -1,45366 | 0,000216 | 0,001276 |
| C4orf50 | 0,036814 | 0,017947 | -1,03655 | 0,005604 | 0,016206 |
| ADAMTS18 | 0,286784 | 0,12863 | -1,15674 | 0,010385 | 0,026389 |
| INSL5 | 0,110217 | 0,018402 | -2,58242 | 0,003105 | 0,010234 |
| FRMPD1 | 0,488726 | 0,110105 | -2,15015 | 0,000272 | 0,001517 |
| FAM222A | 4,392956 | 1,383907 | -1,66645 | 0,000222 | 0,0013 |
| ALK | 0,361152 | 0,050154 | -2,84816 | 0,001825 | 0,006738 |
| ASCL1 | 2,308782 | 0,095524 | -4,59512 | 0,004873 | 0,014547 |
| MALL | 2,842047 | 5,933659 | 1,061992 | 5,05E-08 | 2,12E-06 |
| GATD3A | 0,47307 | 0,205098 | -1,20574 | 0,002611 | 0,00894 |
| DKK1 | 6,544335 | 17,06031 | 1,382326 | 1,04E-06 | 2,09E-05 |
| KCNH2 | 6,387618 | 2,215117 | -1,5279 | 0,010297 | 0,026224 |
| TOGARAM2 | 0,218584 | 0,086875 | -1,33117 | 0,000119 | 0,000802 |
| PHF21B | 0,348766 | 0,079001 | -2,14232 | 9,71E-05 | 0,000688 |
| COL17A1 | 26,18037 | 65,04607 | 1,312976 | 9,54E-08 | 3,38E-06 |
| ASTN2 | 1,420932 | 0,501232 | -1,50329 | 0,000677 | 0,003077 |
| CGB7 | 0,287827 | 0,577671 | 1,005047 | 0,000582 | 0,002736 |
| CELA1 | 0,071091 | 0,035454 | -1,00372 | 5,17E-05 | 0,000422 |
| PPP1R42 | 0,117189 | 0,03474 | -1,75418 | 0,012385 | 0,030281 |
| BSN | 0,523655 | 0,17246 | -1,60236 | 0,016999 | 0,038824 |
| INA | 4,350055 | 1,06907 | -2,02468 | 0,009458 | 0,024497 |
| KCNH6 | 4,371636 | 0,388133 | -3,49355 | 0,000516 | 0,002491 |
| SCN3B | 0,882471 | 0,298671 | -1,56299 | 0,000162 | 0,001015 |
| TACR1 | 0,558234 | 0,208612 | -1,42005 | 2,62E-06 | 4,18E-05 |
| SYP | 9,761766 | 1,655635 | -2,55976 | 0,001087 | 0,004469 |
| SNTN | 0,027115 | 0,072653 | 1,421941 | 0,014557 | 0,03446 |
| GRIN2C | 0,294423 | 0,055982 | -2,39487 | 9,60E-05 | 0,00068 |
| PDZD4 | 4,436976 | 1,351314 | -1,71521 | 4,62E-06 | 6,40E-05 |
| GLTPD2 | 1,247733 | 0,60084 | -1,05426 | 5,38E-05 | 0,000435 |
| SYT3 | 0,371228 | 0,150141 | -1,30599 | 0,010651 | 0,026871 |
| GPR18 | 0,879374 | 0,309512 | -1,50648 | 0,000462 | 0,002287 |
| MTNR1B | 0,025291 | 0,010143 | -1,31807 | 0,006871 | 0,019116 |
| CAMK2N2 | 2,495855 | 0,880373 | -1,50335 | 0,005058 | 0,014985 |
| OGDHL | 2,651008 | 0,747967 | -1,82549 | 0,000557 | 0,002644 |
| LTC4S | 0,060349 | 0,026392 | -1,19324 | 3,01E-07 | 8,10E-06 |
| NRGN | 6,381229 | 3,067318 | -1,05686 | 1,93E-07 | 5,80E-06 |
| LUZP2 | 0,167354 | 0,347654 | 1,054749 | 0,0033 | 0,01074 |
| CUX2 | 0,197786 | 0,028169 | -2,81175 | 0,007892 | 0,02128 |
| SCRG1 | 1,059341 | 0,456815 | -1,21348 | 0,014587 | 0,034467 |
| MROH2A | 0,016477 | 0,054301 | 1,720514 | 0,005038 | 0,014953 |
| LRRC4B | 3,073996 | 0,447273 | -2,78089 | 3,94E-08 | 1,77E-06 |
| DAZL | 0,077953 | 0,02391 | -1,70502 | 0,000251 | 0,001431 |
| UNC13A | 2,234673 | 0,507645 | -2,13817 | 0,001539 | 0,005865 |
| HAVCR1 | 0,395947 | 0,922649 | 1,220474 | 0,000163 | 0,00102 |
| CD22 | 4,559693 | 1,004604 | -2,18231 | 5,52E-05 | 0,000443 |
| TTLL6 | 1,110097 | 0,454208 | -1,28926 | 0,008985 | 0,023549 |
| CD27 | 5,987481 | 2,439179 | -1,29555 | 0,001862 | 0,006845 |
| TESPA1 | 1,159029 | 0,533989 | -1,11804 | 0,00098 | 0,004127 |
| ATP1B2 | 2,543535 | 0,852476 | -1,5771 | 1,62E-06 | 2,93E-05 |
| VWC2 | 0,020671 | 0,008212 | -1,3319 | 0,005109 | 0,015105 |
| FTCD | 3,987429 | 0,221398 | -4,17075 | 0,006167 | 0,017518 |
| SNAP25 | 10,42345 | 2,54531 | -2,03392 | 0,001363 | 0,005324 |
| TRAF3IP3 | 1,733541 | 0,755208 | -1,19878 | 0,000582 | 0,002736 |
| SERTM1 | 1,089254 | 0,154635 | -2,8164 | 0,007228 | 0,01989 |
| PGPEP1L | 0,082608 | 0,015193 | -2,44292 | 5,69E-05 | 0,000453 |
| SIAH3 | 0,051589 | 0,018919 | -1,44723 | 0,000154 | 0,000981 |
| ELFN1 | 1,874323 | 0,750426 | -1,32059 | 0,016073 | 0,03716 |
| TBX1 | 0,609183 | 0,244702 | -1,31585 | 0,001996 | 0,007221 |
| RASGEF1C | 0,155902 | 0,072456 | -1,10546 | 0,001736 | 0,006478 |
| DRD5 | 0,024656 | 0,011268 | -1,12974 | 0,001355 | 0,005308 |
| ELOVL4 | 1,375769 | 0,687236 | -1,00136 | 0,010651 | 0,026871 |
| C21orf58 | 2,371761 | 1,004495 | -1,23949 | 0,001169 | 0,00473 |
| AIRE | 0,08856 | 0,04294 | -1,04432 | 0,005739 | 0,01653 |
| LYPD3 | 4,035084 | 8,906291 | 1,142226 | 0,005537 | 0,016041 |
| MOBP | 0,020968 | 0,010023 | -1,06482 | 0,00375 | 0,0119 |
| SCARA5 | 2,856179 | 0,880853 | -1,69711 | 5,58E-05 | 0,000447 |
| CCDC188 | 1,062864 | 0,314193 | -1,75823 | 4,72E-08 | 2,03E-06 |
| UNC79 | 0,797671 | 0,212585 | -1,90776 | 0,004171 | 0,012886 |
| ADRB3 | 0,164727 | 0,081413 | -1,01675 | 0,001167 | 0,00473 |
| LMO3 | 0,916861 | 0,421583 | -1,12089 | 0,000255 | 0,001447 |
| GPR150 | 0,496484 | 0,213475 | -1,21768 | 9,25E-05 | 0,000661 |
| LMX1A | 0,028641 | 0,010496 | -1,44826 | 0,005672 | 0,016389 |
| PRLR | 1,488168 | 0,392257 | -1,92367 | 0,015191 | 0,035587 |
| UGT2B4 | 0,703705 | 0,083031 | -3,08325 | 0,018755 | 0,042114 |
| CLECL1 | 0,686903 | 0,311981 | -1,13865 | 0,000901 | 0,003857 |
| GSDMC | 0,410625 | 1,077532 | 1,391837 | 0,000343 | 0,001817 |
| LHX1 | 0,101013 | 0,214551 | 1,086773 | 0,009113 | 0,023839 |
| ANKS1B | 0,285232 | 0,085869 | -1,73192 | 0,001099 | 0,004502 |
| IL20RB | 2,640885 | 7,90315 | 1,581406 | 0,001603 | 0,006063 |
| CR2 | 11,49232 | 2,653928 | -2,11447 | 0,004616 | 0,013937 |
| ERO1B | 20,04241 | 8,698974 | -1,20414 | 0,014005 | 0,033414 |
| PARP15 | 1,579794 | 0,720038 | -1,13359 | 0,00303 | 0,010019 |
| TREML1 | 0,555774 | 0,271149 | -1,03541 | 9,53E-07 | 1,95E-05 |
| CYP26A1 | 0,367216 | 0,796476 | 1,117001 | 0,001329 | 0,005224 |
| LTB | 21,18542 | 10,37881 | -1,02943 | 0,0033 | 0,01074 |
| ATP1A2 | 1,365347 | 0,537997 | -1,3436 | 0,00023 | 0,001336 |
| CRB2 | 0,081997 | 0,030357 | -1,43354 | 6,09E-05 | 0,000476 |
| DUSP26 | 7,596159 | 1,71675 | -2,14559 | 0,001065 | 0,004405 |
| KCNK17 | 3,132253 | 0,972941 | -1,68678 | 0,000641 | 0,002957 |
| KCP | 1,466889 | 3,006963 | 1,035548 | 0,000387 | 0,001995 |
| CMA1 | 0,990041 | 0,251703 | -1,97577 | 1,62E-05 | 0,000171 |
| FAM218A | 0,425418 | 0,187664 | -1,18073 | 8,52E-07 | 1,79E-05 |
| SSTR2 | 4,086239 | 0,563241 | -2,85895 | 0,001076 | 0,004438 |
| IGF1 | 0,903425 | 0,289665 | -1,64102 | 0,000773 | 0,003427 |
| LYPD2 | 5,07425 | 16,20667 | 1,675321 | 0,00052 | 0,002509 |
| VPREB3 | 9,628728 | 1,301775 | -2,88687 | 0,000366 | 0,00191 |
| SCAMP5 | 11,7784 | 5,133027 | -1,19826 | 7,70E-05 | 0,000576 |
| MAGEC3 | 0,018586 | 0,008636 | -1,10572 | 0,002536 | 0,008754 |
| VIPR2 | 0,399776 | 0,193279 | -1,04851 | 0,000142 | 0,000923 |
| RBM11 | 0,521038 | 0,260514 | -1,00003 | 5,94E-06 | 7,82E-05 |
| TMEM145 | 2,269279 | 0,354121 | -2,67992 | 0,013891 | 0,033209 |
| FAM163A | 1,730937 | 0,306566 | -2,49728 | 8,71E-05 | 0,000631 |
| NXPH4 | 4,571352 | 0,609731 | -2,90638 | 0,016464 | 0,037838 |
| CHST8 | 0,981967 | 0,320613 | -1,61484 | 0,003427 | 0,011053 |
| DPYSL5 | 0,101198 | 0,046088 | -1,13472 | 0,001404 | 0,005442 |
| RSPO4 | 0,417427 | 0,078371 | -2,41313 | 0,000468 | 0,002302 |
| CD79A | 29,11588 | 6,272662 | -2,21466 | 0,009062 | 0,023711 |
| UTF1 | 0,037154 | 0,012934 | -1,52235 | 8,82E-06 | 0,000108 |
| GPR162 | 3,142775 | 1,166189 | -1,43024 | 5,22E-09 | 4,44E-07 |
| GADD45G | 18,16649 | 7,976146 | -1,18752 | 1,33E-05 | 0,000147 |
| BFSP2 | 0,125855 | 0,051939 | -1,27686 | 0,00879 | 0,023152 |
| SIT1 | 3,675124 | 1,347151 | -1,44788 | 0,000786 | 0,00346 |
| BEX2 | 18,49922 | 7,181434 | -1,36512 | 0,000258 | 0,00146 |
| GLYAT | 0,047408 | 0,018642 | -1,34659 | 0,013331 | 0,032123 |
| FAM71F1 | 0,046217 | 0,019541 | -1,24192 | 0,001032 | 0,004297 |
| MSH4 | 0,077758 | 0,036888 | -1,07585 | 0,016851 | 0,038561 |
| CRIP3 | 0,710297 | 0,239889 | -1,56605 | 1,84E-07 | 5,58E-06 |
| SPIC | 0,044761 | 0,014074 | -1,6692 | 0,013385 | 0,032238 |
| ATP1A3 | 3,640977 | 0,594142 | -2,61545 | 0,000164 | 0,001023 |
| RHOXF1 | 0,371567 | 0,142589 | -1,38176 | 5,62E-05 | 0,00045 |
| LRRC10B | 3,342309 | 0,81242 | -2,04055 | 0,000108 | 0,000746 |
| HSF5 | 0,048994 | 0,017422 | -1,49167 | 2,76E-06 | 4,32E-05 |
| PIPOX | 2,054336 | 0,496041 | -2,05014 | 0,001619 | 0,00612 |
| ZBTB32 | 0,41878 | 0,207067 | -1,0161 | 5,52E-05 | 0,000443 |
| APLP1 | 38,02929 | 6,458206 | -2,55791 | 0,012277 | 0,030076 |
| YIPF7 | 0,04326 | 0,020028 | -1,11102 | 0,016378 | 0,037713 |
| HSPB6 | 32,58748 | 15,48237 | -1,07369 | 0,000392 | 0,002014 |
| ASPDH | 2,150247 | 0,392837 | -2,4525 | 0,000233 | 0,001348 |
| C22orf42 | 1,526204 | 0,208288 | -2,8733 | 0,013496 | 0,032427 |
| CER1 | 0,041849 | 0,012514 | -1,74168 | 0,008068 | 0,02169 |
| SLC16A11 | 1,823682 | 0,620321 | -1,55577 | 7,51E-06 | 9,46E-05 |
| PDK4 | 44,63032 | 17,75726 | -1,32961 | 0,000335 | 0,001784 |
| KIAA0319 | 0,973971 | 0,411908 | -1,24156 | 0,001702 | 0,006369 |
| CELF4 | 1,072821 | 0,28903 | -1,89212 | 0,004132 | 0,012798 |
| EREG | 1,006408 | 3,483375 | 1,791271 | 3,12E-08 | 1,51E-06 |
| CHI3L2 | 1,783951 | 0,781076 | -1,19154 | 0,002675 | 0,009075 |
| NFATC1 | 5,028388 | 1,932942 | -1,3793 | 5,10E-06 | 6,92E-05 |
| NSG2 | 1,740376 | 0,437753 | -1,99121 | 0,005638 | 0,016293 |
| RP1 | 0,027371 | 0,066509 | 1,280897 | 3,14E-05 | 0,000284 |
| SPDYC | 0,224457 | 0,568593 | 1,340959 | 0,016646 | 0,038205 |
| CFAP161 | 0,13016 | 0,059407 | -1,13157 | 0,016863 | 0,038561 |
| HCN2 | 1,296972 | 0,397516 | -1,70606 | 0,001145 | 0,004653 |
| LY6H | 6,829501 | 0,646369 | -3,40135 | 6,25E-05 | 0,000485 |
| TTC23L | 0,15882 | 0,077338 | -1,03814 | 0,002753 | 0,009294 |
| POU3F2 | 0,028865 | 0,012126 | -1,2512 | 0,006235 | 0,017677 |
| ACTL6B | 2,398411 | 0,268636 | -3,15836 | 0,001418 | 0,005489 |
| STPG4 | 0,174449 | 0,354835 | 1,024344 | 1,36E-06 | 2,56E-05 |
| RIMS4 | 0,23445 | 0,050347 | -2,21931 | 0,001618 | 0,00612 |
| ATP2B2 | 0,452427 | 0,042355 | -3,41707 | 0,001032 | 0,004297 |
| TMEM95 | 0,024379 | 0,009593 | -1,34555 | 0,001073 | 0,004435 |
| MYT1 | 0,975455 | 0,149514 | -2,7058 | 0,001099 | 0,004502 |
| UCHL1 | 22,70156 | 8,588587 | -1,4023 | 0,000252 | 0,001433 |
| SIRPG | 1,590028 | 0,787052 | -1,01452 | 0,003526 | 0,011311 |
| P2RY8 | 3,455213 | 1,630919 | -1,08309 | 1,56E-05 | 0,000166 |
| PLIN1 | 3,698405 | 0,74566 | -2,31031 | 2,33E-06 | 3,83E-05 |
| NUP210L | 0,071889 | 0,020488 | -1,81096 | 0,000588 | 0,00276 |
| KIAA1324 | 23,939 | 8,563364 | -1,48311 | 0,004574 | 0,013831 |
| PRRT3 | 2,369739 | 1,053165 | -1,17 | 0,00373 | 0,01184 |
| SMIM32 | 10,47946 | 1,248942 | -3,06879 | 0,000551 | 0,002622 |
| TNNT1 | 1,81487 | 7,525905 | 2,051999 | 2,00E-05 | 0,000202 |
| TCF23 | 0,226959 | 0,08286 | -1,45369 | 0,000582 | 0,002736 |
| KISS1R | 0,735206 | 0,122391 | -2,58665 | 0,012474 | 0,030457 |
| LINGO3 | 0,648435 | 0,138614 | -2,22589 | 6,68E-09 | 5,19E-07 |
| WNT10B | 0,638876 | 0,218408 | -1,54851 | 1,85E-05 | 0,000189 |
| GPR83 | 0,123682 | 0,059157 | -1,064 | 0,001825 | 0,006738 |
| AC113554.1 | 0,021655 | 0,003847 | -2,49294 | 0,005151 | 0,015198 |
| ETV3L | 0,01703 | 0,006947 | -1,29368 | 0,015895 | 0,036904 |
| AMY2B | 33,62328 | 16,21572 | -1,05207 | 0,022542 | 0,048548 |
| NECAB2 | 2,80442 | 0,695065 | -2,01248 | 0,001957 | 0,00712 |
| TBC1D10C | 3,731434 | 1,29731 | -1,52421 | 7,73E-07 | 1,65E-05 |
| SLC18A1 | 0,536368 | 0,111616 | -2,26468 | 0,000914 | 0,003901 |
| NAP1L2 | 2,284298 | 0,966609 | -1,24075 | 0,000191 | 0,001154 |
| ZNF540 | 1,229067 | 0,576013 | -1,09339 | 4,98E-07 | 1,19E-05 |
| HFM1 | 0,127097 | 0,054853 | -1,21228 | 2,11E-05 | 0,00021 |
| CNMD | 0,433177 | 0,162001 | -1,41896 | 0,004344 | 0,013301 |
| COL2A1 | 0,236623 | 0,020858 | -3,50393 | 0,011481 | 0,028534 |
| MUC21 | 0,123561 | 0,826284 | 2,74142 | 0,000145 | 0,000937 |
| CEACAM19 | 2,951458 | 1,337189 | -1,14222 | 0,000594 | 0,002785 |
| SFRP1 | 10,16179 | 3,491084 | -1,54141 | 0,000621 | 0,002885 |
| ZC3H12D | 0,676211 | 0,295969 | -1,19203 | 7,80E-05 | 0,00058 |
| GRIA1 | 0,251206 | 0,021185 | -3,56773 | 0,003232 | 0,010577 |
| KIF12 | 33,12596 | 16,47384 | -1,00779 | 0,015563 | 0,036282 |
| SAXO2 | 0,523848 | 0,237553 | -1,1409 | 0,006334 | 0,017897 |
| HES2 | 0,476746 | 0,997144 | 1,064579 | 0,000527 | 0,002531 |
| CD52 | 51,36666 | 20,96288 | -1,293 | 0,00041 | 0,002087 |
| DPY19L2 | 0,893983 | 0,425799 | -1,07008 | 0,000155 | 0,000981 |
| ACAP1 | 3,65724 | 1,423389 | -1,36143 | 4,72E-08 | 2,03E-06 |
| RAB26 | 7,165068 | 2,559377 | -1,48519 | 0,001702 | 0,006369 |
| PRR20G | 0,039526 | 0,12355 | 1,644207 | 0,012049 | 0,029676 |
| TUNAR | 2,27208 | 0,886346 | -1,35807 | 0,021354 | 0,046573 |
| TPH2 | 0,034106 | 0,007398 | -2,20473 | 0,007944 | 0,021412 |
| MEDAG | 16,13697 | 6,961757 | -1,21285 | 0,001976 | 0,007175 |
| SBK2 | 0,684214 | 0,103237 | -2,72849 | 0,006347 | 0,017931 |
| PPP4R4 | 0,609525 | 0,14018 | -2,12041 | 0,011777 | 0,029134 |
| RIPOR2 | 2,083352 | 0,821526 | -1,34253 | 0,011391 | 0,028351 |
| PSD | 5,459384 | 2,501251 | -1,12609 | 0,007043 | 0,019466 |
| LRRN3 | 0,93145 | 0,368362 | -1,33835 | 0,000761 | 0,003384 |
| SLC22A17 | 25,18885 | 6,111936 | -2,04308 | 7,43E-05 | 0,000557 |
| RADIL | 0,869869 | 0,232567 | -1,90315 | 2,77E-07 | 7,60E-06 |
| RTN4RL1 | 2,37527 | 1,12312 | -1,08058 | 0,004094 | 0,012718 |
| FXYD7 | 0,355058 | 0,106907 | -1,73169 | 2,64E-06 | 4,21E-05 |
| JAKMIP2 | 0,888544 | 0,373839 | -1,24902 | 0,002973 | 0,009862 |
| SPTB | 0,649551 | 0,272438 | -1,25352 | 0,000392 | 0,002014 |
| KCNK3 | 4,887783 | 2,208867 | -1,14587 | 0,000575 | 0,002715 |
| C1orf194 | 0,602194 | 0,167291 | -1,84787 | 0,000387 | 0,001995 |
| NPPC | 0,513606 | 0,106283 | -2,27275 | 8,22E-06 | 0,000102 |
| PRODH2 | 0,64264 | 0,033998 | -4,24047 | 0,000108 | 0,000744 |
| LGI4 | 2,073186 | 1,030882 | -1,00797 | 0,000158 | 0,000997 |
| C2orf66 | 0,136013 | 0,064171 | -1,08377 | 9,04E-06 | 0,00011 |
| CHRNA4 | 0,011186 | 0,002474 | -2,17697 | 0,011187 | 0,027989 |
| HBA2 | 11,66502 | 4,840482 | -1,26897 | 0,01755 | 0,039826 |
| PAX5 | 1,464389 | 0,253041 | -2,53285 | 0,003147 | 0,010344 |
| CRHBP | 0,300054 | 0,087793 | -1,77304 | 9,69E-08 | 3,41E-06 |
| RDH12 | 2,304656 | 0,744611 | -1,62999 | 0,012277 | 0,030076 |
| TNFRSF13C | 3,957795 | 0,698217 | -2,50295 | 0,001899 | 0,006956 |
| PRRT4 | 0,181211 | 0,074025 | -1,29159 | 4,05E-05 | 0,000349 |
| FAM83A | 5,482358 | 11,80971 | 1,107105 | 2,00E-05 | 0,000202 |
| TTC24 | 0,151257 | 0,053027 | -1,51221 | 0,003281 | 0,010696 |
| DEFB124 | 0,160953 | 0,079331 | -1,02069 | 0,000152 | 0,000969 |
| C1QTNF9 | 0,110728 | 0,050726 | -1,12623 | 1,17E-07 | 3,88E-06 |
| FOXN4 | 0,020307 | 0,006054 | -1,7461 | 0,014902 | 0,035044 |
| ITGB6 | 25,13947 | 55,38017 | 1,139415 | 1,31E-08 | 8,14E-07 |
| MLXIPL | 8,756896 | 3,923511 | -1,15827 | 0,009378 | 0,024342 |
| AMER2 | 0,082907 | 0,007469 | -3,47247 | 0,000196 | 0,001179 |
| MAPK8IP2 | 3,706133 | 1,130232 | -1,7133 | 0,002675 | 0,009075 |
| SYNM | 9,568046 | 3,846804 | -1,31456 | 0,001996 | 0,007221 |
| GPR142 | 1,703708 | 0,299444 | -2,50832 | 0,002523 | 0,008716 |
| RIC3 | 1,249043 | 0,453302 | -1,46228 | 3,37E-05 | 0,0003 |
| DLL3 | 0,156841 | 0,068928 | -1,18615 | 0,005335 | 0,015625 |
| ST18 | 0,946997 | 0,21578 | -2,1338 | 0,018994 | 0,042483 |
| B4GALNT4 | 1,99046 | 0,933251 | -1,09276 | 4,49E-06 | 6,28E-05 |
| KCNJ11 | 4,653384 | 1,717907 | -1,43763 | 0,010473 | 0,026569 |
| MROH6 | 6,686424 | 13,45774 | 1,00913 | 6,46E-06 | 8,39E-05 |
| ARX | 5,799283 | 0,719946 | -3,00991 | 0,005057 | 0,014985 |
| CTNNA2 | 0,430269 | 0,160137 | -1,42593 | 0,004745 | 0,014236 |
| NKAIN2 | 0,176545 | 0,059306 | -1,57379 | 0,000101 | 0,000707 |
| TMCC2 | 1,28263 | 0,619858 | -1,0491 | 4,89E-06 | 6,69E-05 |
| NCR3 | 1,158658 | 0,370447 | -1,64512 | 0,000261 | 0,00147 |
| CACNA2D3 | 0,957818 | 0,360344 | -1,41038 | 0,00096 | 0,004054 |
| PRIMA1 | 1,621014 | 0,595631 | -1,44441 | 0,013333 | 0,032123 |
| KCNA5 | 3,762894 | 0,674748 | -2,47942 | 0,000539 | 0,002574 |
| PLIN4 | 6,099585 | 1,860553 | -1,71298 | 0,004249 | 0,013077 |
| SLC25A27 | 2,705787 | 1,289455 | -1,06929 | 3,39E-06 | 5,03E-05 |
| CWH43 | 0,03836 | 0,164464 | 2,100082 | 0,014478 | 0,034277 |
| BEX1 | 52,48983 | 11,23391 | -2,22418 | 0,001022 | 0,004261 |
| PCSK1N | 145,4789 | 17,25835 | -3,07544 | 0,001449 | 0,005579 |
| GH1 | 0,038248 | 0,011033 | -1,79352 | 5,94E-06 | 7,82E-05 |
| CCL17 | 10,71628 | 4,671233 | -1,19793 | 0,001524 | 0,005821 |
| SLC7A10 | 0,172275 | 0,021787 | -2,98315 | 4,33E-06 | 6,06E-05 |
| ASCL2 | 2,559143 | 0,773321 | -1,72652 | 0,000111 | 0,000759 |
| DDX25 | 0,266478 | 0,070781 | -1,91259 | 0,000238 | 0,001372 |
| KCNK12 | 0,124574 | 0,050782 | -1,29462 | 9,74E-06 | 0,000117 |
| RAB3C | 1,48488 | 0,67326 | -1,14111 | 0,000714 | 0,003213 |
| TNMD | 0,274121 | 0,050984 | -2,4267 | 3,81E-05 | 0,000333 |
| GNB3 | 0,838917 | 0,416456 | -1,01037 | 1,15E-05 | 0,000131 |
| ASIC4 | 0,138396 | 0,042638 | -1,69858 | 0,003981 | 0,012429 |
| CARTPT | 5,987786 | 0,582085 | -3,36272 | 0,011523 | 0,02862 |
| LCN6 | 0,384405 | 0,125417 | -1,61589 | 5,63E-10 | 9,72E-08 |
| KCNA1 | 0,09997 | 0,021215 | -2,23642 | 0,005375 | 0,015717 |
| C16orf89 | 4,711445 | 2,046571 | -1,20296 | 3,00E-05 | 0,000274 |
| TMEM151B | 0,194878 | 0,058965 | -1,72464 | 0,000385 | 0,001988 |
| CACNA2D2 | 5,653256 | 1,373154 | -2,04159 | 0,000473 | 0,002319 |
| ST8SIA5 | 0,050281 | 0,022258 | -1,17571 | 0,002358 | 0,008254 |
| NLGN4X | 0,944711 | 0,434501 | -1,12051 | 0,003593 | 0,011492 |
| MAPK4 | 0,704332 | 0,274034 | -1,3619 | 0,010561 | 0,026742 |
| C9orf24 | 2,517902 | 0,843218 | -1,57825 | 0,005245 | 0,015421 |
| NUPR2 | 2,132494 | 0,9561 | -1,15731 | 0,021179 | 0,046298 |
| AMER3 | 0,941813 | 0,195146 | -2,27089 | 0,004056 | 0,012626 |
| KIF5C | 2,140465 | 0,851905 | -1,32916 | 0,014352 | 0,034025 |
| CEACAM18 | 0,529694 | 1,141222 | 1,107347 | 0,018766 | 0,042133 |
| KLKB1 | 1,486797 | 0,578611 | -1,36154 | 0,000273 | 0,001521 |
| VGF | 16,21118 | 3,62958 | -2,15911 | 0,010039 | 0,025698 |
| S100Z | 0,326689 | 0,13267 | -1,30007 | 0,000478 | 0,00234 |
| DLGAP3 | 0,671434 | 0,229051 | -1,55158 | 1,03E-05 | 0,000121 |
| LPL | 7,749284 | 3,097484 | -1,32297 | 1,64E-05 | 0,000173 |
| CELF3 | 7,071504 | 1,185664 | -2,57632 | 0,006222 | 0,017642 |
| KCNC2 | 0,102748 | 0,015627 | -2,71698 | 0,001581 | 0,006004 |
| CD40LG | 1,093434 | 0,524348 | -1,06027 | 0,00447 | 0,013586 |
| VIT | 0,507773 | 0,216308 | -1,2311 | 0,00037 | 0,001929 |
| KLHL14 | 0,326788 | 0,071695 | -2,1884 | 0,000264 | 0,001483 |
| NPAP1 | 0,009852 | 0,003935 | -1,32415 | 0,007145 | 0,019726 |
| ATP6V0E2 | 15,14839 | 7,006673 | -1,11236 | 2,05E-06 | 3,47E-05 |
| GNG7 | 4,641035 | 2,082341 | -1,15624 | 4,14E-08 | 1,82E-06 |
| ARPP21 | 0,015421 | 0,005655 | -1,44735 | 0,022999 | 0,049422 |
| NPAS4 | 0,170615 | 0,044332 | -1,94431 | 0,004875 | 0,014547 |
| MTMR7 | 0,863607 | 0,349861 | -1,30359 | 0,000452 | 0,002251 |
| KIF19 | 1,39845 | 0,240732 | -2,53833 | 3,48E-06 | 5,11E-05 |
| GCK | 1,929877 | 0,536717 | -1,84628 | 0,018115 | 0,040917 |
| GABRB1 | 0,044816 | 0,021357 | -1,06926 | 0,004411 | 0,013436 |
| DNAH3 | 0,097791 | 0,221701 | 1,18084 | 1,01E-08 | 6,81E-07 |
| NYAP1 | 1,742943 | 0,694197 | -1,32811 | 0,00421 | 0,012972 |
| OLFM1 | 5,730109 | 2,056443 | -1,47841 | 0,00035 | 0,001846 |
| PTGDR2 | 1,133505 | 0,475163 | -1,2543 | 0,004532 | 0,013724 |
| P2RX2 | 0,251033 | 0,097203 | -1,36881 | 0,003511 | 0,01129 |
| NPW | 3,161581 | 0,393597 | -3,00585 | 0,001211 | 0,004865 |
| THRSP | 1,077056 | 0,233002 | -2,20868 | 0,005435 | 0,015835 |
| NOVA1 | 0,789795 | 0,241383 | -1,71016 | 2,11E-05 | 0,00021 |
| SLITRK1 | 0,229319 | 0,036911 | -2,63522 | 0,00038 | 0,001968 |
| CCL14 | 0,809986 | 0,329874 | -1,29598 | 7,88E-09 | 5,80E-07 |
| CEACAM20 | 0,093076 | 0,350987 | 1,914945 | 0,013595 | 0,032633 |
| TSPAN11 | 3,553344 | 1,491531 | -1,25238 | 0,000737 | 0,003302 |
| FBLL1 | 3,51136 | 0,621535 | -2,49812 | 4,82E-06 | 6,63E-05 |
| PPP1R16B | 3,667436 | 1,747626 | -1,06937 | 0,000462 | 0,002287 |
| NEURL1 | 7,994782 | 2,125443 | -1,91129 | 0,000108 | 0,000746 |
| ARL14 | 15,8515 | 41,21161 | 1,378432 | 1,11E-06 | 2,19E-05 |
| IL1RAPL2 | 0,096395 | 0,210309 | 1,125475 | 0,000299 | 0,001632 |
| DUSP15 | 0,82025 | 0,284933 | -1,52544 | 5,05E-07 | 1,20E-05 |
| CFP | 1,300978 | 0,556561 | -1,22498 | 2,72E-07 | 7,50E-06 |
| GRIA2 | 0,606872 | 0,159826 | -1,92489 | 0,012482 | 0,030457 |
| LBP | 2,351323 | 0,475461 | -2,30607 | 0,000227 | 0,001325 |
| RNF225 | 0,022686 | 0,067525 | 1,573614 | 1,16E-05 | 0,000132 |
| GRM7 | 0,072151 | 0,033926 | -1,08864 | 0,005438 | 0,015835 |
| ADIPOQ | 3,487138 | 0,395281 | -3,14109 | 7,85E-05 | 0,000583 |
| LHFPL1 | 0,064363 | 0,024494 | -1,39379 | 0,00589 | 0,016898 |
| ICAM3 | 1,341228 | 0,593276 | -1,17678 | 0,001322 | 0,005199 |
| WNK4 | 2,147659 | 0,74043 | -1,53633 | 0,014826 | 0,034876 |
| JPH3 | 0,888407 | 0,282113 | -1,65495 | 0,02119 | 0,046298 |
| PCDHA1 | 0,116496 | 0,039456 | -1,56197 | 0,005145 | 0,015198 |
| WNT9B | 0,089572 | 0,043018 | -1,05809 | 5,01E-05 | 0,000413 |
| SNX22 | 1,127255 | 0,457611 | -1,30062 | 8,67E-09 | 6,20E-07 |
| GPR88 | 0,218707 | 0,078478 | -1,47863 | 0,000655 | 0,003006 |
| KRT73 | 0,034992 | 0,006961 | -2,32958 | 0,000882 | 0,003797 |
| RLBP1 | 0,081 | 0,022256 | -1,86375 | 0,016765 | 0,038397 |
| FCRL2 | 0,866945 | 0,181473 | -2,25619 | 0,005148 | 0,015198 |
| VSX2 | 0,01415 | 0,003767 | -1,90916 | 0,000569 | 0,00269 |
| CHD5 | 0,575532 | 0,195599 | -1,557 | 0,002649 | 0,009012 |
| RALYL | 0,629342 | 0,305366 | -1,0433 | 0,003726 | 0,01184 |
| CD1C | 5,781261 | 2,628775 | -1,13699 | 0,000111 | 0,000759 |
| COL25A1 | 0,144294 | 0,06001 | -1,26574 | 0,010384 | 0,026389 |
| RASAL3 | 4,38293 | 1,98002 | -1,14638 | 3,44E-06 | 5,07E-05 |
| CADM3 | 2,135765 | 0,815119 | -1,38967 | 1,03E-05 | 0,000121 |
| ARRDC5 | 0,384011 | 0,166149 | -1,20867 | 1,80E-05 | 0,000186 |
| RNF182 | 0,580755 | 0,15127 | -1,9408 | 0,009378 | 0,024342 |
| ANKRD55 | 0,259332 | 0,10074 | -1,36416 | 0,000222 | 0,0013 |
| LRRC74A | 0,088806 | 0,038547 | -1,20404 | 3,81E-05 | 0,000333 |
| NANOS1 | 1,089601 | 0,466943 | -1,22248 | 0,000306 | 0,001663 |
| FBXL16 | 3,637166 | 1,189731 | -1,61218 | 0,001122 | 0,004574 |
| HAPLN2 | 0,267521 | 0,057751 | -2,21173 | 0,002833 | 0,009518 |
| TAL2 | 0,146345 | 0,054264 | -1,43132 | 1,42E-06 | 2,65E-05 |
| GRIK5 | 2,670528 | 0,75352 | -1,82541 | 3,08E-05 | 0,00028 |
| REM2 | 0,797518 | 0,333129 | -1,25943 | 2,37E-06 | 3,87E-05 |
| MPP2 | 2,13388 | 0,705629 | -1,5965 | 0,002973 | 0,009862 |
| IGFL1 | 2,445331 | 5,537934 | 1,179318 | 0,018731 | 0,042067 |
| NAT8L | 1,636316 | 0,599875 | -1,44772 | 2,44E-05 | 0,000234 |
| MET | 14,92053 | 30,78415 | 1,044889 | 3,96E-13 | 7,37E-10 |
| GTSF1L | 0,078005 | 0,034527 | -1,17586 | 0,003315 | 0,010787 |
| CIB4 | 0,18653 | 0,084795 | -1,13736 | 0,003797 | 0,012016 |
| MSI1 | 3,146631 | 0,844057 | -1,8984 | 1,18E-05 | 0,000133 |
| WNK3 | 0,259629 | 0,115652 | -1,16666 | 0,001206 | 0,004845 |
| SPAG6 | 0,734167 | 0,132145 | -2,47399 | 0,012168 | 0,029903 |
| JAKMIP1 | 1,01885 | 0,352522 | -1,53116 | 4,03E-05 | 0,000347 |
| PHF24 | 0,176942 | 0,074051 | -1,25668 | 2,98E-06 | 4,58E-05 |
| GPD1 | 6,67864 | 3,00599 | -1,15171 | 0,007296 | 0,020026 |
| SLC4A8 | 0,755715 | 0,284536 | -1,40923 | 0,005293 | 0,015522 |
| CLCN1 | 0,365796 | 0,847005 | 1,211332 | 6,98E-05 | 0,000533 |
| ESPN | 2,008092 | 4,067014 | 1,018145 | 8,05E-06 | 1,00E-04 |
| EFCAB8 | 0,069086 | 0,028062 | -1,29977 | 1,46E-05 | 0,000157 |
| ACSL6 | 0,365996 | 0,080241 | -2,18942 | 5,21E-08 | 2,15E-06 |
| PAK3 | 2,604411 | 0,831434 | -1,64728 | 0,006505 | 0,018286 |
| CXCL11 | 1,69732 | 4,150244 | 1,289938 | 0,010651 | 0,026871 |
| PEMT | 22,57395 | 8,285438 | -1,44601 | 0,001349 | 0,005286 |
| SMIM23 | 0,094195 | 0,036378 | -1,37256 | 0,001648 | 0,006222 |
| LAMB4 | 0,141503 | 0,045307 | -1,64304 | 0,000289 | 0,001592 |
| ADGRG5 | 2,832327 | 0,92073 | -1,62114 | 0,000721 | 0,003241 |
| RIMBP3C | 0,009792 | 0,004738 | -1,04724 | 0,017596 | 0,039925 |
| CLEC17A | 1,069702 | 0,214029 | -2,32133 | 0,000375 | 0,001947 |
| A2ML1 | 1,233313 | 2,80551 | 1,185724 | 0,021027 | 0,046025 |
| NCAM1 | 3,167347 | 0,982627 | -1,68856 | 0,000588 | 0,00276 |
| C10orf82 | 0,081403 | 0,035361 | -1,20293 | 0,000178 | 0,001093 |
| FNDC5 | 0,9109 | 0,385109 | -1,24202 | 7,16E-05 | 0,000542 |
| ARHGAP9 | 4,184776 | 1,906927 | -1,1339 | 3,34E-06 | 4,98E-05 |
| ASB16 | 1,128421 | 0,55905 | -1,01326 | 0,000401 | 0,002053 |
| SLC22A16 | 0,131588 | 0,052384 | -1,32884 | 5,45E-05 | 0,000439 |
| GP1BA | 1,107259 | 0,414639 | -1,41706 | 1,09E-05 | 0,000126 |
| KCNB1 | 0,665894 | 0,143871 | -2,21052 | 0,000225 | 0,001312 |
| TCAP | 1,030937 | 0,468175 | -1,13884 | 7,72E-06 | 9,68E-05 |
| AMPH | 1,883535 | 0,816184 | -1,20648 | 0,015689 | 0,036482 |
| GDAP1L1 | 0,713941 | 0,141968 | -2,33024 | 0,001336 | 0,005241 |
| MAP6 | 3,539712 | 1,381913 | -1,35696 | 0,001032 | 0,004297 |
| KRT72 | 0,138921 | 0,017259 | -3,00887 | 5,77E-05 | 0,000458 |
| FCRLA | 2,567256 | 0,38606 | -2,73333 | 0,000665 | 0,00304 |
| FMN2 | 0,809751 | 0,270031 | -1,58435 | 0,011777 | 0,029134 |
| PCDH15 | 0,034359 | 0,004023 | -3,09426 | 0,001222 | 0,0049 |
| PI16 | 6,865069 | 2,182505 | -1,65329 | 8,50E-06 | 0,000104 |
| TMEM179 | 5,693794 | 0,688585 | -3,04768 | 0,012431 | 0,030379 |
| ELANE | 6,768594 | 0,26046 | -4,69972 | 0,003146 | 0,010344 |
| BTNL2 | 0,033665 | 0,011261 | -1,57993 | 0,002012 | 0,007272 |
| TCEAL6 | 0,483129 | 0,23709 | -1,02697 | 0,022165 | 0,047967 |
| TNR | 0,329635 | 0,112395 | -1,55229 | 0,005151 | 0,015198 |
| MAPK10 | 1,660663 | 0,78955 | -1,07266 | 0,000258 | 0,00146 |
| ADH1B | 10,28335 | 4,123647 | -1,31832 | 3,39E-05 | 0,000302 |
| GALNT5 | 5,743479 | 11,74893 | 1,032533 | 3,44E-08 | 1,61E-06 |
| C2CD4C | 1,270888 | 0,562351 | -1,1763 | 0,013443 | 0,032305 |
| HAP1 | 2,34485 | 0,25836 | -3,18204 | 0,004788 | 0,014342 |
| CD300LG | 0,239741 | 0,047219 | -2,34403 | 7,83E-07 | 1,67E-05 |
| SCN3A | 0,328979 | 0,147719 | -1,15514 | 0,000324 | 0,001738 |
| ARL14EPL | 0,027364 | 0,069697 | 1,348812 | 0,007164 | 0,019758 |
| ZAP70 | 2,544831 | 0,932624 | -1,4482 | 5,54E-06 | 7,40E-05 |
| TMEM132E | 0,865364 | 0,312439 | -1,46973 | 0,008243 | 0,022016 |
| HRH3 | 0,085416 | 0,008508 | -3,32769 | 0,014971 | 0,035163 |
| MCOLN3 | 1,147716 | 0,494228 | -1,21552 | 0,004132 | 0,012798 |
| UGT1A10 | 2,712625 | 7,643843 | 1,494609 | 5,20E-08 | 2,15E-06 |
| MYL7 | 0,227665 | 0,041291 | -2,46303 | 0,001826 | 0,006738 |
| RASGRP2 | 3,763332 | 1,044054 | -1,84981 | 3,33E-07 | 8,65E-06 |
| XKR7 | 0,646611 | 0,121079 | -2,41695 | 0,010919 | 0,027423 |
| TRIM67 | 0,094423 | 0,038795 | -1,28327 | 0,001256 | 0,005001 |
| JSRP1 | 3,089118 | 1,507501 | -1,03504 | 0,000252 | 0,001433 |
| TMEM272 | 0,064048 | 0,03131 | -1,03254 | 0,004633 | 0,013983 |
| GAMT | 15,61265 | 7,650355 | -1,02912 | 1,46E-05 | 0,000157 |
| RGS7 | 0,998996 | 0,48476 | -1,04321 | 0,007622 | 0,020703 |
| BEND4 | 0,133684 | 0,032205 | -2,05347 | 0,000426 | 0,002147 |
| GRM4 | 0,379587 | 0,125015 | -1,60232 | 0,013062 | 0,031621 |
| SNTG2 | 0,253711 | 0,109268 | -1,21532 | 6,74E-07 | 1,50E-05 |
| SCG2 | 89,12073 | 17,51645 | -2,34705 | 0,007043 | 0,019466 |
| TMEM178B | 1,564245 | 0,546735 | -1,51655 | 0,006334 | 0,017897 |
| DPPA4 | 0,03581 | 0,017111 | -1,06542 | 0,001437 | 0,005544 |
| FDCSP | 85,02988 | 11,73173 | -2,85755 | 0,015688 | 0,036482 |
| TFF2 | 180,2776 | 462,2463 | 1,358442 | 0,012379 | 0,030273 |
| SLC2A4 | 0,82361 | 0,361219 | -1,18909 | 0,001635 | 0,006177 |
| PYHIN1 | 0,874002 | 0,431151 | -1,01945 | 0,002404 | 0,008384 |
| NRG2 | 0,444004 | 0,185106 | -1,26222 | 2,14E-06 | 3,58E-05 |
| TSPOAP1 | 2,212674 | 1,103254 | -1,00403 | 5,47E-06 | 7,31E-05 |
| ASTN1 | 0,562032 | 0,132792 | -2,08149 | 7,99E-05 | 0,000591 |
| CD3D | 11,321 | 5,512294 | -1,03828 | 0,002916 | 0,009731 |
| RTL5 | 4,630217 | 2,051937 | -1,17409 | 0,000669 | 0,003052 |
| SLC7A2 | 10,59692 | 4,871743 | -1,12113 | 0,002476 | 0,008572 |
| SLC6A13 | 0,079786 | 0,024016 | -1,73211 | 0,000219 | 0,001289 |
| RTP5 | 0,137839 | 0,033828 | -2,0267 | 1,30E-06 | 2,48E-05 |
| MAG | 0,084213 | 0,031344 | -1,42587 | 0,00036 | 0,001882 |
| RAB39B | 1,457902 | 0,393519 | -1,88939 | 1,12E-06 | 2,22E-05 |
| DNAH9 | 0,179396 | 0,056548 | -1,66559 | 0,000911 | 0,00389 |
| NT5M | 1,368697 | 0,678141 | -1,01315 | 7,68E-09 | 5,70E-07 |
| SERPINF2 | 7,121989 | 2,467301 | -1,52935 | 1,22E-09 | 1,68E-07 |
| FCRL1 | 1,610289 | 0,214905 | -2,90555 | 0,002448 | 0,008512 |
| COL19A1 | 0,087881 | 0,024375 | -1,85015 | 0,001224 | 0,004907 |
| ANKRD18B | 0,044585 | 0,098971 | 1,150452 | 0,002619 | 0,008959 |
| B3GAT1 | 0,591172 | 0,117474 | -2,33124 | 0,000103 | 0,000721 |
| SNAP91 | 1,286072 | 0,373347 | -1,78438 | 0,000259 | 0,001468 |
| ZDHHC22 | 0,1927 | 0,044819 | -2,10417 | 0,015229 | 0,035671 |
| PNMA6A | 1,413495 | 0,633287 | -1,15834 | 0,003395 | 0,010982 |
| CACNA1B | 0,722864 | 0,099913 | -2,85498 | 0,002213 | 0,007848 |
| FCER2 | 3,908246 | 0,325878 | -3,58412 | 0,00017 | 0,001053 |
| GNG4 | 4,607545 | 1,856655 | -1,31129 | 0,010923 | 0,027423 |
| C14orf178 | 0,056355 | 0,023974 | -1,23307 | 0,00045 | 0,002244 |
| HSD17B3 | 0,637572 | 0,264605 | -1,26875 | 1,01E-05 | 0,00012 |
| FGF23 | 0,005557 | 0,027224 | 2,292608 | 0,001901 | 0,006959 |
| LRRC8E | 0,172275 | 0,344928 | 1,001577 | 1,16E-11 | 8,13E-09 |
| CNTNAP4 | 0,113942 | 0,010663 | -3,41766 | 7,06E-05 | 0,000537 |
| EFR3B | 0,789286 | 0,272879 | -1,53229 | 0,001206 | 0,004845 |
| CTSG | 2,68472 | 0,942357 | -1,51043 | 0,000306 | 0,001663 |
| CCL21 | 100,5154 | 32,86076 | -1,61298 | 0,000648 | 0,002981 |
| PSMA8 | 0,064093 | 0,014602 | -2,13402 | 0,001289 | 0,005097 |
| PRB1 | 0,053508 | 0,322929 | 2,593396 | 0,011704 | 0,029008 |
| EPHA2 | 30,88227 | 61,85097 | 1,002017 | 7,15E-10 | 1,13E-07 |
| CDK5R2 | 4,748104 | 0,887811 | -2,41903 | 0,008386 | 0,022327 |
| CADPS | 2,594902 | 1,129838 | -1,19956 | 0,01855 | 0,041684 |
| CMTM5 | 0,197604 | 0,059662 | -1,72772 | 9,30E-05 | 0,000664 |
| BRINP2 | 0,210774 | 0,036117 | -2,54495 | 0,000179 | 0,001096 |
| PLCXD3 | 2,745825 | 1,289978 | -1,08989 | 0,010923 | 0,027423 |
| FUT7 | 0,34342 | 0,163315 | -1,07232 | 3,02E-06 | 4,63E-05 |
| GALNT16 | 1,241193 | 0,539883 | -1,20101 | 1,07E-07 | 3,66E-06 |
| SERTM2 | 0,067151 | 0,024269 | -1,46827 | 0,002162 | 0,0077 |
| KL | 3,44175 | 0,565103 | -2,60656 | 0,004745 | 0,014236 |
| CXCL13 | 21,15697 | 5,40649 | -1,96837 | 0,014706 | 0,034663 |
| RPRML | 0,452049 | 0,019916 | -4,50448 | 5,41E-05 | 0,000437 |
| DMRTC1B | 0,029535 | 0,007365 | -2,00375 | 0,001809 | 0,006688 |
| CALY | 14,15063 | 1,13485 | -3,64029 | 0,002138 | 0,007631 |
| RASA4 | 0,213397 | 0,093828 | -1,18545 | 2,19E-07 | 6,36E-06 |
| SLC8A3 | 0,215662 | 0,069817 | -1,62713 | 8,19E-05 | 0,000603 |
| ZBTB8B | 0,021137 | 0,009395 | -1,16982 | 0,01322 | 0,031913 |
| HEPACAM | 0,049614 | 0,015835 | -1,64765 | 0,001443 | 0,005564 |
| NKX6-3 | 5,881255 | 0,397916 | -3,88559 | 0,007151 | 0,019738 |
| ABRA | 0,044996 | 0,021191 | -1,08636 | 0,003325 | 0,010815 |
| C12orf50 | 0,010579 | 0,004178 | -1,34027 | 0,002961 | 0,00985 |
| MMRN1 | 4,4403 | 2,190922 | -1,01912 | 0,000166 | 0,001031 |
| DES | 184,9876 | 52,20171 | -1,82526 | 0,015068 | 0,035343 |
| DNAI2 | 0,229242 | 0,024474 | -3,22757 | 2,01E-06 | 3,44E-05 |
| PRSS21 | 3,237114 | 7,513912 | 1,214856 | 0,004921 | 0,014652 |
| RND2 | 1,060134 | 0,326663 | -1,69837 | 6,82E-06 | 8,77E-05 |
| CD36 | 4,234807 | 1,769344 | -1,25908 | 1,40E-06 | 2,62E-05 |
| CACNA1H | 7,858639 | 3,846873 | -1,03059 | 0,000777 | 0,003437 |
| CNTN2 | 0,126167 | 0,054798 | -1,20315 | 0,000324 | 0,001738 |
| RBP7 | 7,222299 | 3,345229 | -1,11035 | 1,52E-07 | 4,81E-06 |
| S100A1 | 2,528445 | 1,076335 | -1,23212 | 0,006505 | 0,018286 |
| SLC29A4 | 8,114744 | 2,23793 | -1,85838 | 0,000669 | 0,003052 |
| PAX6 | 1,920444 | 0,719579 | -1,41621 | 0,014706 | 0,034663 |
| GNAO1 | 3,036862 | 1,218154 | -1,31788 | 0,007824 | 0,021129 |
| KRTAP5-5 | 0,25872 | 0,093721 | -1,46494 | 0,015716 | 0,036539 |
| CHRNB2 | 0,902054 | 0,25038 | -1,84909 | 0,002159 | 0,007694 |
| TMEM74B | 3,960653 | 1,477621 | -1,42246 | 0,002159 | 0,007694 |
| CAMK2B | 2,87779 | 0,583347 | -2,30254 | 0,00421 | 0,012972 |
| PLA2G2A | 102,0553 | 37,87999 | -1,42984 | 0,002313 | 0,008124 |
| SSUH2 | 0,370024 | 0,129139 | -1,51869 | 0,001772 | 0,006577 |
| SCG5 | 129,2536 | 35,33274 | -1,87113 | 0,020066 | 0,044377 |
| CXXC4 | 2,172387 | 0,697755 | -1,63849 | 0,002549 | 0,008771 |
| TRPM6 | 0,156125 | 0,072426 | -1,10812 | 5,32E-06 | 7,14E-05 |
| AP3B2 | 1,059701 | 0,320007 | -1,72748 | 0,001231 | 0,004931 |
| RHBDL3 | 0,298342 | 0,148035 | -1,01103 | 8,52E-08 | 3,13E-06 |
| FFAR3 | 0,142534 | 0,062954 | -1,17893 | 0,000154 | 0,00098 |
| APCDD1L | 0,796943 | 2,555944 | 1,681307 | 0,00639 | 0,01801 |
| OCM | 0,190195 | 0,09152 | -1,05533 | 8,20E-06 | 0,000101 |
| MMP3 | 2,050745 | 6,972091 | 1,765443 | 0,013665 | 0,032757 |
| CFAP70 | 0,786549 | 0,364105 | -1,11118 | 0,00668 | 0,018676 |
| CARMIL2 | 1,345805 | 0,46144 | -1,54426 | 9,24E-08 | 3,30E-06 |
| MTUS2 | 0,820608 | 0,33275 | -1,30225 | 0,01769 | 0,040084 |
| SLC5A10 | 0,092375 | 0,042793 | -1,11012 | 7,07E-05 | 0,000537 |
| STAB2 | 0,188951 | 0,088956 | -1,08685 | 0,001772 | 0,006577 |
| STUM | 0,958196 | 0,29815 | -1,68428 | 0,002549 | 0,008771 |
| SLITRK2 | 0,181431 | 0,070903 | -1,35551 | 0,002488 | 0,008609 |
| CHGB | 272,6183 | 37,66044 | -2,85576 | 0,01769 | 0,040084 |
| MRAP | 0,317083 | 0,111337 | -1,50993 | 0,000457 | 0,00227 |
| AC010323.1 | 0,051529 | 0,024459 | -1,07505 | 0,000472 | 0,002319 |
| KCNMB2 | 1,690097 | 0,475601 | -1,82928 | 0,020864 | 0,045799 |
| REEP2 | 5,740571 | 1,472783 | -1,96265 | 0,004094 | 0,012718 |
| CXCR5 | 0,193964 | 0,044035 | -2,13907 | 0,015256 | 0,035729 |
| SEC14L4 | 0,467096 | 0,981307 | 1,070986 | 1,69E-05 | 0,000176 |
| MADCAM1 | 2,757526 | 1,057209 | -1,38311 | 1,24E-05 | 0,000139 |
| SPATA22 | 0,04175 | 0,01587 | -1,39547 | 0,004755 | 0,014263 |
| SLC12A1 | 0,02369 | 0,006953 | -1,7686 | 0,000179 | 0,001095 |
| NLRP1 | 7,267771 | 3,249156 | -1,16145 | 1,16E-07 | 3,86E-06 |
| PLAC1 | 0,086343 | 0,197537 | 1,193976 | 0,000498 | 0,002422 |
| INSM1 | 7,840984 | 1,422474 | -2,46263 | 0,005487 | 0,015942 |
| ZCCHC18 | 0,901256 | 0,309698 | -1,54108 | 2,02E-06 | 3,45E-05 |
| CCR7 | 7,449511 | 1,990722 | -1,90385 | 3,64E-05 | 0,00032 |
| GNG2 | 6,57425 | 3,079454 | -1,09415 | 0,000244 | 0,001398 |
| TTC9B | 0,325476 | 0,112228 | -1,53612 | 0,003872 | 0,01217 |
| MAS1L | 0,093773 | 0,038975 | -1,26664 | 0,018964 | 0,042483 |
| DNASE1L3 | 0,924685 | 0,331094 | -1,48172 | 1,03E-07 | 3,57E-06 |
| AC015813.2 | 0,048773 | 0,024274 | -1,00666 | 0,007369 | 0,020179 |
| GAL3ST3 | 0,04248 | 0,0177 | -1,26301 | 0,000518 | 0,002501 |
| AQP7 | 1,23204 | 0,593168 | -1,05454 | 2,66E-06 | 4,23E-05 |
| SVOP | 1,859192 | 0,359619 | -2,37014 | 0,004788 | 0,014342 |
| PRPH | 1,783554 | 0,416304 | -2,09905 | 0,001133 | 0,004618 |
| SNPH | 2,772326 | 1,231263 | -1,17096 | 1,78E-05 | 0,000183 |
| GAGE10 | 0,06796 | 0,021896 | -1,63404 | 2,23E-05 | 0,000218 |
| LYL1 | 3,091213 | 1,50355 | -1,0398 | 1,14E-08 | 7,39E-07 |
| STMN4 | 0,773648 | 0,088238 | -3,1322 | 0,002229 | 0,007887 |
| PTX3 | 6,942288 | 2,152371 | -1,68948 | 0,005488 | 0,015942 |
| OPRD1 | 0,233864 | 0,084064 | -1,47612 | 0,000244 | 0,001398 |
| GRIA3 | 1,353846 | 0,438732 | -1,62565 | 0,011975 | 0,0295 |
| IL1R2 | 5,891606 | 13,05748 | 1,148144 | 0,002313 | 0,008124 |
| ANK2 | 1,750695 | 0,706044 | -1,3101 | 6,32E-05 | 0,00049 |
| PROK1 | 0,495324 | 0,117915 | -2,07062 | 0,000465 | 0,002299 |
| SSTR3 | 3,143831 | 0,699847 | -2,16741 | 0,001918 | 0,007003 |
| IL36B | 0,030083 | 0,147217 | 2,290929 | 0,000838 | 0,003648 |
| CRYBA4 | 0,104597 | 0,047348 | -1,14345 | 6,05E-05 | 0,000474 |
| CRCT1 | 1,108034 | 3,020826 | 1,446941 | 0,005895 | 0,016898 |
| C1QTNF4 | 0,570572 | 0,214971 | -1,40827 | 2,37E-07 | 6,69E-06 |
| NPY4R2 | 0,03752 | 0,092852 | 1,30726 | 0,009409 | 0,024413 |
| TMEM89 | 0,142918 | 0,069011 | -1,05029 | 0,016701 | 0,038312 |
| CHRDL1 | 9,980772 | 3,930952 | -1,34427 | 0,000122 | 0,000817 |
| GPRASP1 | 5,189486 | 2,247703 | -1,20714 | 0,001193 | 0,004805 |
| MS4A1 | 8,346228 | 1,187102 | -2,81368 | 0,000522 | 0,002512 |
| ZSCAN1 | 0,478324 | 0,113987 | -2,06911 | 0,000309 | 0,001678 |
| RUBCNL | 1,453364 | 0,717116 | -1,01912 | 0,001022 | 0,004261 |
| CHRNA2 | 0,057732 | 0,011445 | -2,33459 | 0,007206 | 0,019854 |
| C12orf42 | 0,087443 | 0,021093 | -2,05159 | 2,11E-07 | 6,21E-06 |
| CCDC178 | 0,104347 | 0,048369 | -1,10925 | 7,16E-07 | 1,57E-05 |
| MAMLD1 | 2,857442 | 1,323268 | -1,11062 | 0,003147 | 0,010344 |
| MUCL3 | 32,27239 | 83,12588 | 1,364997 | 4,92E-06 | 6,73E-05 |
| ELAVL3 | 0,33645 | 0,046228 | -2,86356 | 0,008029 | 0,021593 |
| OGN | 9,549725 | 4,67858 | -1,02939 | 0,010561 | 0,026742 |
| ZNF831 | 0,567224 | 0,173914 | -1,70555 | 3,34E-06 | 4,98E-05 |
| PACSIN1 | 0,944956 | 0,273716 | -1,78757 | 4,35E-08 | 1,89E-06 |
| CNIH2 | 5,538753 | 1,508884 | -1,87608 | 0,016999 | 0,038824 |

**Supplementary Table 7** Four hundred and ninety-two DEGs between high- and low-risk groups in the OS-related signature.

| **Gene** | **conMean** | **treatMean** | **logFC** | **p-value** | **fdr** |
| --- | --- | --- | --- | --- | --- |
| OR2L13 | 0,054697 | 0,010244 | -2,41662 | 0,00082 | 0,008114 |
| TFAP2C | 0,83382 | 1,765971 | 1,082654 | 9,60E-05 | 0,001978 |
| S100B | 11,29895 | 5,146026 | -1,13466 | 0,001881 | 0,013905 |
| PNMA3 | 0,943617 | 0,206845 | -2,18965 | 7,06E-07 | 0,000125 |
| PABPN1L | 0,030811 | 0,013926 | -1,14561 | 0,007778 | 0,035643 |
| BPIFB4 | 0,28253 | 0,026558 | -3,41118 | 0,00502 | 0,026698 |
| TENM1 | 0,21705 | 0,087042 | -1,31825 | 0,000494 | 0,005826 |
| VWC2L | 0,0449 | 0,011099 | -2,01627 | 0,00046 | 0,00554 |
| FGF14 | 0,818081 | 0,266551 | -1,61783 | 0,000684 | 0,007138 |
| SPINK9 | 0,098921 | 0,045964 | -1,10578 | 0,012199 | 0,047858 |
| DMRT1 | 0,006203 | 0,012538 | 1,015196 | 0,004501 | 0,024868 |
| CLEC4F | 0,496204 | 0,216817 | -1,19445 | 1,67E-06 | 0,000195 |
| CACNA1A | 2,996371 | 0,507521 | -2,56168 | 0,000557 | 0,006246 |
| KCNH3 | 1,279514 | 0,367971 | -1,79793 | 0,007043 | 0,033224 |
| AFF3 | 0,69731 | 0,271311 | -1,36185 | 0,006003 | 0,0299 |
| ALLC | 0,068172 | 0,031631 | -1,10785 | 0,000716 | 0,007416 |
| EML6 | 0,289728 | 0,13949 | -1,05454 | 1,71E-05 | 0,000719 |
| NSG1 | 2,130184 | 0,79204 | -1,42733 | 0,003332 | 0,020539 |
| RAMACL | 0,138492 | 0,404987 | 1,548076 | 0,011285 | 0,045484 |
| CYP46A1 | 0,363648 | 0,087246 | -2,05939 | 0,000392 | 0,004968 |
| INSM2 | 0,054959 | 0,011231 | -2,29084 | 0,003138 | 0,019707 |
| COL26A1 | 0,690354 | 0,2343 | -1,55898 | 0,003208 | 0,019992 |
| VWA5B2 | 5,673297 | 0,995013 | -2,5114 | 0,004171 | 0,023627 |
| CACNB2 | 1,198454 | 0,491408 | -1,28618 | 0,002224 | 0,015586 |
| CFC1 | 7,137657 | 1,461276 | -2,28822 | 0,002973 | 0,018946 |
| CPA5 | 0,209887 | 0,05436 | -1,949 | 0,008582 | 0,038085 |
| DAW1 | 0,07634 | 0,276463 | 1,856579 | 0,000195 | 0,003131 |
| PPP1R1A | 9,650383 | 1,770671 | -2,44629 | 0,001133 | 0,009926 |
| SRRM4 | 0,096799 | 0,029043 | -1,73682 | 0,005933 | 0,029729 |
| NFASC | 2,47716 | 1,004023 | -1,30289 | 0,001322 | 0,010956 |
| TSPEAR | 0,308902 | 0,104767 | -1,55996 | 0,000289 | 0,004026 |
| FAM25A | 0,202015 | 0,498403 | 1,30285 | 2,22E-05 | 0,000818 |
| GPX3 | 86,39338 | 30,46098 | -1,50396 | 0,008031 | 0,036341 |
| TRPC7 | 0,112124 | 0,023868 | -2,23192 | 8,39E-06 | 0,000487 |
| C19orf81 | 0,775297 | 0,124318 | -2,64071 | 0,003486 | 0,021127 |
| KRT17 | 160,826 | 348,6312 | 1,116201 | 0,003661 | 0,021761 |
| ARG1 | 0,632914 | 0,081379 | -2,95928 | 0,000548 | 0,00619 |
| ABCC8 | 27,04035 | 5,778251 | -2,22641 | 0,00914 | 0,039629 |
| UPK2 | 0,646301 | 2,804231 | 2,117328 | 0,001754 | 0,013241 |
| AVPR1B | 0,564537 | 0,127027 | -2,15194 | 0,007069 | 0,033336 |
| MAPK8IP1 | 8,159169 | 3,600346 | -1,18029 | 0,008386 | 0,037537 |
| UPB1 | 0,112605 | 0,050859 | -1,1467 | 2,67E-05 | 0,000911 |
| CCDC183 | 1,110957 | 0,529868 | -1,0681 | 6,73E-05 | 0,001606 |
| RTN1 | 5,760201 | 1,629159 | -1,82199 | 0,002524 | 0,016959 |
| REELD1 | 0,410539 | 0,172453 | -1,25132 | 0,009775 | 0,04147 |
| RGS11 | 2,946028 | 1,143378 | -1,36547 | 1,49E-06 | 0,000182 |
| CASKIN1 | 0,221343 | 0,067093 | -1,72206 | 0,00639 | 0,031187 |
| MMD2 | 0,082471 | 0,01058 | -2,96259 | 0,002894 | 0,018616 |
| CTXN2 | 0,16665 | 0,021961 | -2,9238 | 0,00313 | 0,01968 |
| F7 | 0,514105 | 0,106597 | -2,2699 | 0,006447 | 0,031362 |
| C5orf38 | 3,016075 | 0,323955 | -3,21881 | 0,001212 | 0,010356 |
| PACRG | 1,484891 | 0,634082 | -1,22762 | 0,002973 | 0,018946 |
| ADH6 | 2,470973 | 0,981628 | -1,33183 | 0,006621 | 0,031883 |
| ZNF114 | 0,140402 | 1,094384 | 2,962482 | 0,002727 | 0,017907 |
| WDR17 | 0,562124 | 0,219462 | -1,35692 | 0,004368 | 0,024353 |
| ARMC12 | 0,46484 | 0,22872 | -1,02315 | 0,000335 | 0,004454 |
| TRIM46 | 1,971608 | 0,91572 | -1,10639 | 0,000405 | 0,00508 |
| CPA4 | 0,232681 | 0,835744 | 1,844706 | 1,58E-05 | 0,000688 |
| VWA5B1 | 0,424676 | 0,185887 | -1,19194 | 0,003591 | 0,021485 |
| RGS9 | 3,259887 | 1,141624 | -1,51374 | 0,000241 | 0,003544 |
| HS3ST4 | 0,143507 | 0,012592 | -3,51059 | 0,002093 | 0,014942 |
| BTBD17 | 0,594947 | 0,096731 | -2,6207 | 8,72E-05 | 0,001869 |
| KRT6A | 9,011251 | 65,08131 | 2,852444 | 0,001478 | 0,01183 |
| FAM205A | 0,009289 | 0,004599 | -1,01414 | 0,007589 | 0,035016 |
| TAT | 0,260903 | 0,094659 | -1,4627 | 0,001862 | 0,013816 |
| GPR6 | 0,15644 | 0,016248 | -3,26727 | 0,004063 | 0,023258 |
| SBSN | 0,06434 | 0,403867 | 2,650099 | 0,001044 | 0,009507 |
| AC009690.1 | 0,108965 | 0,052157 | -1,06293 | 8,38E-06 | 0,000487 |
| RUNDC3A | 6,59803 | 0,928595 | -2,82891 | 0,005792 | 0,029221 |
| SV2A | 4,40063 | 2,092223 | -1,07267 | 0,004701 | 0,025538 |
| LCN10 | 0,150772 | 0,065835 | -1,19543 | 0,000374 | 0,004823 |
| USP41 | 0,253419 | 0,053395 | -2,24675 | 0,001375 | 0,01128 |
| RFX6 | 2,916016 | 0,60755 | -2,26292 | 0,004246 | 0,023922 |
| KHDRBS2 | 0,313471 | 0,061655 | -2,34606 | 0,001323 | 0,010956 |
| SPINK2 | 0,485077 | 0,217513 | -1,15711 | 0,00297 | 0,018946 |
| INSYN1 | 0,868718 | 0,430084 | -1,01427 | 0,000331 | 0,00442 |
| AC008687.4 | 0,559124 | 1,130691 | 1,015965 | 0,005079 | 0,026906 |
| TPH1 | 0,659729 | 0,208904 | -1,65903 | 5,38E-05 | 0,001422 |
| KIF5A | 1,807362 | 0,57468 | -1,65305 | 0,004574 | 0,025103 |
| TCEAL5 | 3,72431 | 0,757005 | -2,2986 | 0,0038 | 0,022295 |
| KRT222 | 0,370568 | 0,185255 | -1,00023 | 0,010831 | 0,044059 |
| PTPRN2 | 34,38987 | 12,8075 | -1,42499 | 5,52E-05 | 0,00144 |
| WFIKKN1 | 0,36151 | 0,152537 | -1,24488 | 5,45E-05 | 0,001432 |
| RIMBP2 | 2,436334 | 0,464199 | -2,3919 | 0,006982 | 0,033038 |
| APOH | 21,37187 | 3,168473 | -2,75385 | 0,005792 | 0,029221 |
| AL096711.2 | 0,048798 | 0,015958 | -1,61253 | 0,010472 | 0,043276 |
| XKR4 | 0,117286 | 0,032927 | -1,83267 | 0,005316 | 0,027757 |
| BRSK1 | 2,630263 | 1,241828 | -1,08274 | 6,64E-06 | 0,000423 |
| HMGA2 | 0,489348 | 1,267937 | 1,373551 | 5,80E-07 | 0,000113 |
| ATP2A3 | 41,86064 | 18,08457 | -1,21084 | 6,07E-07 | 0,000114 |
| LGI3 | 1,095469 | 0,239092 | -2,19591 | 0,01139 | 0,04568 |
| CXCL10 | 6,410414 | 14,78279 | 1,205429 | 0,00692 | 0,032822 |
| WNT1 | 0,141089 | 0,047031 | -1,58493 | 0,006984 | 0,03304 |
| UCN3 | 26,20191 | 4,387968 | -2,57805 | 0,002128 | 0,015106 |
| SOWAHA | 2,571952 | 1,218497 | -1,07776 | 1,28E-06 | 0,000171 |
| CYP2C8 | 1,666154 | 0,800858 | -1,0569 | 0,002916 | 0,018719 |
| DDC | 10,62715 | 2,332038 | -2,18809 | 6,40E-05 | 0,001569 |
| CASP14 | 0,239708 | 3,87105 | 4,013374 | 0,006447 | 0,031362 |
| FAIM2 | 2,198086 | 1,043202 | -1,07523 | 0,000142 | 0,002582 |
| DRD2 | 0,690205 | 0,17506 | -1,97917 | 0,000203 | 0,003176 |
| MPZ | 1,956841 | 0,837335 | -1,22465 | 0,000103 | 0,002089 |
| SPTBN4 | 1,226972 | 0,290833 | -2,07684 | 0,000286 | 0,004011 |
| A1BG | 0,143963 | 0,061069 | -1,23719 | 8,50E-05 | 0,001841 |
| PCP4 | 8,301161 | 1,633668 | -2,3452 | 0,000873 | 0,008425 |
| QPCT | 35,59092 | 12,96553 | -1,45683 | 0,003836 | 0,022407 |
| APBB1 | 10,9644 | 5,453345 | -1,00761 | 1,06E-05 | 0,000541 |
| KRT13 | 3,069968 | 9,950494 | 1,696544 | 0,008204 | 0,036978 |
| ENPP2 | 17,27202 | 7,989235 | -1,11231 | 0,004966 | 0,026501 |
| MELTF | 6,785666 | 13,63575 | 1,006832 | 7,25E-05 | 0,001676 |
| ADGRG2 | 1,655638 | 0,593892 | -1,47911 | 0,003872 | 0,022519 |
| CCDC184 | 3,214898 | 1,242428 | -1,37161 | 0,000379 | 0,004851 |
| EFNB3 | 5,349201 | 1,270302 | -2,07415 | 0,005844 | 0,029374 |
| KRT9 | 0,024249 | 0,102139 | 2,074551 | 1,77E-07 | 6,90E-05 |
| TMEM61 | 3,518398 | 1,677709 | -1,06843 | 0,008171 | 0,036842 |
| IQSEC3 | 2,112322 | 0,522231 | -2,01607 | 0,001825 | 0,013626 |
| FXYD6 | 10,02479 | 4,957591 | -1,01586 | 0,000753 | 0,00768 |
| NKX2-2 | 12,45064 | 2,353256 | -2,40349 | 0,004288 | 0,024052 |
| LRRTM1 | 0,677968 | 0,301972 | -1,1668 | 0,009372 | 0,040212 |
| ADGRF3 | 0,376343 | 0,138235 | -1,44493 | 0,001844 | 0,013734 |
| CYP24A1 | 0,464573 | 1,882228 | 2,018463 | 0,007041 | 0,033224 |
| C1orf127 | 4,271283 | 1,285862 | -1,73193 | 0,011777 | 0,04675 |
| NAP1L5 | 4,575321 | 2,237571 | -1,03194 | 0,001587 | 0,012388 |
| TMPRSS6 | 3,557471 | 0,599429 | -2,56919 | 0,000452 | 0,005475 |
| MYO18B | 0,135243 | 0,036195 | -1,9017 | 0,004249 | 0,023922 |
| C14orf180 | 0,213363 | 0,076311 | -1,48334 | 0,000267 | 0,003823 |
| FAM166A | 0,077166 | 0,036711 | -1,07178 | 0,001966 | 0,014358 |
| GNAZ | 4,736841 | 1,78706 | -1,40634 | 0,001022 | 0,009375 |
| COL28A1 | 1,015596 | 0,387968 | -1,38832 | 1,90E-05 | 0,000745 |
| TSPAN7 | 12,1629 | 4,367347 | -1,47766 | 0,000634 | 0,006808 |
| PCDH10 | 0,36605 | 0,125726 | -1,54176 | 0,01168 | 0,046498 |
| ANGPTL5 | 0,10734 | 0,036572 | -1,55339 | 0,000285 | 0,004011 |
| CLEC4G | 0,395632 | 0,155396 | -1,34821 | 0,011972 | 0,047318 |
| MPPED1 | 0,6313 | 0,00738 | -6,41852 | 0,012366 | 0,048271 |
| CLUL1 | 0,338971 | 0,153519 | -1,14274 | 0,003908 | 0,02265 |
| ENTHD1 | 0,150943 | 0,32536 | 1,108035 | 0,004895 | 0,026262 |
| ZBED2 | 1,323568 | 3,632052 | 1,456352 | 7,83E-06 | 0,000481 |
| CERS1 | 1,050496 | 0,231373 | -2,18278 | 0,0038 | 0,022295 |
| AGTR1 | 1,2524 | 0,614915 | -1,02624 | 0,000761 | 0,007735 |
| ADAM29 | 0,022865 | 0,008851 | -1,36924 | 0,005372 | 0,027873 |
| PPP2R2B | 0,582512 | 0,290389 | -1,0043 | 0,001181 | 0,010152 |
| SLC22A7 | 0,046569 | 0,011627 | -2,00182 | 0,008959 | 0,039219 |
| TMEM86B | 2,550197 | 1,186858 | -1,10346 | 3,41E-05 | 0,001061 |
| F10 | 13,61281 | 3,618557 | -1,91148 | 8,71E-05 | 0,001868 |
| SLC12A5 | 0,826148 | 0,12669 | -2,70509 | 0,002753 | 0,017984 |
| DCX | 0,307968 | 0,056312 | -2,45126 | 0,004112 | 0,023449 |
| RPRM | 0,232518 | 0,095439 | -1,2847 | 6,44E-05 | 0,001573 |
| NOL4 | 1,604748 | 0,38474 | -2,06039 | 0,008605 | 0,038085 |
| SLC6A16 | 0,752917 | 0,358149 | -1,07193 | 0,00051 | 0,005917 |
| CRMP1 | 8,411256 | 3,202248 | -1,39324 | 0,001702 | 0,012968 |
| KCNF1 | 1,033184 | 0,377707 | -1,45176 | 2,20E-05 | 0,000813 |
| MANSC4 | 0,129202 | 0,050179 | -1,36448 | 0,000728 | 0,007495 |
| ALPP | 0,817715 | 2,886651 | 1,819727 | 0,00047 | 0,005629 |
| SGSM1 | 1,531062 | 0,54924 | -1,47902 | 0,000127 | 0,002401 |
| CACNA1I | 0,260646 | 0,103309 | -1,33513 | 0,00914 | 0,039629 |
| FGF17 | 0,287604 | 0,088831 | -1,69495 | 4,75E-06 | 0,000348 |
| GPLD1 | 0,287616 | 0,141642 | -1,02189 | 0,002056 | 0,014722 |
| HMGCLL1 | 0,87653 | 0,314188 | -1,48018 | 0,010561 | 0,043485 |
| DYNC1I1 | 2,206495 | 0,859571 | -1,36007 | 0,001065 | 0,009613 |
| LY6D | 10,19659 | 40,46914 | 1,988736 | 0,000473 | 0,005639 |
| PLPPR1 | 0,496831 | 0,173876 | -1,51469 | 0,003817 | 0,022387 |
| IL31RA | 0,071153 | 0,184562 | 1,375112 | 3,87E-06 | 0,000311 |
| FEV | 5,322101 | 0,691414 | -2,94437 | 0,001122 | 0,009883 |
| SYN1 | 3,59107 | 1,531351 | -1,22961 | 0,000588 | 0,006482 |
| TGM4 | 0,013331 | 0,100343 | 2,912036 | 0,000351 | 0,004577 |
| STXBP5L | 0,320075 | 0,068763 | -2,21872 | 0,009179 | 0,039774 |
| CDO1 | 5,197 | 2,122132 | -1,29216 | 0,000837 | 0,0082 |
| KLHL41 | 1,163875 | 0,330408 | -1,81661 | 0,003059 | 0,019316 |
| TCEAL2 | 8,575701 | 1,475177 | -2,53937 | 0,004723 | 0,025646 |
| NR0B1 | 1,665285 | 0,383203 | -2,11959 | 0,009173 | 0,039761 |
| ARHGAP19-SLIT1 | 0,029096 | 0,00642 | -2,18016 | 0,006073 | 0,030115 |
| NACAD | 3,028002 | 1,115785 | -1,44031 | 0,001789 | 0,013411 |
| P2RX6 | 0,43288 | 0,150639 | -1,52287 | 0,001076 | 0,009672 |
| C8orf86 | 0,017871 | 0,004652 | -1,9416 | 0,001436 | 0,011619 |
| CCER2 | 0,472051 | 0,223717 | -1,07727 | 0,000379 | 0,004851 |
| OR52N4 | 0,215808 | 0,087767 | -1,298 | 0,00276 | 0,018021 |
| C4orf50 | 0,0378 | 0,016949 | -1,15713 | 0,000662 | 0,007011 |
| ADAMTS18 | 0,298843 | 0,116433 | -1,35989 | 0,000255 | 0,003698 |
| ALK | 0,365205 | 0,046056 | -2,98726 | 0,000669 | 0,007051 |
| DKK1 | 6,543938 | 17,06071 | 1,382447 | 5,05E-05 | 0,00136 |
| KCNH2 | 6,388702 | 2,214021 | -1,52885 | 0,002834 | 0,018332 |
| TOGARAM2 | 0,219624 | 0,085824 | -1,35559 | 0,000761 | 0,007735 |
| PHF21B | 0,348084 | 0,079691 | -2,12695 | 0,000489 | 0,00579 |
| REC8 | 7,217313 | 3,396097 | -1,08758 | 1,64E-05 | 0,000705 |
| DACH2 | 0,225715 | 0,062456 | -1,85359 | 0,010644 | 0,04355 |
| TNNI3K | 0,058001 | 0,026928 | -1,10696 | 0,004532 | 0,024956 |
| PTGES | 16,3277 | 34,59567 | 1,08327 | 2,57E-05 | 0,00089 |
| BSN | 0,536715 | 0,159252 | -1,75284 | 0,010741 | 0,043834 |
| KCNH6 | 4,334201 | 0,425993 | -3,34686 | 0,005197 | 0,027252 |
| SCN3B | 0,87899 | 0,302191 | -1,54039 | 0,002807 | 0,018204 |
| SYP | 9,818482 | 1,598274 | -2,61899 | 0,003626 | 0,021644 |
| PDZD4 | 4,366736 | 1,422352 | -1,61828 | 6,56E-05 | 0,001582 |
| SYT3 | 0,369261 | 0,152131 | -1,27933 | 0,000855 | 0,00832 |
| OGDHL | 2,618788 | 0,780554 | -1,74633 | 0,011876 | 0,047027 |
| PRKCG | 1,617764 | 0,704878 | -1,19856 | 0,004574 | 0,025103 |
| LRRC4B | 2,996372 | 0,525778 | -2,51069 | 0,001349 | 0,011108 |
| VWA7 | 4,800936 | 2,28174 | -1,07318 | 0,008031 | 0,036341 |
| MSTN | 0,145132 | 0,072443 | -1,00245 | 6,89E-05 | 0,00162 |
| TTLL6 | 1,187767 | 0,375656 | -1,66077 | 0,000794 | 0,007946 |
| IRX2 | 6,833086 | 0,52306 | -3,70749 | 0,006799 | 0,032393 |
| SNTG1 | 0,451073 | 0,049468 | -3,1888 | 0,012465 | 0,048545 |
| ATP1B2 | 2,552851 | 0,843054 | -1,59841 | 0,000249 | 0,003636 |
| SNAP25 | 10,35054 | 2,619056 | -1,98259 | 0,005341 | 0,027757 |
| FAM135B | 0,362417 | 0,07055 | -2,36094 | 0,008495 | 0,037842 |
| C21orf58 | 2,372824 | 1,00342 | -1,24168 | 0,000149 | 0,002648 |
| AIRE | 0,091172 | 0,040299 | -1,17784 | 7,36E-06 | 0,000465 |
| ANXA8 | 1,301926 | 3,999277 | 1,619092 | 1,14E-05 | 0,000567 |
| CCDC188 | 1,048875 | 0,328341 | -1,67558 | 8,60E-05 | 0,001853 |
| GPR87 | 2,393294 | 8,53958 | 1,835167 | 1,98E-05 | 0,000761 |
| PRLR | 1,52557 | 0,35443 | -2,10578 | 0,00051 | 0,005917 |
| ZDHHC11B | 1,268099 | 0,534551 | -1,24627 | 1,48E-05 | 0,000667 |
| GSDMC | 0,35941 | 1,12933 | 1,651765 | 0,000366 | 0,004732 |
| SSTR1 | 4,433164 | 2,084057 | -1,08894 | 0,001011 | 0,009307 |
| ANKS1B | 0,290648 | 0,080393 | -1,85414 | 0,002224 | 0,015586 |
| PTPRT | 0,88987 | 0,139089 | -2,67759 | 0,001862 | 0,013816 |
| IL20RB | 1,886448 | 8,666161 | 2,199721 | 0,000387 | 0,004934 |
| ATP1A2 | 1,355828 | 0,547624 | -1,30792 | 0,011583 | 0,046274 |
| CRB2 | 0,078511 | 0,033882 | -1,21237 | 0,011196 | 0,045203 |
| DUSP26 | 7,536362 | 1,777226 | -2,08424 | 0,005058 | 0,026804 |
| KCNK17 | 3,116965 | 0,988403 | -1,65697 | 0,00346 | 0,02104 |
| FAM218A | 0,411067 | 0,202178 | -1,02375 | 0,002076 | 0,014839 |
| SSTR2 | 4,102385 | 0,546912 | -2,90708 | 0,006563 | 0,031684 |
| AC011473.4 | 0,012953 | 0,055236 | 2,092271 | 0,00541 | 0,02797 |
| SCAMP5 | 11,91652 | 4,993336 | -1,25489 | 6,73E-05 | 0,001606 |
| ADCY2 | 0,542442 | 0,227893 | -1,25111 | 0,00346 | 0,02104 |
| FRRS1L | 0,953656 | 0,246614 | -1,95121 | 0,004038 | 0,023191 |
| TMEM130 | 3,653836 | 1,772634 | -1,04352 | 0,002598 | 0,017317 |
| FAM163A | 1,688746 | 0,349236 | -2,27368 | 0,004659 | 0,02537 |
| SCGB2A1 | 20,71932 | 7,219683 | -1,52097 | 0,003836 | 0,022407 |
| CHST8 | 0,985084 | 0,317461 | -1,63367 | 0,004449 | 0,024667 |
| LONRF2 | 1,805671 | 0,862545 | -1,06586 | 1,38E-06 | 0,000179 |
| KNDC1 | 1,190914 | 0,501654 | -1,2473 | 0,002056 | 0,014722 |
| GPR162 | 3,06055 | 1,249349 | -1,29261 | 4,01E-06 | 0,000314 |
| GADD45G | 17,95361 | 8,191443 | -1,13208 | 8,38E-06 | 0,000487 |
| PAGE2B | 0,242083 | 0,058514 | -2,04864 | 0,009493 | 0,040612 |
| BEX2 | 18,46245 | 7,218626 | -1,3548 | 0,003238 | 0,020123 |
| FAM71F1 | 0,045832 | 0,019931 | -1,20133 | 0,000359 | 0,004673 |
| CRIP3 | 0,698894 | 0,251422 | -1,47497 | 3,90E-06 | 0,000311 |
| KRT16 | 21,84596 | 58,91124 | 1,431176 | 0,004288 | 0,024052 |
| ATP1A3 | 3,666028 | 0,568806 | -2,68821 | 0,00082 | 0,008114 |
| KIAA0408 | 0,024436 | 0,008781 | -1,47654 | 0,001617 | 0,012547 |
| RHOXF1 | 0,364865 | 0,149367 | -1,2885 | 0,001177 | 0,010152 |
| CFAP65 | 0,251907 | 0,111512 | -1,17569 | 0,001099 | 0,009767 |
| BRINP1 | 0,934067 | 0,286685 | -1,70406 | 0,006447 | 0,031362 |
| PIPOX | 2,052519 | 0,49788 | -2,04353 | 0,004056 | 0,023227 |
| ASPDH | 2,142409 | 0,400763 | -2,41841 | 0,000261 | 0,003762 |
| C22orf42 | 1,536302 | 0,198076 | -2,95534 | 0,006947 | 0,032936 |
| EFCAB1 | 0,090576 | 0,194693 | 1,104007 | 0,007892 | 0,035968 |
| RGS20 | 0,133761 | 0,2993 | 1,161935 | 0,000551 | 0,006193 |
| SLC16A11 | 1,701798 | 0,74359 | -1,19448 | 0,000428 | 0,005285 |
| DLGAP1 | 0,209871 | 0,094943 | -1,14436 | 0,008985 | 0,039219 |
| KIAA0319 | 0,967843 | 0,418106 | -1,21091 | 0,008459 | 0,037704 |
| EREG | 1,006061 | 3,483726 | 1,791913 | 0,000121 | 0,002328 |
| RP1 | 0,029068 | 0,064793 | 1,156403 | 0,000202 | 0,003176 |
| LY6H | 6,777821 | 0,698637 | -3,27821 | 0,000127 | 0,002401 |
| ACTL6B | 2,387454 | 0,279717 | -3,09343 | 0,010552 | 0,043485 |
| MYT1 | 0,977355 | 0,147592 | -2,72727 | 0,005197 | 0,027252 |
| S100A2 | 13,01375 | 108,6346 | 3,061375 | 0,000157 | 0,002702 |
| UCHL1 | 21,55134 | 9,751885 | -1,14402 | 0,004966 | 0,026501 |
| PLIN1 | 3,408011 | 1,039354 | -1,71324 | 0,000457 | 0,005509 |
| PRRT3 | 2,378421 | 1,044384 | -1,18735 | 0,000706 | 0,00733 |
| SMIM32 | 10,54993 | 1,177671 | -3,16323 | 9,61E-06 | 0,000515 |
| TNNT1 | 2,380239 | 6,954111 | 1,54676 | 0,001181 | 0,010152 |
| KRT78 | 0,048175 | 0,128249 | 1,412589 | 0,000963 | 0,009025 |
| FAM47C | 0,014855 | 0,003353 | -2,14745 | 0,007584 | 0,035002 |
| LINGO3 | 0,602626 | 0,184943 | -1,70418 | 0,001099 | 0,009767 |
| WNT10B | 0,629544 | 0,227847 | -1,46624 | 0,000634 | 0,006808 |
| AC113554.1 | 0,021902 | 0,003596 | -2,60641 | 8,29E-05 | 0,001818 |
| NECAB2 | 2,769955 | 0,729921 | -1,92405 | 0,001937 | 0,014218 |
| TBC1D10C | 3,416325 | 1,616 | -1,08002 | 0,005792 | 0,029221 |
| CHGA | 193,9889 | 35,80043 | -2,43792 | 0,000873 | 0,008425 |
| NAP1L2 | 2,30697 | 0,94368 | -1,28963 | 0,001539 | 0,012147 |
| ZNF540 | 1,249688 | 0,555157 | -1,1706 | 4,00E-08 | 3,06E-05 |
| HFM1 | 0,123912 | 0,058075 | -1,09334 | 0,003492 | 0,021127 |
| AC004805.1 | 0,015717 | 0,006611 | -1,24943 | 1,44E-05 | 0,000657 |
| WNT4 | 11,91887 | 3,16976 | -1,9108 | 0,011777 | 0,04675 |
| CCDC187 | 0,742318 | 0,276804 | -1,42317 | 0,001685 | 0,012898 |
| SLC22A10 | 0,041363 | 0,007652 | -2,43445 | 0,012618 | 0,049011 |
| COL2A1 | 0,231544 | 0,025994 | -3,15504 | 0,007421 | 0,034462 |
| CEACAM19 | 2,943237 | 1,345503 | -1,12926 | 0,005197 | 0,027252 |
| KIF12 | 33,60771 | 15,98661 | -1,07193 | 0,001011 | 0,009307 |
| SAXO2 | 0,52531 | 0,236075 | -1,15392 | 0,001825 | 0,013626 |
| HES2 | 0,37957 | 1,095424 | 1,529051 | 0,000157 | 0,002702 |
| GPC5 | 0,252636 | 0,115774 | -1,12575 | 9,74E-06 | 0,000519 |
| DPY19L2 | 0,900802 | 0,418902 | -1,1046 | 0,000306 | 0,004198 |
| KSR2 | 0,350692 | 0,15048 | -1,22063 | 0,007556 | 0,034884 |
| RAB26 | 6,792708 | 2,935969 | -1,21015 | 0,000837 | 0,0082 |
| TUNAR | 2,389327 | 0,767767 | -1,63786 | 0,007961 | 0,036182 |
| SBK2 | 0,671588 | 0,116007 | -2,53337 | 0,002439 | 0,016578 |
| DLGAP2 | 0,064494 | 0,019449 | -1,72944 | 0,009258 | 0,039987 |
| PSD | 5,486419 | 2,473909 | -1,14907 | 7,33E-05 | 0,001691 |
| SLC22A17 | 25,06476 | 6,23743 | -2,00664 | 2,89E-05 | 0,000958 |
| RTN4RL1 | 2,352871 | 1,145774 | -1,0381 | 0,010651 | 0,04355 |
| FXYD7 | 0,327355 | 0,134926 | -1,27869 | 0,006112 | 0,030196 |
| JAKMIP2 | 0,908386 | 0,353772 | -1,36048 | 4,34E-05 | 0,001241 |
| SPTB | 0,65429 | 0,267645 | -1,28961 | 0,006982 | 0,033038 |
| PNMA8C | 1,157733 | 0,175517 | -2,72162 | 0,005336 | 0,027757 |
| DCBLD2 | 5,943091 | 12,91198 | 1,119425 | 2,40E-06 | 0,000231 |
| PRODH2 | 0,633303 | 0,043442 | -3,86574 | 0,001365 | 0,011208 |
| C2orf66 | 0,13354 | 0,066672 | -1,00211 | 0,00075 | 0,007667 |
| NDRG4 | 2,62518 | 1,257816 | -1,0615 | 0,005537 | 0,02838 |
| DIRAS2 | 0,837558 | 0,334114 | -1,32585 | 0,003059 | 0,019316 |
| CITED1 | 1,046141 | 0,465527 | -1,16814 | 0,001043 | 0,009502 |
| CRHBP | 0,285978 | 0,10203 | -1,48692 | 0,000901 | 0,008633 |
| ANLN | 3,032075 | 6,770522 | 1,158961 | 5,21E-10 | 3,86E-06 |
| FAM83A | 3,367291 | 13,94881 | 2,050482 | 7,51E-06 | 0,000469 |
| ZNF483 | 0,493845 | 0,187182 | -1,39962 | 0,001032 | 0,009438 |
| MLXIPL | 9,048451 | 3,628643 | -1,31824 | 0,001157 | 0,010021 |
| MAPK8IP2 | 3,738585 | 1,097411 | -1,76839 | 0,001076 | 0,009672 |
| C16orf74 | 1,201845 | 3,176307 | 1,402099 | 0,001555 | 0,012231 |
| GPR142 | 1,730037 | 0,272816 | -2,6648 | 0,001825 | 0,013626 |
| RIC3 | 1,246335 | 0,456042 | -1,45045 | 0,000182 | 0,002997 |
| PKP1 | 2,105078 | 4,905047 | 1,220393 | 0,012277 | 0,04805 |
| KCNJ11 | 4,742065 | 1,628218 | -1,54222 | 0,000505 | 0,005866 |
| AC007906.2 | 3,508454 | 7,04901 | 1,006585 | 0,002097 | 0,014942 |
| KCNA5 | 3,730287 | 0,707726 | -2,39802 | 0,002834 | 0,018332 |
| PCSK1N | 139,7917 | 23,01023 | -2,60293 | 0,000846 | 0,008276 |
| DDX25 | 0,272892 | 0,064294 | -2,08556 | 0,006563 | 0,031684 |
| RAB3C | 1,532482 | 0,625117 | -1,29367 | 0,003872 | 0,022519 |
| TNMD | 0,253439 | 0,071901 | -1,81754 | 0,000427 | 0,005279 |
| PROZ | 0,234356 | 0,102404 | -1,19442 | 0,002739 | 0,017977 |
| GNB3 | 0,846784 | 0,408499 | -1,05166 | 7,17E-07 | 0,000125 |
| ASIC4 | 0,130919 | 0,0502 | -1,38292 | 0,005487 | 0,028223 |
| TMEM151B | 0,191499 | 0,062383 | -1,61812 | 0,005663 | 0,028847 |
| CACNA2D2 | 5,573425 | 1,453892 | -1,93864 | 0,002224 | 0,015586 |
| GIPR | 7,36343 | 2,516011 | -1,54924 | 9,74E-06 | 0,000519 |
| FYB2 | 0,733618 | 0,180795 | -2,02068 | 0,012901 | 0,049796 |
| KLKB1 | 1,433756 | 0,632255 | -1,18122 | 0,011391 | 0,04568 |
| VGF | 16,05095 | 3,791627 | -2,08177 | 0,009219 | 0,03983 |
| S100Z | 0,33045 | 0,128866 | -1,35856 | 6,56E-05 | 0,001582 |
| DLGAP3 | 0,67237 | 0,228104 | -1,55957 | 0,000139 | 0,002556 |
| SYNGR3 | 1,659901 | 0,828504 | -1,00252 | 0,000185 | 0,003015 |
| LPL | 7,57872 | 3,269986 | -1,21267 | 0,000669 | 0,007051 |
| CELF3 | 7,090506 | 1,166446 | -2,60377 | 0,004877 | 0,026173 |
| NPAP1 | 0,009898 | 0,003889 | -1,34767 | 0,007309 | 0,034105 |
| ATP6V0E2 | 14,77805 | 7,381219 | -1,00153 | 9,37E-05 | 0,001947 |
| SCEL | 3,957061 | 8,533757 | 1,108752 | 7,70E-05 | 0,00173 |
| NPAS4 | 0,162034 | 0,053012 | -1,61191 | 0,002523 | 0,016959 |
| SRCIN1 | 1,502198 | 0,680856 | -1,14165 | 0,000699 | 0,007267 |
| PCDHA2 | 0,077797 | 0,02962 | -1,39317 | 0,006305 | 0,030942 |
| KIF19 | 1,375137 | 0,26431 | -2,37927 | 0,004449 | 0,024667 |
| NYAP1 | 1,755078 | 0,681923 | -1,36385 | 0,002727 | 0,017907 |
| OLFM1 | 5,829949 | 1,955469 | -1,57597 | 0,003332 | 0,020539 |
| PTGDR2 | 1,107742 | 0,501218 | -1,14411 | 0,002335 | 0,016137 |
| P2RX2 | 0,246322 | 0,101968 | -1,27243 | 0,000798 | 0,007982 |
| NOVA1 | 0,778675 | 0,25263 | -1,62399 | 0,007425 | 0,034462 |
| FBLL1 | 3,376076 | 0,758356 | -2,1544 | 0,004449 | 0,024667 |
| NEURL1 | 7,29734 | 2,830812 | -1,36615 | 0,004616 | 0,025231 |
| DUSP15 | 0,792178 | 0,313324 | -1,33817 | 0,001322 | 0,010956 |
| AC008758.6 | 0,127952 | 0,062766 | -1,02755 | 8,32E-05 | 0,001821 |
| CROCC2 | 0,073557 | 0,036233 | -1,02158 | 0,004743 | 0,025679 |
| RAB3A | 4,621793 | 1,621137 | -1,51145 | 5,52E-05 | 0,00144 |
| WNK4 | 2,222997 | 0,664236 | -1,74274 | 0,000828 | 0,008168 |
| JPH3 | 0,905206 | 0,265123 | -1,77158 | 0,001524 | 0,012057 |
| PCDHA1 | 0,115774 | 0,040186 | -1,52653 | 0,004166 | 0,023627 |
| SNX22 | 1,057528 | 0,528131 | -1,00173 | 0,001862 | 0,013816 |
| KRT73 | 0,033922 | 0,008044 | -2,07634 | 0,003754 | 0,022149 |
| FGFBP1 | 5,893784 | 12,15324 | 1,044075 | 0,000769 | 0,007802 |
| PLEKHD1 | 0,081575 | 0,039318 | -1,05296 | 0,000193 | 0,003108 |
| CHD5 | 0,570235 | 0,200956 | -1,50468 | 0,008031 | 0,036341 |
| PRB3 | 0,072173 | 0,156671 | 1,118214 | 0,001237 | 0,010512 |
| CTSV | 1,048373 | 2,49957 | 1,253529 | 1,76E-05 | 0,00072 |
| FBXL16 | 3,638536 | 1,188345 | -1,6144 | 0,000627 | 0,00677 |
| MS4A8 | 11,02309 | 4,631642 | -1,25093 | 0,000164 | 0,002803 |
| TAL2 | 0,137667 | 0,06304 | -1,12684 | 0,001754 | 0,013241 |
| GRIK5 | 2,618072 | 0,806572 | -1,69863 | 0,004368 | 0,024353 |
| REM2 | 0,8102 | 0,320303 | -1,33884 | 1,96E-07 | 6,90E-05 |
| MET | 15,10284 | 30,59977 | 1,018701 | 2,19E-10 | 3,24E-06 |
| MSI1 | 3,112808 | 0,878265 | -1,82549 | 0,003395 | 0,020835 |
| JAKMIP1 | 1,014202 | 0,357223 | -1,50545 | 0,000218 | 0,003319 |
| PHF24 | 0,172861 | 0,078178 | -1,14478 | 0,004532 | 0,024956 |
| GPD1 | 6,729493 | 2,954559 | -1,18755 | 0,001996 | 0,014418 |
| SLC4A8 | 0,779211 | 0,260773 | -1,57922 | 0,000151 | 0,002655 |
| EFCAB8 | 0,067863 | 0,029299 | -1,21177 | 0,000137 | 0,002538 |
| ACSL6 | 0,347375 | 0,099072 | -1,80994 | 0,004532 | 0,024956 |
| CXCL11 | 1,701569 | 4,145947 | 1,284836 | 0,011975 | 0,047318 |
| PEMT | 22,16781 | 8,696191 | -1,35001 | 0,001076 | 0,009672 |
| SMIM23 | 0,089629 | 0,040996 | -1,12848 | 0,001991 | 0,014418 |
| ADGRG5 | 2,791201 | 0,962324 | -1,53629 | 0,001309 | 0,010894 |
| A2ML1 | 0,483649 | 3,563693 | 2,88134 | 0,000607 | 0,006621 |
| NCAM1 | 3,125745 | 1,024702 | -1,609 | 0,00303 | 0,01919 |
| FNDC5 | 0,891519 | 0,404711 | -1,13937 | 0,004094 | 0,023353 |
| CRYBA2 | 28,33176 | 3,487639 | -3,0221 | 0,00373 | 0,022022 |
| ASB16 | 1,148779 | 0,53846 | -1,09319 | 1,48E-05 | 0,000667 |
| SLC22A16 | 0,132469 | 0,051492 | -1,36323 | 6,90E-05 | 0,00162 |
| GP1BA | 1,026587 | 0,496228 | -1,04878 | 0,005389 | 0,027873 |
| GDAP1L1 | 0,705601 | 0,150402 | -2,23003 | 0,000837 | 0,0082 |
| MAP6 | 3,59163 | 1,329406 | -1,43386 | 0,000379 | 0,004851 |
| ZNF157 | 0,089588 | 0,042492 | -1,07612 | 0,002361 | 0,016264 |
| PCDH15 | 0,034399 | 0,003983 | -3,11039 | 0,00168 | 0,012898 |
| PCARE | 0,024365 | 0,008451 | -1,52767 | 0,000138 | 0,002552 |
| TMEM179 | 5,750413 | 0,631323 | -3,18722 | 0,00274 | 0,017977 |
| ELANE | 6,827653 | 0,20073 | -5,08806 | 0,005486 | 0,028223 |
| ACMSD | 1,314906 | 0,449982 | -1,54702 | 0,002861 | 0,018444 |
| SCN3A | 0,339384 | 0,137196 | -1,30668 | 0,00142 | 0,011521 |
| ZAP70 | 2,360504 | 1,119046 | -1,07683 | 0,001881 | 0,013905 |
| USP27X | 3,827311 | 1,745052 | -1,13306 | 0,00035 | 0,004568 |
| TMEM132E | 0,884223 | 0,293366 | -1,59171 | 0,000662 | 0,007011 |
| GHRHR | 0,342291 | 0,067246 | -2,34771 | 0,003268 | 0,020272 |
| RASGRP2 | 3,536412 | 1,273554 | -1,47343 | 0,00095 | 0,008942 |
| TRIM67 | 0,093642 | 0,039585 | -1,24221 | 0,000489 | 0,00579 |
| TMEM272 | 0,066569 | 0,028761 | -1,21076 | 6,55E-06 | 0,000423 |
| RGS7 | 1,036888 | 0,446437 | -1,21573 | 1,09E-05 | 0,00055 |
| GRM4 | 0,384093 | 0,120459 | -1,67292 | 0,004944 | 0,026456 |
| SCG2 | 88,69104 | 17,95102 | -2,30472 | 0,008607 | 0,038085 |
| ANXA8L1 | 0,481874 | 1,403176 | 1,541969 | 1,90E-05 | 0,000745 |
| TMEM178B | 1,574612 | 0,53625 | -1,55402 | 0,004328 | 0,024175 |
| TP63 | 0,424127 | 1,172865 | 1,467468 | 0,003945 | 0,022764 |
| NRG2 | 0,421619 | 0,207745 | -1,02112 | 0,003427 | 0,020955 |
| ASTN1 | 0,546409 | 0,148592 | -1,87862 | 0,012277 | 0,04805 |
| RTL5 | 4,701122 | 1,980226 | -1,24734 | 0,000677 | 0,007082 |
| SLC6A13 | 0,07631 | 0,027531 | -1,47079 | 0,008627 | 0,038165 |
| RAB39B | 1,441654 | 0,409951 | -1,8142 | 0,00092 | 0,008777 |
| STPG3 | 0,310643 | 0,154896 | -1,00396 | 1,85E-05 | 0,000739 |
| JAKMIP3 | 0,226618 | 0,107799 | -1,07191 | 6,85E-07 | 0,000125 |
| ERICH3 | 0,147828 | 0,068367 | -1,11255 | 0,007197 | 0,033746 |
| SERPINF2 | 6,450145 | 3,146779 | -1,03546 | 0,000331 | 0,00442 |
| ANKRD18B | 0,039461 | 0,104153 | 1,400206 | 0,000285 | 0,004011 |
| B3GAT1 | 0,586489 | 0,12221 | -2,26275 | 0,000331 | 0,00442 |
| SNAP91 | 1,321195 | 0,337825 | -1,9675 | 0,001282 | 0,010783 |
| CACNA1B | 0,72712 | 0,095609 | -2,92698 | 0,006534 | 0,031684 |
| HSD17B3 | 0,630805 | 0,27145 | -1,21651 | 0,00021 | 0,003231 |
| KRT83 | 0,035923 | 0,075531 | 1,072139 | 0,00034 | 0,004491 |
| PRB1 | 0,053684 | 0,322751 | 2,587864 | 0,012352 | 0,048271 |
| CARMIL3 | 0,681395 | 0,276503 | -1,30119 | 2,59E-08 | 2,56E-05 |
| CDK5R2 | 4,707144 | 0,929237 | -2,34073 | 0,008386 | 0,037537 |
| CADPS | 2,711878 | 1,011534 | -1,42275 | 0,000669 | 0,007051 |
| BRINP2 | 0,201455 | 0,045542 | -2,1452 | 0,000526 | 0,006043 |
| TGFB2 | 3,018763 | 6,092102 | 1,012983 | 0,000433 | 0,005304 |
| ICA1L | 0,871305 | 0,413186 | -1,07638 | 0,000104 | 0,002106 |
| SERTM2 | 0,066347 | 0,025083 | -1,40332 | 0,00105 | 0,009548 |
| CPLX1 | 3,822575 | 1,694906 | -1,17334 | 5,87E-05 | 0,001483 |
| KL | 3,477555 | 0,528891 | -2,71703 | 0,006447 | 0,031362 |
| CALY | 14,13273 | 1,15295 | -3,61564 | 0,000598 | 0,006558 |
| RASA4 | 0,207092 | 0,100205 | -1,04731 | 2,06E-05 | 0,000779 |
| TEX38 | 0,121463 | 0,053248 | -1,18971 | 0,000219 | 0,003325 |
| SLC8A3 | 0,216453 | 0,069017 | -1,64903 | 0,006334 | 0,030972 |
| DNAI2 | 0,22519 | 0,028572 | -2,97844 | 0,00428 | 0,024052 |
| RND2 | 1,048392 | 0,338539 | -1,63078 | 0,000225 | 0,003378 |
| RXRG | 1,510435 | 0,522799 | -1,53064 | 0,010651 | 0,04355 |
| CCDC181 | 1,199544 | 0,556636 | -1,10768 | 8,09E-05 | 0,001796 |
| TMEM40 | 0,254959 | 1,026792 | 2,009808 | 0,000527 | 0,006043 |
| GNAO1 | 3,094369 | 1,159994 | -1,41553 | 0,001957 | 0,014303 |
| KRTAP5-5 | 0,268901 | 0,083424 | -1,68854 | 0,00032 | 0,004324 |
| CHRNB2 | 0,928401 | 0,223734 | -2,05296 | 0,00749 | 0,034656 |
| TMEM74B | 4,133404 | 1,302906 | -1,6656 | 2,81E-05 | 0,00094 |
| CAMK2B | 2,885004 | 0,576051 | -2,32431 | 0,012796 | 0,049478 |
| SLC35F4 | 0,207131 | 0,048101 | -2,1064 | 0,007554 | 0,034884 |
| SSUH2 | 0,356278 | 0,143042 | -1,31657 | 0,007689 | 0,035322 |
| SCG5 | 131,4927 | 33,06814 | -1,99147 | 0,00686 | 0,032627 |
| CXXC4 | 2,191742 | 0,678181 | -1,69234 | 0,001231 | 0,010469 |
| AP3B2 | 1,070016 | 0,309575 | -1,78927 | 0,008533 | 0,037894 |
| FFAR3 | 0,139684 | 0,065838 | -1,08518 | 0,000153 | 0,002673 |
| ASIC3 | 1,151624 | 0,550137 | -1,06581 | 0,000191 | 0,003085 |
| CARMIL2 | 1,229125 | 0,579445 | -1,08489 | 0,003908 | 0,02265 |
| MRAP | 0,299764 | 0,128853 | -1,2181 | 0,000576 | 0,006386 |
| KCNMB2 | 1,722201 | 0,443132 | -1,95844 | 0,005341 | 0,027757 |
| REEP2 | 5,75574 | 1,457442 | -1,98156 | 0,004409 | 0,024514 |
| AC119396.1 | 1,131782 | 0,460619 | -1,29695 | 0,001157 | 0,010021 |
| AGBL4 | 0,125852 | 0,039117 | -1,68587 | 0,001043 | 0,009502 |
| SLC12A1 | 0,023714 | 0,006929 | -1,77511 | 0,00074 | 0,00758 |
| NLRP1 | 7,314482 | 3,201914 | -1,19182 | 6,25E-08 | 3,62E-05 |
| PLAC1 | 0,068722 | 0,215358 | 1,647895 | 1,28E-05 | 0,000608 |
| MAP7D2 | 1,068859 | 0,428372 | -1,31914 | 0,008459 | 0,037704 |
| INSM1 | 7,874442 | 1,388636 | -2,50351 | 0,004075 | 0,023316 |
| ZCCHC18 | 0,902909 | 0,308026 | -1,55153 | 1,99E-06 | 0,00021 |
| GNG2 | 6,509691 | 3,144747 | -1,04964 | 0,010832 | 0,044059 |
| FAM183A | 0,701731 | 0,303474 | -1,20935 | 0,003544 | 0,021353 |
| TMEM151A | 2,345191 | 0,970345 | -1,27314 | 0,008607 | 0,038085 |
| SMOC2 | 34,58419 | 15,81699 | -1,12864 | 0,000296 | 0,004088 |
| AQP7 | 1,219063 | 0,606293 | -1,00769 | 1,49E-06 | 0,000182 |
| SLC6A19 | 2,227941 | 1,056583 | -1,07631 | 0,012377 | 0,048271 |
| SNPH | 2,749174 | 1,254677 | -1,13168 | 4,24E-05 | 0,001222 |
| GAGE10 | 0,060339 | 0,029603 | -1,02734 | 0,003555 | 0,021353 |
| PDE3B | 1,870352 | 0,911 | -1,03779 | 1,52E-05 | 0,000673 |
| IL11 | 1,631628 | 3,495135 | 1,099036 | 0,011777 | 0,04675 |
| HCN1 | 0,130759 | 0,049314 | -1,40685 | 0,00483 | 0,02602 |
| OPRD1 | 0,237019 | 0,080874 | -1,55125 | 0,004701 | 0,025538 |
| FNDC9 | 0,059797 | 0,028575 | -1,0653 | 0,003677 | 0,021848 |
| GRIA3 | 1,370655 | 0,421733 | -1,70046 | 0,01021 | 0,042609 |
| ANK2 | 1,72202 | 0,735045 | -1,2282 | 0,012902 | 0,049796 |
| PROK1 | 0,497462 | 0,115753 | -2,10354 | 0,000179 | 0,002979 |
| DNASE2B | 2,300842 | 0,237144 | -3,27832 | 0,002943 | 0,018817 |
| SSTR3 | 3,178528 | 0,664756 | -2,25746 | 0,001603 | 0,012486 |
| SLC7A8 | 18,15564 | 8,405432 | -1,11102 | 0,00093 | 0,00883 |
| NPY4R2 | 0,035319 | 0,095079 | 1,428703 | 0,005278 | 0,027595 |
| SH2D5 | 0,057157 | 0,148322 | 1,375725 | 6,48E-05 | 0,001573 |
| GPRASP1 | 5,387636 | 2,047301 | -1,39593 | 3,54E-05 | 0,001093 |
| KRT14 | 2,118374 | 12,87813 | 2,603894 | 0,008532 | 0,037894 |
| ZSCAN1 | 0,466182 | 0,126267 | -1,88442 | 0,005638 | 0,028727 |
| ZNF831 | 0,535952 | 0,205541 | -1,38267 | 0,003059 | 0,019316 |
| PACSIN1 | 0,891013 | 0,328272 | -1,44055 | 0,009458 | 0,040486 |
| CNIH2 | 5,50995 | 1,538014 | -1,84097 | 0,008533 | 0,037894 |

**Supplementary Table 8** Forty-eight DETFs between PAAD and normal pancreatic tissues.

| **Gene** | **conMean** | **treatMean** | **logFC** | **p-value** | **fdr** |
| --- | --- | --- | --- | --- | --- |
| NCAPG | 0,223124 | 1,305935 | 2,549164 | 8,32E-49 | 2,75E-48 |
| PAX5 | 0,049314 | 0,474537 | 3,266447 | 5,05E-15 | 6,79E-15 |
| NR5A2 | 4,229833 | 1,860926 | -1,18458 | 1,40E-51 | 6,09E-51 |
| EOMES | 0,222587 | 0,49268 | 1,14628 | 1,66E-07 | 1,99E-07 |
| SPDEF | 0,322429 | 3,49531 | 3,438366 | 6,48E-49 | 2,19E-48 |
| BCL11A | 1,77298 | 0,683806 | -1,37452 | 6,25E-48 | 1,99E-47 |
| TP63 | 0,103838 | 0,590944 | 2,508685 | 5,10E-28 | 8,36E-28 |
| SOX2 | 0,130232 | 0,57719 | 2,147963 | 3,44E-21 | 5,18E-21 |
| CENPA | 0,194431 | 1,274558 | 2,71267 | 9,00E-50 | 3,33E-49 |
| HOXB13 | 0,019906 | 0,498648 | 4,64674 | 1,80E-23 | 2,79E-23 |
| GREB1 | 1,781001 | 0,63158 | -1,49565 | 1,18E-40 | 2,75E-40 |
| NANOG | 0,087079 | 0,018367 | -2,24524 | 4,25E-22 | 6,53E-22 |
| EGR2 | 0,438485 | 2,725358 | 2,635845 | 2,51E-53 | 1,43E-52 |
| SCML2 | 0,752993 | 0,277201 | -1,4417 | 9,55E-45 | 2,52E-44 |
| BATF | 0,554949 | 3,80274 | 2,776613 | 5,50E-56 | 4,71E-55 |
| PPARG | 1,069522 | 3,082709 | 1,527233 | 6,64E-47 | 1,93E-46 |
| TFAP2A | 0,265306 | 1,386915 | 2,38615 | 8,62E-43 | 2,07E-42 |
| FOXA1 | 0,345968 | 1,628393 | 2,234736 | 1,13E-40 | 2,65E-40 |
| ETV1 | 0,745564 | 2,056138 | 1,463533 | 8,46E-53 | 4,46E-52 |
| SMAD2 | 3,510056 | 1,502722 | -1,22392 | 1,71E-57 | 4,94E-56 |
| KDM4C | 3,116056 | 1,400876 | -1,15339 | 3,05E-56 | 3,09E-55 |
| KLF5 | 2,183917 | 5,350314 | 1,292705 | 3,58E-47 | 1,05E-46 |
| SPIB | 0,598732 | 1,441081 | 1,267169 | 3,70E-17 | 5,17E-17 |
| HOXC9 | 0,114612 | 0,967645 | 3,077721 | 1,08E-37 | 2,32E-37 |
| FOXM1 | 0,748229 | 2,152368 | 1,524374 | 3,33E-45 | 9,05E-45 |
| FOXP3 | 0,129248 | 1,261504 | 3,286925 | 4,64E-57 | 9,08E-56 |
| E2F7 | 0,15175 | 0,553968 | 1,86811 | 4,94E-32 | 8,91E-32 |
| MAF | 1,198482 | 2,948818 | 1,298929 | 2,58E-54 | 1,65E-53 |
| XBP1 | 8,299066 | 0 | #NOME? | 1,87E-66 | 5,13E-64 |
| PRDM1 | 0,601514 | 2,260255 | 1,909814 | 2,18E-54 | 1,42E-53 |
| HOXB7 | 0,59126 | 3,274917 | 2,469593 | 7,77E-56 | 6,26E-55 |
| IRF4 | 0,151697 | 0,747841 | 2,301541 | 4,84E-33 | 9,02E-33 |
| LMNB1 | 1,197688 | 3,096839 | 1,370544 | 4,02E-56 | 3,68E-55 |
| MYB | 0,121031 | 0,717768 | 2,568138 | 3,76E-49 | 1,32E-48 |
| EMX1 | 0,305203 | 0,124323 | -1,29568 | 2,95E-31 | 5,14E-31 |
| RUNX1 | 1,361434 | 3,359013 | 1,30291 | 1,23E-49 | 4,43E-49 |
| SIX5 | 4,046002 | 1,667437 | -1,27887 | 2,22E-57 | 4,94E-56 |
| HOXC11 | 0,014867 | 0,63913 | 5,425895 | 2,30E-35 | 4,64E-35 |
| LHX2 | 0,033514 | 0,075485 | 1,171406 | 6,32E-08 | 7,69E-08 |
| GTF2I | 5,1195 | 2,173454 | -1,23601 | 1,71E-57 | 4,94E-56 |
| RYBP | 1,474147 | 3,088331 | 1,066947 | 1,80E-57 | 4,94E-56 |
| MEIS1 | 4,655516 | 2,320727 | -1,00436 | 2,11E-57 | 4,94E-56 |
| IKZF1 | 0,478871 | 1,298717 | 1,439379 | 1,22E-29 | 2,08E-29 |
| LEF1 | 0,52579 | 2,385221 | 2,181566 | 3,82E-52 | 1,72E-51 |
| GATA3 | 0,172081 | 1,718919 | 3,320346 | 2,43E-52 | 1,19E-51 |
| PAX6 | 2,09017 | 0,846015 | -1,30486 | 1,64E-39 | 3,69E-39 |
| FOSL1 | 0,747843 | 3,477071 | 2,217066 | 1,45E-49 | 5,14E-49 |
| EPO | 1,833089 | 0,191787 | -3,2567 | 2,65E-52 | 1,27E-51 |

**Supplementary Table 9** The correlation analysis of OS-related DETFs and OS-related DEICRGs.

| **TF** | **ICRGs** | **cor** | **pvalue** | **Regulation** |
| --- | --- | --- | --- | --- |
| SPDEF | MAPK12 | -0,40207 | 2,90E-08 | Negative |
| SPDEF | PAK1 | 0,407693 | 1,78E-08 | Postive |
| SPDEF | PAK3 | -0,57492 | 5,84E-17 | Negative |
| SPDEF | PPP3CB | -0,57832 | 3,48E-17 | Negative |
| SPDEF | MYD88 | 0,421196 | 5,29E-09 | Postive |
| PPARG | EML4 | 0,435454 | 1,38E-09 | Postive |
| PPARG | MAPK12 | -0,49423 | 2,73E-12 | Negative |
| PPARG | KRAS | 0,457746 | 1,50E-10 | Postive |
| PPARG | MAPK11 | -0,49385 | 2,85E-12 | Negative |
| PPARG | PAK1 | 0,513892 | 2,57E-13 | Postive |
| PPARG | PAK3 | -0,55464 | 1,15E-15 | Negative |
| PPARG | PPP3CB | -0,58324 | 1,62E-17 | Negative |
| PPARG | MYD88 | 0,669871 | 2,09E-24 | Postive |
| KLF5 | EML4 | 0,610952 | 1,72E-19 | Postive |
| KLF5 | FYN | -0,41988 | 5,97E-09 | Negative |
| KLF5 | NRAS | 0,427475 | 2,95E-09 | Postive |
| KLF5 | LYN | 0,448177 | 3,97E-10 | Postive |
| KLF5 | MAPK12 | -0,68056 | 2,02E-25 | Negative |
| KLF5 | YES1 | 0,510583 | 3,86E-13 | Postive |
| KLF5 | KRAS | 0,547251 | 3,24E-15 | Postive |
| KLF5 | MAPK11 | -0,63558 | 2,04E-21 | Negative |
| KLF5 | PAK1 | 0,634539 | 2,48E-21 | Postive |
| KLF5 | PAK3 | -0,62961 | 6,19E-21 | Negative |
| KLF5 | PPP3CB | -0,51946 | 1,28E-13 | Negative |
| KLF5 | MYD88 | 0,620747 | 3,09E-20 | Postive |
| KLF5 | PIK3CB | 0,540062 | 8,66E-15 | Postive |

**Supplementary Table 10** The correlation analysis of PFS-related DETFs and PFS-related DEICRGs.

| **TF** | **ICRGs** | **cor** | **p-value** | **Regulation** |
| --- | --- | --- | --- | --- |
| SPDEF | MAPK12 | -0,40207 | 2,90E-08 | Negative |
| SPDEF | PAK3 | -0,57492 | 5,84E-17 | Negative |
| SPDEF | PPP3CB | -0,57832 | 3,48E-17 | Negative |
| SPDEF | MYD88 | 0,421196 | 5,29E-09 | Postive |
| BATF | NFKBIE | 0,461307 | 1,03E-10 | Postive |
| BATF | PAK3 | -0,55398 | 1,26E-15 | Negative |
| BATF | PPP3CB | -0,49611 | 2,19E-12 | Negative |
| BATF | MYD88 | 0,530805 | 2,97E-14 | Postive |
| PPARG | PIK3R3 | -0,4186 | 6,71E-09 | Negative |
| PPARG | EML4 | 0,435454 | 1,38E-09 | Postive |
| PPARG | BATF | 0,538385 | 1,09E-14 | Postive |
| PPARG | MAPK12 | -0,49423 | 2,73E-12 | Negative |
| PPARG | KRAS | 0,457746 | 1,50E-10 | Postive |
| PPARG | MAPK11 | -0,49385 | 2,85E-12 | Negative |
| PPARG | PAK3 | -0,55464 | 1,15E-15 | Negative |
| PPARG | PPP3CB | -0,58324 | 1,62E-17 | Negative |
| PPARG | MYD88 | 0,669871 | 2,09E-24 | Postive |
| KLF5 | EML4 | 0,610952 | 1,72E-19 | Postive |
| KLF5 | FYN | -0,41988 | 5,97E-09 | Negative |
| KLF5 | BATF | 0,437889 | 1,09E-09 | Postive |
| KLF5 | NRAS | 0,427475 | 2,95E-09 | Postive |
| KLF5 | LYN | 0,448177 | 3,97E-10 | Postive |
| KLF5 | MAPK12 | -0,68056 | 2,02E-25 | Negative |
| KLF5 | YES1 | 0,510583 | 3,86E-13 | Postive |
| KLF5 | KRAS | 0,547251 | 3,24E-15 | Postive |
| KLF5 | MAPK11 | -0,63558 | 2,04E-21 | Negative |
| KLF5 | PAK3 | -0,62961 | 6,19E-21 | Negative |
| KLF5 | PPP3CB | -0,51946 | 1,28E-13 | Negative |
| KLF5 | MYD88 | 0,620747 | 3,09E-20 | Postive |
| KLF5 | PIK3CB | 0,540062 | 8,66E-15 | Postive |

**Supplementary Figure 1** Heatmap (A) and volcano plot (B) of 124 DEICRGs between PAAD and normal pancreatic tissues.


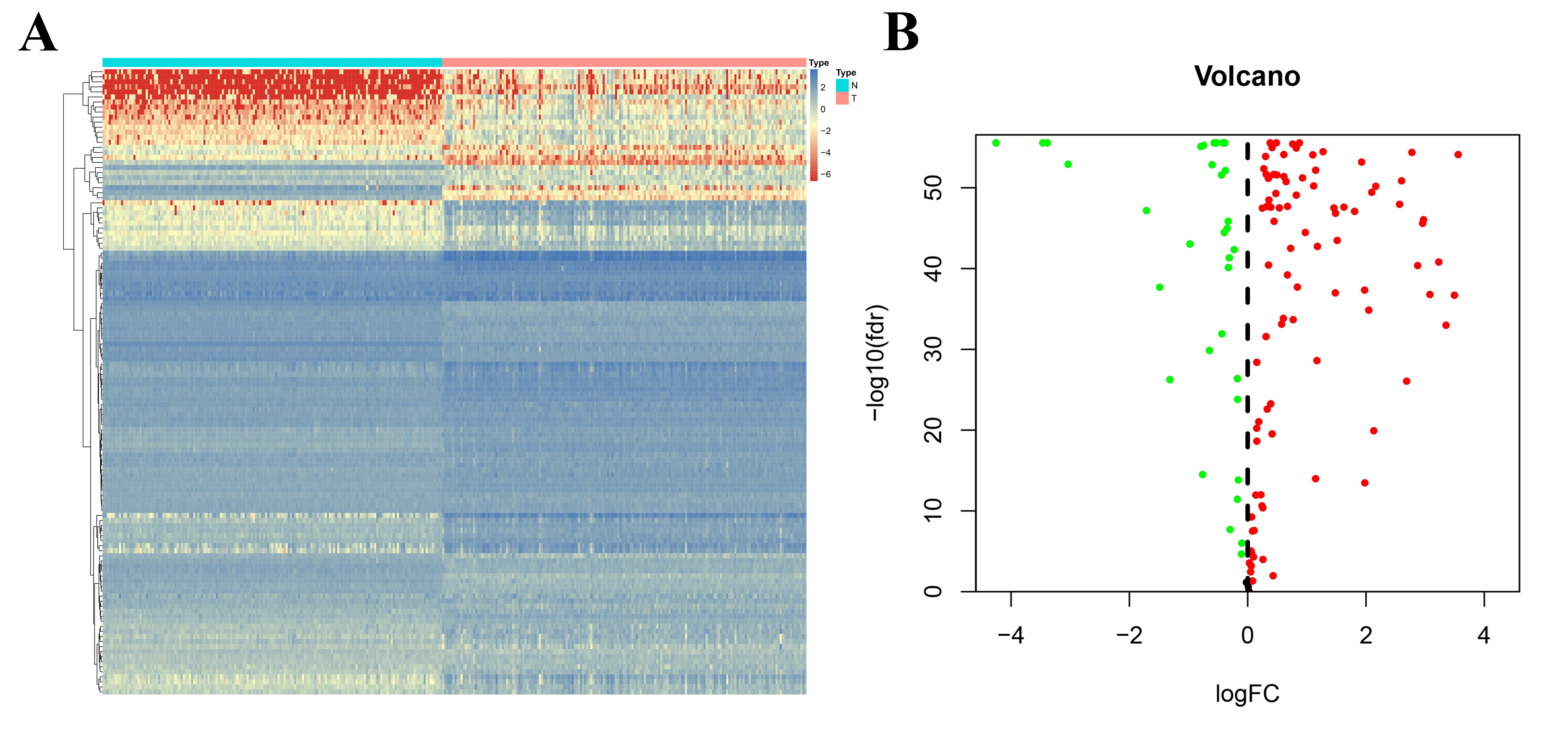


**Supplementary Figure 2** External validation of the OS-related signature (A) and PFS-related signature (B) in the ICGC database.


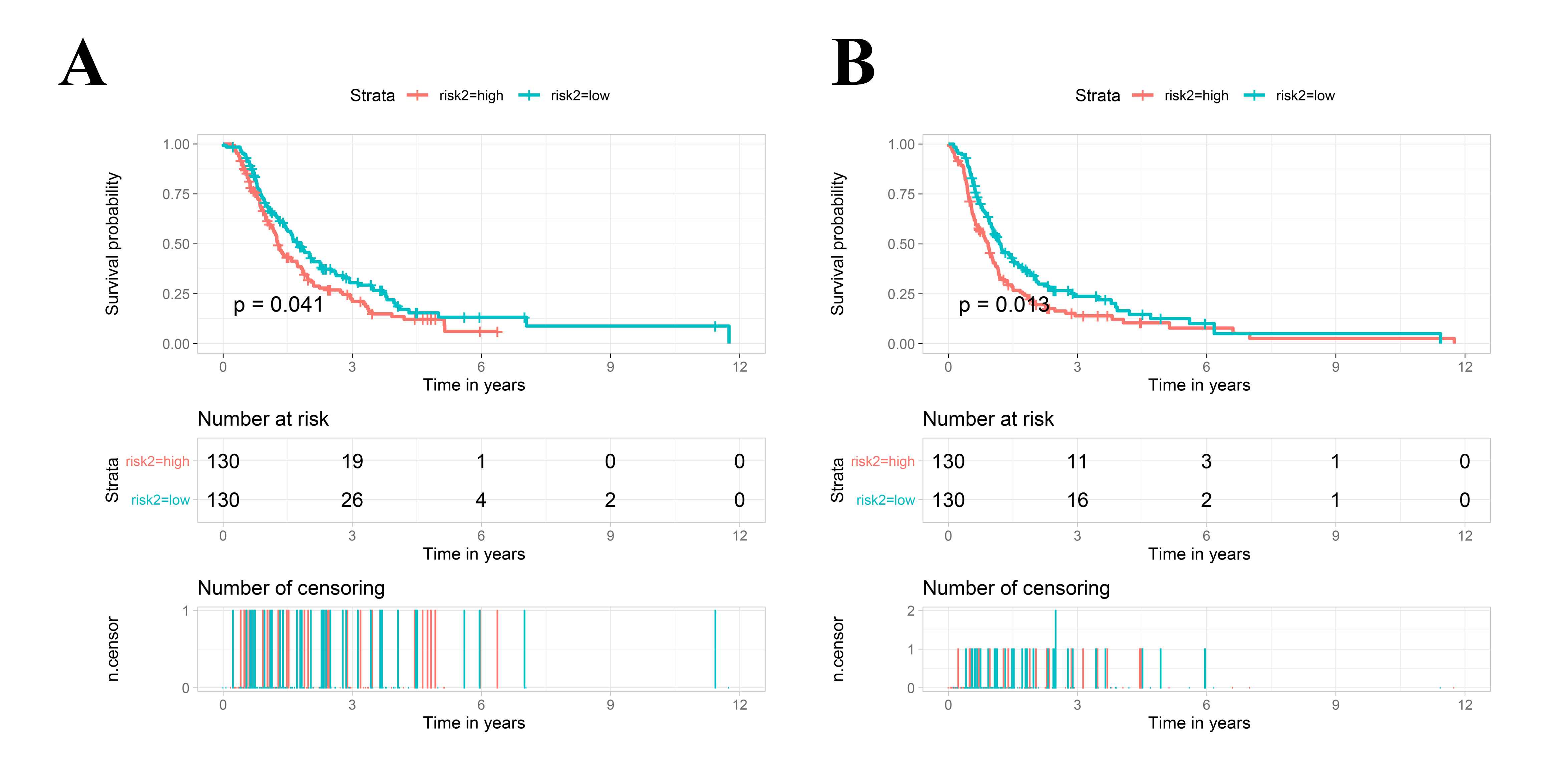


**Supplementary Figure 3** The heatmaps of 920 DEGs (A) and 492 DEGs (D) between high- and low-risk groups in the OS-related signature and PFS-related signature.


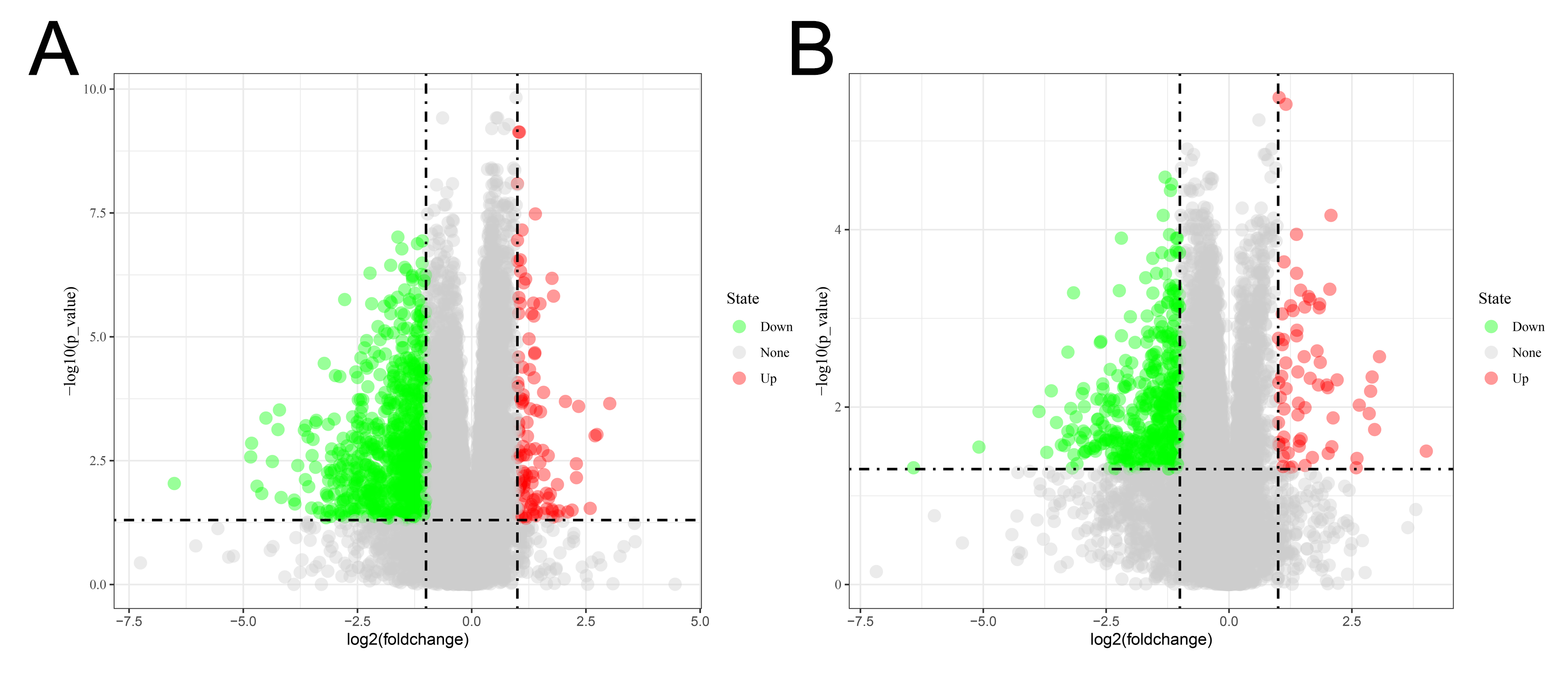


**Supplementary Figure 4** Mutation state of hub genes and infiltration level of six immune cells.
